# UBE2C contributes to malignant phenotypes in clear cell renal cell carcinoma *via* cell cycle and apoptosis regulation

**DOI:** 10.7717/peerj.21436

**Published:** 2026-06-18

**Authors:** Tianzi Qin, Jie Liang, Wentong Ya, Yi Long, Jichao Wu, Dongdong Meng, Fu Gan

**Affiliations:** 1 Department of Urinary Surgery, Affiliated Hospital of Youjiang Medical University for Nationalities, Baise, GuangXi, China; 2 Life Science and Clinical Medicine Research Center, Baise, GuangXi, China; 3 Clinical Medical College, Youjiang Medical University for Nationalities, Baise, GuangXi, China

**Keywords:** Clear cell renal cell carcinoma, Prognosis, Cell cycle, Mendelian randomization, UBE2C

## Abstract

**Background:**

Clear cell renal cell carcinoma (ccRCC) is characterized by aggressive behavior and poor prognosis. Ubiquitin-conjugating enzyme E2C (UBE2C), a key component of the ubiquitin-proteasome system, has been implicated in tumorigenesis; however, its role in ccRCC remains unclear. This study aimed to systematically investigate the clinical relevance, genetic associations, and biological functions of UBE2C in ccRCC.

**Methods:**

Multi-omics analyses of The Cancer Genome Atlas Kidney Renal Clear Cell Carcinoma (TCGA-KIRC) and Gene Expression Omnibus (GEO) datasets were performed to evaluate UBE2C expression and prognostic significance. Mendelian randomization (MR) analysis was conducted to explore potential genetic associations. Experimental validation was performed using clinical specimens and ccRCC cell lines, including quantitative reverse transcriptase polymerase chain reaction (qRT-PCR), immunohistochemistry (IHC), immunofluorescence (IF), functional assays (CCK-8, colony formation, Transwell, and xenograft models), rescue experiments, and western blot analysis. Gene set enrichment analysis (GSEA) and flow cytometry were used to investigate potential mechanisms.

**Results:**

UBE2C expression was significantly upregulated in ccRCC tissues and was associated with advanced stage, metastasis, high tumor grade, and poor survival (HR = 2.25, 95% CI [1.64–3.09]; *P* < 0.001). MR analysis suggested a genetic association between elevated serum UBE2C levels and increased ccRCC risk (IVW OR = 1.418, *P* = 0.010). Functional assays demonstrated that UBE2C knockdown inhibited proliferation, migration, and invasion while promoting apoptosis, whereas UBE2C overexpression enhanced malignant phenotypes. GSEA indicated enrichment of cell cycle and apoptosis-related pathways, which was supported by flow cytometry showing G0/G1 arrest and increased apoptosis in UBE2C-silenced cells. Rescue experiments further showed that re-expression of UBE2C restored proliferative and invasive capacities. Western blot analysis revealed that UBE2C modulated cell cycle and apoptosis-related proteins.

**Conclusions:**

UBE2C was upregulated in ccRCC and was associated with aggressive clinicopathological features and poor prognosis. Functional, rescue, and protein-level analyses suggested that UBE2C may contribute to malignant phenotypes through regulation of cell cycle progression and apoptosis. These findings indicate that UBE2C may serve as a potential prognostic biomarker and therapeutic target in ccRCC, although further mechanistic studies are required.

## Introduction

Renal cell carcinoma (RCC) is the most common malignant tumor of the kidney, with clear cell renal cell carcinoma (ccRCC) accounting for approximately 75–80% of all cases (Makino et al., 2022). Despite advances in surgical resection and the development of targeted therapies and immunotherapies, patients with advanced or metastatic ccRCC still have poor outcomes, with 5-year survival rates below 10% (Bhanegaonkar et al., 2021; Su et al., 2023). The pronounced molecular heterogeneity of ccRCC contributes to tumor aggressiveness and therapeutic resistance, highlighting the urgent need to identify novel molecular drivers and actionable therapeutic targets (Schiavoni et al., 2023; Xu et al., 2024).

The ubiquitin-proteasome system (UPS) plays a crucial role in maintaining cellular homeostasis by regulating protein degradation in a temporally and spatially controlled manner. Through this mechanism, the UPS governs essential biological processes including cell cycle progression, apoptosis, DNA repair, and signal transduction (Din et al., 2024; Hitora & Tsukamoto, 2025). Dysregulation of the UPS has been implicated in the development and progression of multiple cancers, including ccRCC (Guo et al., 2021). Among UPS components, ubiquitin-conjugating enzyme E2C (UBE2C) has attracted considerable attention due to its role in the ubiquitin-mediated degradation of mitotic regulators such as cyclin B1 and securin (Chen & Wang, 2021; Liu et al., 2019), thereby ensuring proper mitotic progression and genomic stability (Domentean et al., 2023).

UBE2C overexpression has been reported in several malignancies, including breast, lung, bladder, and colorectal cancers, where it is associated with tumor progression and poor prognosis (Dastsooz et al., 2019). Functional studies suggest that UBE2C may promote tumor growth by enhancing proliferation, suppressing apoptosis, and facilitating invasion (Zhang et al., 2023). However, the biological role of UBE2C in ccRCC remains incompletely understood. In particular, the clinical relevance, genetic associations, and functional mechanisms of UBE2C in ccRCC have not been systematically evaluated using integrated genomic and experimental approaches.

To address these gaps, this study employed a multidisciplinary strategy to investigate the role of UBE2C in ccRCC. Multi-omics analyses of The Cancer Genome Atlas Kidney Renal Clear Cell Carcinoma (TCGA-KIRC) and Gene Expression Omnibus (GEO) datasets were performed to evaluate UBE2C expression patterns and prognostic significance. Bidirectional Mendelian randomization (MR) analysis was conducted to explore potential genetic associations between UBE2C and ccRCC risk. Experimental validation was further performed using clinical specimens and ccRCC cell lines, including molecular profiling and functional assays. In addition, gene set enrichment analysis (GSEA) and flow cytometry were used to explore biological pathways potentially regulated by UBE2C.

## Materials and Methods

### Data acquisition and processing

RNA-seq FPKM data and corresponding clinical information of ccRCC patients were retrieved from The Cancer Genome Atlas (TCGA) portal (https://portal.gdc.cancer.gov/). The dataset consisted of 539 tumor samples and 72 adjacent normal samples (Saad et al., 2022). Additionally, the microarray dataset GSE53757 was downloaded from the Gene Expression Omnibus (GEO) database (https://www.ncbi.nlm.nih.gov/geo/), which comprised 72 ccRCC tissues and 72 normal kidney tissues (Wang et al., 2023a). Probe IDs were annotated to gene symbols based on the platform annotation files. When multiple probes corresponded to the same gene, the average expression value was calculated and used for further analysis.

### Differential expression and statistical analysis

Student’s t-test was used to compare UBE2C expression between ccRCC and normal tissues. For paired samples in TCGA, a paired Student’s t-test was used. Statistical significance was defined as two-tailed *P* < 0.05. All analyses were performed in R (v4.2.1; R Core Team, 2022), and data were visualized with the ggplot2 package (Lv et al., 2023).

### Survival and prognostic analysis

TCGA-KIRC patients were divided into high- and low-UBE2C expression groups based on the median expression level. We used Kaplan-Meier curves to compare overall survival (OS), with log-rank tests for significance. Univariate and multivariate Cox proportional hazards models were used to evaluate the prognostic value of UBE2C, along with clinicopathological factors (age, gender, tumor, node, metastasis (TNM) stage, grade). Results are reported as hazard ratios (HR) with 95% confidence intervals (CI). Receiver operating characteristic (ROC) curves were generated, and the area under the curve (AUC) was calculated to assess UBE2C’s diagnostic performance.

### Mendelian randomization analysis

Serum UBE2C genome-wide association study (GWAS) data (ID: ebi-a-GCST90087066) and ccRCC GWAS data (ID: C3_KIDNEY_CLEAR_CELL_CARCINOMA_EXALLC) were obtained from the MRC Integrative Epidemiology Unit (IEU) GWAS database (https://gwas.mrcieu.ac.uk). Both datasets were from European populations (2021): serum UBE2C data included 5,365 samples and 1,048,575 single nucleotide polymorphisms (SNPs), while ccRCC data included 315,137 samples and 21,303,856 SNPs.

Instrumental variable (IV) selection: Independent SNPs significantly associated with serum UBE2C levels (*P* < 5 × 10^−8^) were selected as IVs. Similarly, ccRCC-associated SNPs were extracted from the respective GWAS. Linkage disequilibrium (LD) clumping (r^2^ < 0.001, window size = 10,000 kb) was performed to ensure IV independence. SNP strength was verified using the F-statistic (F > 10).

MR analysis: Bidirectional two-sample MR was performed. Inverse-variance weighted (IVW) regression was the main method, supplemented by MR-Egger, weighted median, simple mode, and weighted mode for sensitivity analysis. Causal effects are reported as odds ratios (OR) with 95% CI.

Sensitivity Analysis: Heterogeneity was assessed with Cochran’s Q statistic. Horizontal pleiotropy was evaluated using MR-Egger intercept tests and MR-PRESSO global tests. Leave-one-out analysis was used to determine the influence of individual SNPs (Skrivankova et al., 2021).

### Patient tissue collection

Fresh-frozen ccRCC tissues and paired adjacent normal tissues (≥1 cm from the tumor margin) were collected from 30 patients who underwent radical or partial nephrectomy at our hospital. The sample size was determined based on sample availability during the study period and precedent from previously published ccRCC tissue validation studies, which commonly include 20–40 paired samples. A cohort of 30 paired tissues was therefore considered sufficient for experimental validation. The study was conducted in line with the Declaration of Helsinki and approved by the Youjiang Medical University for Nationalities Institutional Review Board (Approval No: 2025JJH140083). Written informed consent was obtained from all participants. Tissues were immediately snap-frozen in liquid nitrogen and stored at −80 °C for later RNA/protein extraction. An additional set of paired formalin-fixed paraffin-embedded (FFPE) tissues from the same patients was used for immunohistochemistry (IHC) and immunofluorescence (IF).

### Quantitative reverse transcription polymerase chain reaction

Total RNA was extracted from tissues and cell lines using TRIzol™ Reagent (Cat. No.: 15596026; Thermo Fisher Scientific, Waltham, MA, USA) following the manufacturer’s instructions. RNA purity was checked with a NanoDrop™ One spectrophotometer (Thermo Fisher Scientific, Waltham, MA, USA), with A260/A280 ratios between 1.9 and 2.1. After DNase I treatment, 1 µg of RNA was reverse-transcribed into cDNA using ToloScript™ All-in-one RT EasyMix (Cat. No.: R0101; TOLOBIO, Shanghai, China) in a 20 µL reaction system (50 °C for 15 min; 85 °C for 5 s), and cDNA was stored at −80 °C. Quantitative reverse transcriptase polymerase chain reaction (qRT-PCR) was performed on a LightCycler® 96 system (Roche, Basel, Switzerland) with 20 µL reactions containing 2 µL cDNA, 10 µL PowerUp™ SYBR™ Green Master Mix (Cat. No.: A25742; Thermo Fisher Scientific, Waltham, MA, USA), and 0.4 μM gene-specific primers (UBE2C or GAPDH). The thermal cycling protocol was: uracil-DNA glycosylase (UDG) activation at 50 °C for 2 min; initial denaturation at 95 °C for 2 min; 40 cycles of denaturation at 95 °C for 15 s and annealing/extension at 60 °C for 15 s (modified from the original separate extension step for SYBR Green chemistry). Melting curve analysis (95 °C → 60 °C → 95 °C, 15 s per step) confirmed specific single-peak amplification. Primer sequences were designed using NCBI Primer-BLAST to generate approximately 100 bp amplicons. The sequences are listed in Table S1. All primers were synthesized by Sangon Biotech (Shanghai, China). Primer specificity was confirmed by melting-curve analysis. Relative expression was calculated using the 2^(−ΔΔCT)^ method with GAPDH as the internal control. PCR efficiency was validated (slope: −3.39 to −3.1; R^2^ ≥ 0.98). The limit of detection (LOD) was 2.5 (95% CI). Technical replicates with >20% deviation from median Ct values were excluded, and data were derived from 3–20 biological replicates analyzed in triplicate.

### Immunohistochemistry

Deparaffinized and rehydrated 4 μm ccRCC sections were subjected to antigen retrieval in citrate buffer (pH 6.0) using microwave heating. Endogenous peroxidase activity was blocked with 3% H_2_O_2_ for 15 min. After blocking with 10% normal goat serum, sections were incubated overnight at 4 °C with an anti-UBE2C antibody (1:200, Cat. No.: ab252940; Abcam, Cambridge, UK), followed by incubation with an HRP-conjugated secondary antibody (ZSGB-BIO, Beijing, China) for 1 h at room temperature (RT) and DAB development with hematoxylin counterstaining. Two blinded pathologists independently scored staining intensity (0: negative; 1: weak; 2: moderate; 3: strong) and positive cell percentage (0: 0%; 1: 1–25%; 2: 26–50%; 3: 51–75%; 4: 76–100%). High expression was defined as a total score (intensity × percentage) ≥4.

### Immunofluorescence

For IF staining, tissue sections or cultured cells on coverslips were fixed in 4% paraformaldehyde for 15 min. Cell membranes were permeabilized with 0.1% Triton X-100 for 10 min, followed by blocking with 5% bovine serum albumin (BSA) for 1 h. Samples were then incubated overnight at 4 °C with a primary antibody against UBE2C (1:100; Abcam, Cambridge, UK). Next, samples were incubated for 1 h at RT in the dark with an Alexa Fluor 488-conjugated secondary antibody (1:500; Invitrogen, Waltham, MA, USA). Nuclei were counterstained with DAPI (Solarbio, Beijing, China). Slides were imaged using a Nikon fluorescence microscope (Tokyo, Japan).

### Cell lines and culture conditions

The ccRCC cell lines 786-O, 769-P, ACHN, and Caki-1, as well as the human embryonic kidney cell line HK-2, were bought from ProCell Corporation (Wuhan, China). The four ccRCC cell lines were selected to represent distinct biological characteristics. 786-O and 769-P are VHL-deficient lines with high proliferative capacity, ACHN exhibits strong migratory and invasive potential, and Caki-1 provides additional biological heterogeneity. The use of multiple cell lines with different genetic backgrounds enhances the robustness of the functional analyses. All cell lines used in this study were authenticated by short tandem repeat (STR) profiling. Cell culture procedures were performed in accordance with relevant biosafety regulations and ethical guidelines. HK-2 and ACHN cells were cultured in Minimum Essential Medium (OriCell, Shanghai, China). Caki-1 cells were grown in McCoy’s 5A medium (OriCell, Shanghai, China), while 786-O and 769-P cells were cultured in RPMI-1640 medium (OriCell, Shanghai, China). All basal media were supplemented with 10% fetal bovine serum (OriCell, Shanghai, China) and 1% penicillin/streptomycin (Solarbio, Beijing, China). Cells were maintained in a humidified incubator at 37 °C with 5% CO_2_. All cell lines used in this study were regularly tested for mycoplasma contamination using the mycoplasma detection kit (Vazyme, Nanjing, China), and confirmed to be free of mycoplasma contamination before experimentation.

### Lentiviral transduction and establishment of stable cell lines

For UBE2C knockdown, three UBE2C-targeting short hairpin RNAs (shRNAs) and a non-targeting control (NC) were cloned into the pLKO.1 vector. For overexpression, full-length UBE2C cDNA was inserted into the pCDH-CMV-MCS-EF1-Puro vector. Lentiviruses were packaged in 293T cells using Lipofectamine 3000 (Invitrogen, Waltham, MA, USA) with psPAX2 and pMD2.G plasmids. Viral supernatants (collected at 48 and 72 h post-transfection) were filtered and used to infect 786-O, ACHN, and 769-P cells in the presence of 8 µg/mL polybrene (Sigma-Aldrich, St. Louis, MO, USA). Stable cell lines were selected with 2 µg/mL puromycin (Sigma-Aldrich, St. Louis, MO, USA) for ≥14 days, and transduction efficiency was confirmed by qRT-PCR before functional assays.

### Cell counting kit-8 proliferation assay

Cells were seeded in 96-well plates at a density of 2 × 10^3^ cells per well. At 0, 24, 48, 72, and 96 h after seeding, 10 µL of Cell counting kit-8 (CCK-8) reagent (UElandy, Shanghai, China) was added to each well. Plates were incubated at 37 °C for 2 h, and absorbance at 450 nm was measured using a microplate reader (BioTek, Winooski, VT, USA). Each experimental condition included six replicate wells, and the experiment was repeated three times independently.

### Colony formation assay

Cells were trypsinized and seeded into 6-well plates at a low density (500 cells per well). Culture medium was changed every 3–4 days. After 14 days of incubation, colonies were washed twice with PBS, fixed with methanol for 15 min, and stained with 0.1% crystal violet (Beyotime, Shanghai, China) for 20 min. Colonies containing >50 cells were counted manually under a microscope. The experiment was done in triplicate.

### Transwell migration and invasion assays

Cell migration and invasion were assessed using 24-well Transwell chambers with 8.0 μm pores (Corning, Corning, NY, USA). For the migration assay, 5 × 10^4^ cells suspended in 200 µL serum-free medium were seeded into the upper chamber. For the invasion assay, the upper chamber was pre-coated with 50 µL Matrigel (diluted 1:8 in serum-free medium; Corning) and allowed to solidify at 37 °C for 4 h. The lower chamber was filled with 600 µL medium supplemented with 20% fetal bovine serum (FBS) as a chemoattractant. After 24–48 h of incubation, non-migrated/non-invaded cells on the upper membrane were gently removed with a cotton swab. Cells on the lower surface were fixed with methanol, stained with 0.1% crystal violet, and imaged under an inverted microscope (Nikon, Tokyo, Japan). Five random fields were counted per membrane. Each experiment was repeated three times.

### Flow cytometry for apoptosis and cell cycle

Cell cycle analysis was performed using a Cell Cycle Staining Kit (Multi Sciences, Shanghai, China) according to the manufacturer’s protocol. Briefly, 1 × 10^6^ cells were harvested, washed with PBS, and centrifuged to remove the supernatant. Then, 1 mL of DNA Staining Solution and 10 µL of Permeabilization Solution were added, followed by vortex mixing for 5–10 s. Cells were incubated at room temperature for 30 min and analyzed by flow cytometry.

For apoptosis analysis, cells were stained using an Annexin V-FITC/PI Apoptosis Kit (Multi Sciences, Shanghai, China). Cells were harvested using Accutase, washed with pre-cooled PBS, and 1 × 10^5^ cells were resuspended in 100 µL of 1× binding buffer. Subsequently, 5 µL Annexin V-FITC and 10 µL propidium iodide (PI) were added, followed by gentle vortex mixing and incubation at room temperature for 5 min in the dark.

Data acquisition was performed using a flow cytometer (Thermo Fisher Scientific, Waltham, MA, USA), and at least 10,000 events were collected for each sample. Annexin V-FITC (Ex = 488 nm, Em = 530 nm) and PI (Ex = 535 nm, Em = 615 nm) signals were detected in their respective channels. Data were analyzed using FlowJo software (version 10.8.1; BD Biosciences, San Jose, CA, USA). Early apoptotic cells were defined as Annexin V-positive/PI-negative, and late apoptotic cells were defined as Annexin V-positive/PI-positive. For cell-cycle analysis, cell populations in G0/G1, S, and G2/M phases were quantified using the Watson pragmatic model in FlowJo software. All experiments were performed in triplicate.

### *In vivo* transplants tumor model

Four-week-old male BALB/c nude mice (*n* = 12) were purchased from Vital River Laboratory Animal Technology Co., Ltd. (Beijing, China). All mice were housed under specific pathogen-free (SPF) conditions in individually ventilated cages (IVC) with controlled environmental parameters: a 12 h light/dark cycle, temperature maintained at 22 ± 1 °C, and relative humidity at 55 ± 5%. SPF-grade standard laboratory chow and autoclaved sterile water were provided *ad libitum*. To promote species-specific natural behaviors and reduce stress, environmental enrichment materials were supplemented, including nesting materials, cardboard tubes, and wooden chew blocks. Twice-weekly health monitoring was performed by trained staff to assess signs of distress or abnormal conditions. Prior to experimental interventions, the sample size was determined based on previous studies investigating ACHN cell-derived xenograft tumor models in nude mice, which have demonstrated that a sample size of six mice per group is sufficient to detect consistent intergroup differences in tumor growth parameters (*e.g*., tumor volume and weight). This sample size also adheres to the minimum requirements for statistical validity in *in vivo* tumorigenicity assays, consistent with the ARRIVE guidelines 2.0 and commonly used sample sizes in xenograft tumor studies (Festing & Altman, 2002; Percie du Sert et al., 2020). Subsequently, the 12 mice were randomly divided into two groups (*n* = 6 per group) using a random number table method (numbered sequentially, sorted by random numbers, first 6 as control group injected with ACHN cells stably transfected with empty lentiviral vector and the rest as experimental group injected with ACHN cells stably transfected with lentiviral shUBE2C), with body weight and activity status measured to confirm no significant intergroup difference (*P* > 0.05) for grouping rationality. Before injection, the stably transfected cells of both groups were digested with trypsin, centrifuged (1000 rpm, 5 min), washed twice with sterile PBS, and resuspended in PBS/Matrigel (1:1) to adjust the cell concentration to 5 × 10^6^ cells/100 µL, which was kept on ice to maintain cell viability. Subsequently, the cell suspensions of the two groups were subcutaneously injected into the posteromedial region of the right axilla of corresponding mice (*n* = 6 per group) without analgesia (procedure < 2 min with 25G needles, complying with IACUC Category B guidelines for transient, minimal discomfort). To mitigate potential confounders, we used alternating housing, randomized injections *via* a unified random number table, investigator blinding for tumor assessment and health monitoring, and standardized all procedures while excluding confounding variables (*e.g*., handling time variations). Tumor volume was measured every 3 days using digital calipers. The volume was calculated using the formula: Volume = length × width^2^/2. During each measurement, mice were anesthetized with isoflurane (3% for induction, 1.5% for maintenance *via* a nose cone) to minimize restraint stress and ensure accurate measurements. Predefined humane endpoints for euthanasia were established as follows: >20% body weight loss within 48 h, ulcerated tumors covering more than 10% of the body surface area, or severe mobility impairment. None of the animals reached these humane endpoints prior to the scheduled experimental termination. At 4 weeks post-injection, all mice were euthanized by cervical dislocation after confirming unconsciousness *via* the toe pinch reflex test. Tumors were immediately excised, weighed, and processed for subsequent experiments: samples designated for immunohistochemistry (IHC) were fixed in 4% paraformaldehyde (PFA), while the remaining samples were snap-frozen for future analyses. All animal experimental procedures were approved by the Animal Ethics Committee of Youjiang Medical University for Nationalities (Approval No.: 2023101302) and strictly complied with the ARRIVE Guidelines 2.0 (Animal Research: Reporting of *In Vivo* Experiments).

### Rescue experiments

To further validate the functional role of UBE2C, rescue experiments were performed. ACHN cells were divided into four groups: NC + vector, shUBE2C + vector, NC + UBE2C, and shUBE2C + UBE2C. UBE2C knockdown cells were transfected with UBE2C overexpression plasmids using Lipofectamine 3000 (Invitrogen, Waltham, MA, USA) according to the manufacturer’s instructions. After 48 h, cells were subjected to colony formation assays, Transwell migration and invasion assays, and western blot analysis. Rescue efficiency was confirmed by detecting UBE2C protein expression. These experiments were performed to determine whether re-expression of UBE2C could reverse the inhibitory effects induced by UBE2C knockdown.

### Western blot analysis

Total protein was harvested following cell lysis and clarified by centrifugation at 14,000× *g* for 10 min at 4 °C. Protein samples were separated using SDS-PAGE (EpiZyme, Shanghai, China) and subsequently transferred onto polyvinylidene fluoride (PVDF) membranes (Merck Millipore, Burlington, MA, USA). The membranes were blocked with 10% skimmed milk in TBST for 1 h at room temperature and then incubated with primary antibodies overnight at 4 °C with gentle agitation. After washing with PBS, membranes were incubated with HRP-conjugated anti-rabbit secondary antibody (1:7500; Proteintech, Wuhan, China) for 1 h at room temperature. Protein bands were visualized using enhanced chemiluminescence reagents (EpiZyme, Shanghai, China) and captured with an AI600 imaging system (GE Healthcare, Chicago, IL, USA). Band intensities were quantified using ImageJ software (version 1.8.0). Details of the antibodies used in this study are provided in Table S2.

### Gene set enrichment analysis

GSEA (version 4.3.2) was performed using the GEO dataset GSE53757. The gene expression profile was ranked based on correlation with UBE2C expression. The Hallmark gene sets “HALLMARK_APOPTOSIS” and “HALLMARK_G2M_CHECKPOINT” (representing the cell cycle) were obtained from the Molecular Signatures Database (MSigDB; http://www.gsea-msigdb.org/gsea/msigdb). The number of permutations was set to 1,000. A normalized enrichment score (NES) with a false discovery rate (FDR) q-value < 0.25 was considered statistically significant (Wang et al., 2024).

### Statistical analysis

All statistical analyses were performed using GraphPad Prism (version 9.0; GraphPad Software, San Diego, CA, USA) and R software (version 4.2.1). Data are presented as mean ± standard deviation (SD) from at least three independent biological experiments. Comparisons between two groups were conducted using unpaired or paired Student’s t-tests, as appropriate. Differences among multiple groups were analyzed using one-way analysis of variance (ANOVA) followed by Tukey’s *post hoc* test. Categorical variables were analyzed using the chi-square test or Fisher’s exact test. Survival analysis was performed using the Kaplan-Meier method with the log-rank test. Cox proportional hazards regression models were used for univariate and multivariate analyses. The proportional hazards assumption was evaluated using Schoenfeld residuals. Multiple testing correction was performed using the Benjamini-Hochberg false discovery rate (FDR) method where applicable. Flow cytometry data were analyzed from three independent experiments and compared using unpaired Student’s t-test or one-way analysis of variance (ANOVA), as appropriate. Statistical comparisons for rescue experiments involving four groups were performed using one-way ANOVA followed by Tukey’s multiple comparisons test. Normality of data distribution was assessed before applying parametric tests. All statistical tests were two-sided, and *P* < 0.05 was considered statistically significant.

## Results

### Bioinformatic analysis of UBE2C expression and prognostic value

**Figure 1:**
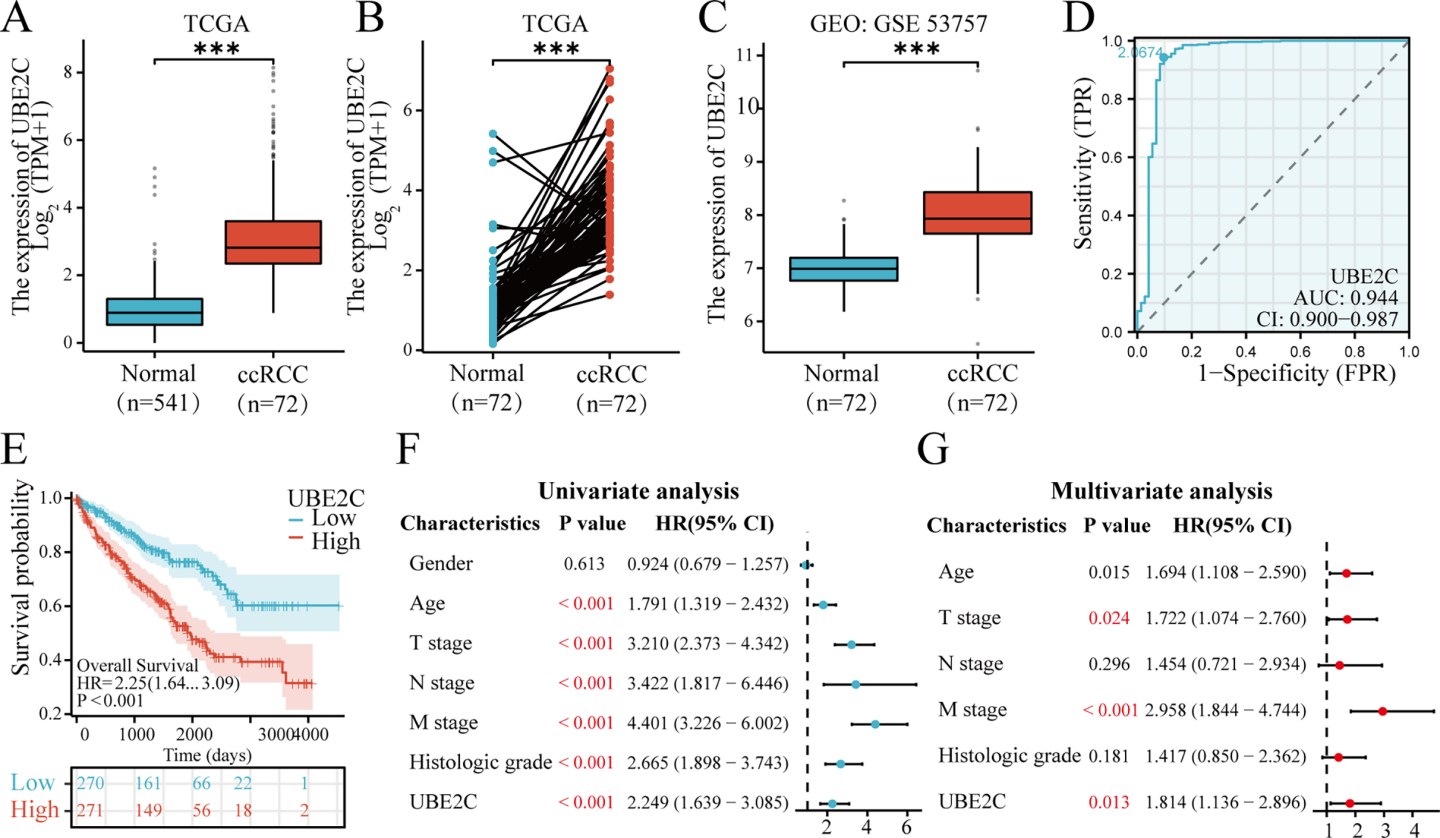
Analysis of UBE2C expression and prognostic value in ccRCC based on bioinformatics. (A) The differential expression of UBE2C was analyzed between 541 cases of ccRCC and 72 cases of normal tissues in the TCGA database. (B) The differential expression of UBE2C was examined between 72 cases of ccRCC and their corresponding adjacent tissues in the TCGA database. (C) The differential expression of UBE2C was investigated between 72 cases of ccRCC and 72 cases of normal renal tissues in the GEO database. (D) The ROC curve was used to demonstrate the diagnostic value of UBE2C in ccRCC. (E) The overall survival (OS) curve was derived from the TCGA database. (F) Univariate cox analysis was performed based on the TCGA ccRCC data. (G) Multivariate cox analysis was conducted based on the TCGA ccRCC data. ***P < 0.001.

Analysis of the TCGA-KIRC dataset showed notably higher UBE2C mRNA expression in ccRCC tissues than in normal kidney tissues (t = 15.592, *P* < 0.001; Fig. 1A). This finding was confirmed by paired sample analysis, which showed that UBE2C expression was significantly upregulated in tumor tissues compared to matched adjacent normal tissues (t = 13.395, *P* < 0.001; Fig. 1B). Additional validation using the GEO dataset GSE53757 further confirmed marked upregulation of UBE2C in ccRCC samples (t = 9.717, *P* < 0.001; Fig. 1C). ROC curve analysis showed an AUC of 0.944 (95% CI [0.900–0.987]) with an optimal cutoff value of 2.067 (Fig. 1D), indicating strong discriminatory ability for ccRCC. Patients were divided into high- and low-UBE2C expression groups based on the median expression level. Chi-square tests showed no significant differences in gender ( ${\chi^2}$ = 3.723, *P* = 0.054) or age ( ${\chi^2}$ = 1.155, *P* = 0.282) between the two groups. However, significant associations were found with advanced T stage ( ${\chi^2}$ = 31.761, *P* < 0.001), N stage ( ${\chi^2}$ = 9.204, *P* = 0.002), M stage ( ${\chi^2}$ = 29.112, *P* < 0.001), pathologic stage ( ${\chi^2}$ = 30.991, *P* < 0.001), and histologic grade ( ${\chi^2}$ = 34.232, *P* < 0.001; Table 1). Kaplan-Meier survival analysis showed that patients with high UBE2C expression had significantly shorter overall survival (OS) than those with low expression (HR = 2.25, 95% CI [1.64–3.09], *P* < 0.001; Fig. 1E). Univariate and multivariate Cox regression analyses further confirmed UBE2C expression as an independent prognostic factor for OS (HR = 1.814, 95% CI [1.136–2.896], *P* = 0.013), along with T stage (HR = 1.722, 95% CI [1.074–2.760], *P* = 0.024) and M stage (HR = 2.958, 95% CI [1.844–4.744], *P* < 0.001; Figs. 1F, 1G).

**Table 1:** Clinical characteristics of UBE2C expression in renal clear cell carcinoma patients based on TCGA database.

Clinical features	Sample size (*n*)	UBE2C expression		χ^2^	*P*
		Low (*n* = 270)	High (*n* = 271)		
Gender, *n* (%)					
男	354	166 (30.7%)	188 (34.8%)	3.723	0.054
女	187	104 (19.2%)	83 (15.3%)		
Age (year), *n* (%)					
<=60	269	128 (23.7%)	141 (26.1%)	1.155	0.282
>60	272	142 (26.2%)	130 (24%)		
T stages, *n* (%)					
T1 & T2	350	206 (38.1%)	144 (26.6%)	31.761	<0.001
T3 & T4	191	64 (11.8%)	127 (23.5%)		
N stages, *n* (%)					
N0	242	125 (48.5%)	117 (45.3%)	9.204	0.002
N1	16	2 (0.8%)	14 (5.4%)		
M stages, *n* (%)					
M0	429	234 (46.1%)	195 (38.4%)	29.112	<0.001
M1	79	17 (3.3%)	62 (12.2%)		
Pathological stage, *n* (%)					
Stage I & Stage II	332	198 (36.8%)	134 (24.9%)	30.991	<0.001
Stage III & Stage IV	206	72 (13.4%)	134 (24.9%)		
Histological grade, *n* (%)					
G1 & G2	250	158 (29.6%)	92 (17.3%)	34.232	<0.001
G3 & G4	283	107 (20.1%)	176 (33%)		

### Mendelian randomization analysis of the causal relationship between UBE2C and ccRCC risk

Bidirectional two-sample MR analysis was done to explore the causal role of UBE2C in ccRCC. After LD clumping and removal of weak IVs, 18 SNPs significantly associated with serum UBE2C levels and nine SNPs associated with ccRCC were retained as IVs.

**Figure 2:**
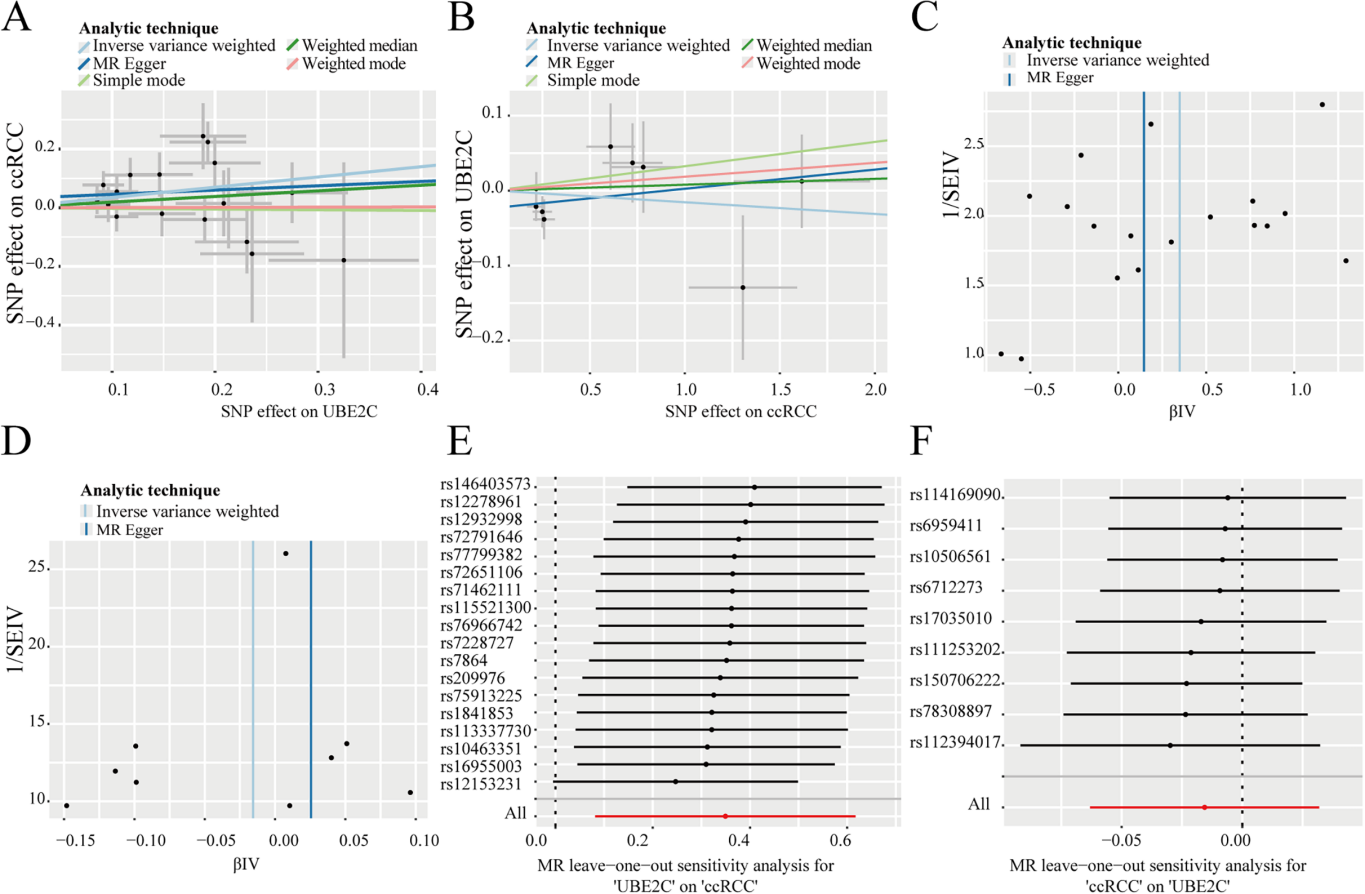
Scatter plot, funnel plot and leave-one-out sensitivity analysis of MR analysis results. (A, B) Scatter plots depicting the causal relationship between UBE2C and ccRCC in MR analyses: (A) UBE2C → ccRCC; (B) ccRCC → UBE2C. (C, D) Funnel plots for the MR analyses of the causal relationship between UBE2C and ccRCC: (C) UBE2C → ccRCC; (D) ccRCC → UBE2C. (E, F) MR leave-one-out sensitivity analyses of the causal relationship between UBE2C and ccRCC: (E) UBE2C → ccRCC; (F) ccRCC → UBE2C.

The IVW method showed that genetically predicted higher serum UBE2C levels were associated with increased ccRCC risk (OR = 1.418, 95% CI [1.085–1.853], *P* = 0.010; Table 2; Fig. 2A). Sensitivity analyses including funnel plot assessment, leave-one-out analysis (Figs. 2C, 2E), Cochran’s Q test (IVW: Q = 22.169, *P* = 0.178), MR-Egger intercept test (*P* = 0.584), and MR-PRESSO global test (*P* = 0.172)—showed no significant heterogeneity or horizontal pleiotropy (Table 3). Conversely, reverse MR analysis provided no evidence that genetic predisposition to ccRCC was associated with altered serum UBE2C levels (IVW: OR = 0.985, 95% CI [0.939–1.032], *P* = 0.519; Table 2; Fig. 2B). Sensitivity analyses further supported the robustness of this null association (Cochran’s Q: *P* = 0.399; MR-Egger: *P* = 0.183; MR-PRESSO: *P* = 0.466; Figs. 2D, 2F; Table 3).

**Table 2:** Two-sample bidirectional MR analysis results.

Exposure	Outcome	SNP	Analytic technique	β	SE	OR (95% CI)	*P*
UBE2C	ccRCC	18	MR Egger	0.146	0.389	1.157 [0.539–2.484]	0.712
		18	Weighted median	0.191	0.180	1.211 [0.849–1.726]	0.288
		18	Inverse variance weighted	0.349	0.136	1.418 [1.085–1.853]	0.010
		18	Simple mode	−0.023	0.346	0.977 [0.495–1.925]	0.947
		18	Weighted mode	0.006	0.335	1.006 [0.521–1.942]	0.984
ccRCC	UBE2C	9	MR Egger	0.026	0.037	1.026 [0.955–1.102]	0.507
		9	Weighted median	0.008	0.033	1.008 [0.944–1.076]	0.815
		9	Inverse variance weighted	−0.016	0.024	0.985 [0.939–1.032]	0.519
		9	Simple mode	0.032	0.058	1.033 [0.922–1.157]	0.590
		9	Weighted mode	0.018	0.040	1.019 [0.942–1.101]	0.654

**Table 3:** MR sensitivity analysis.

Exposure	MR-PRESSO (*P*)	MR-Egger (*P*)	CochranQ (*P*)
UBE2C	0.172	0.584	0.178
ccRCC	0.466	0.183	0.399

### Validation of UBE2C overexpression in clinical ccRCC specimens

**Figure 3:**
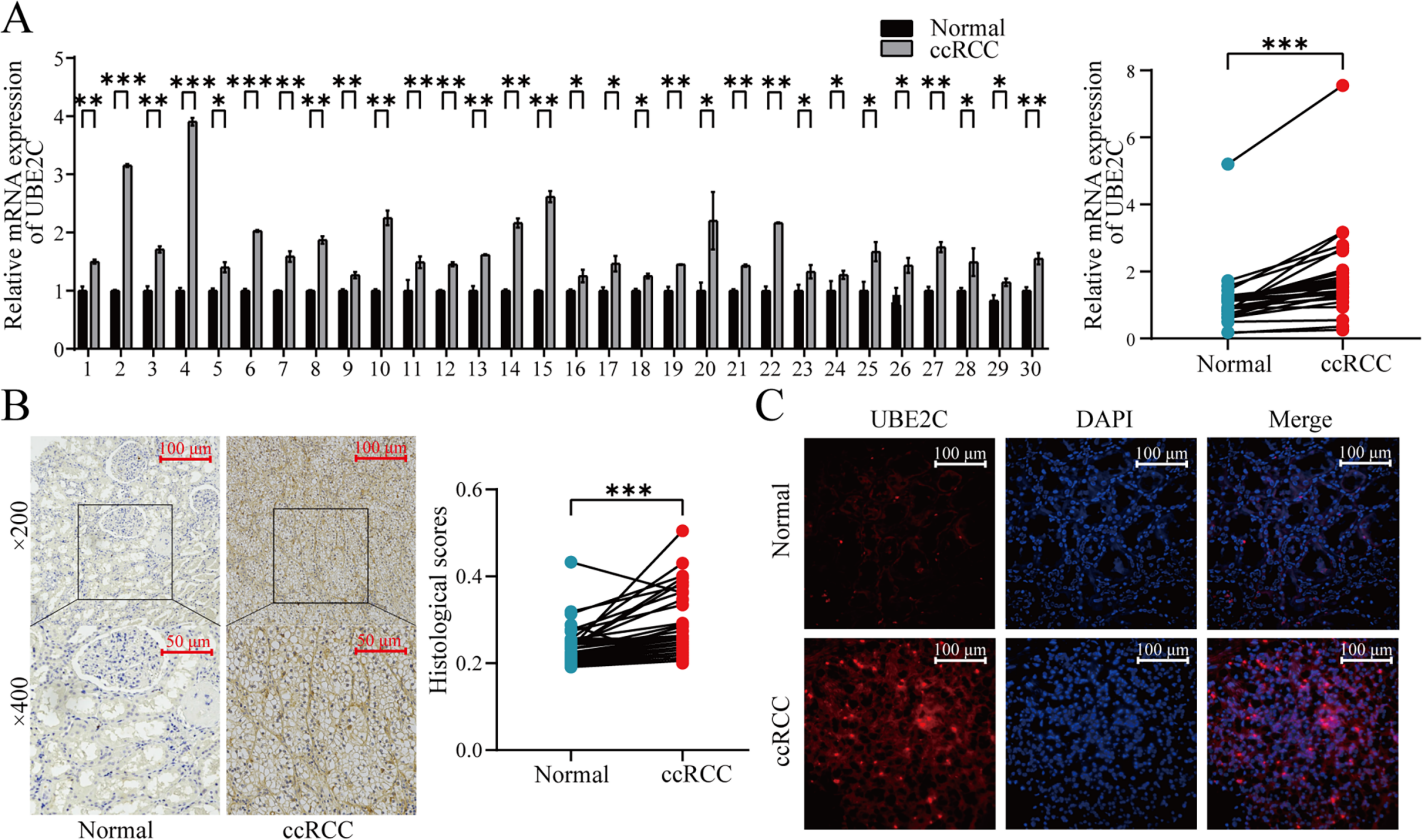
Expression of UBE2C in ccRCC tissues. (A) The expression levels of UBE2C mRNA in 30 pairs of ccRCC tissues and adjacent tissues were detected *via* qRT-PCR. (B) The protein expression of UBE2C in ccRCC tissues and adjacent tissues was detected using IHC assay. (C) The protein distribution and expression levels of UBE2C in ccRCC tissues and adjacent tissues were detected by IF assay. *P < 0.05, **P < 0.01, ***P < 0.001.

qRT-PCR analysis of 30 pairs of ccRCC tissues and adjacent normal tissues showed significantly upregulated UBE2C mRNA expression in tumor tissues (t = 6.235, *P* < 0.001; Fig. 3A). Consistent with this, IHC showed stronger UBE2C protein staining in ccRCC tissues (t = 4.039, *P* < 0.001; Fig. 3B). IF further showed that UBE2C protein overexpression was mainly localized to the cytoplasm and nucleoli of ccRCC cells (Fig. 3C). Based on IHC staining scores, 30 ccRCC patients were divided into high- and low-UBE2C expression groups. High UBE2C expression was significantly correlated with advanced (T stage ${\chi^2}$ = 10.800, *P* = 0.001), lymph node metastasis (N stage, ${\chi^2}$ = 5.714, *P* = 0.017), distant metastasis (M stage, ${\chi^2}$ = 4.658, *P* = 0.031), and higher pathologic stage ( ${\chi^2}$ = 13.393, *P* < 0.001). No significant associations were found with gender ( ${\chi^2}$ = 0.002, *P* = 0.961) or age ( ${\chi^2}$ = 0.136, *P* = 0.713) (Table 4).

**Table 4:** The correlation between UBE2C expression and the clinicopathological characteristics of patients with ccRCC.

Clinical features	Sample size (*n*)	UBE2C expression		*χ* ^*2*^	*P*
		Low (*n* = 15)	High (*n* = 15)		
**Gender *n* (%)**					
Male	13	6 (20%)	7 (23%)	0.002	0.961
Female	17	9 (30%)	8 (27%)		
**Age (year), *n* (%)**					
≤60	14	6 (20%)	8 (27%)	0.136	0.713
>60	16	9 (30%)	7 (23%)		
**T stages, *n* (%)**					
T1–T2	15	12 (40%)	3 (10%)	10.800	0.001
T3–T4	15	3 (10%)	12 (40%)		
**N stages, *n* (%)**					
N0	21	14 (47%)	7 (23%)	5.714	0.017
N1	9	1 (3%)	8 (27%)		
**M stages, *n* (%)**					
M0	23	14 (47%)	9 (30%)	4.658	0.031
M1	7	1 (3%)	6 (20%)		
**Pathological stage, *n* (%)**					
I–II	14	12 (40%)	2 (7%)	13.393	<0.001
III–IV	16	3 (10%)	13 (43%)		

### UBE2C expression and functional characterization in ccRCC cell lines

**Figure 4:**
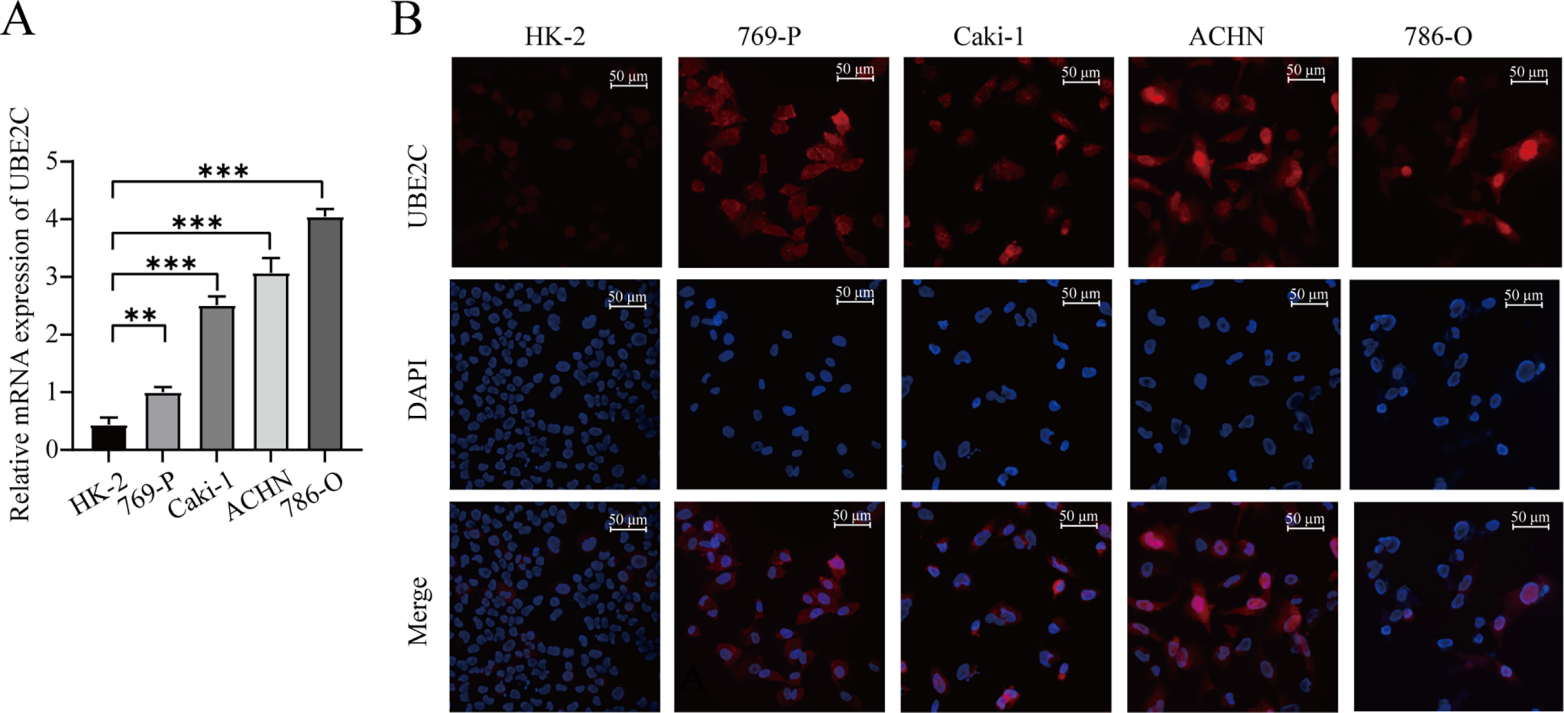
The expression of UBE2C in ccRCC cell lines. (A) The expression levels of UBE2C mRNA in the normal renal cell line HK-2 and four ccRCC cell lines, namely 769-P, Caki-1, 786-O, and ACHN, were measured *via* qRT-PCR. (B) The protein distribution and expression levels of UBE2C in the normal renal cell line HK-2 and the four ccRCC cell lines 769-P, Caki-1, 786-O, and ACHN were determined using IF assay. **P < 0.01, ***P < 0.001.

qRT-PCR analyses confirmed that UBE2C mRNA expression was significantly upregulated in all four ccRCC cell lines (Caki-1, 786-O, 769-P, ACHN) compared to the immortalized human renal tubular epithelial cell line HK-2 (*P* < 0.01; Fig. 4A). IF staining consistently showed enhanced UBE2C protein expression in the cytoplasm and nucleoli of cancer cells (Fig. 4B). Due to their relatively high UBE2C expression levels, ACHN and 786-O cells were selected for knockdown experiments, while 769-P cells were chosen for overexpression studies.

### Establishment of stable UBE2C-knockdown and overexpressing cell lines

**Figure 5:**
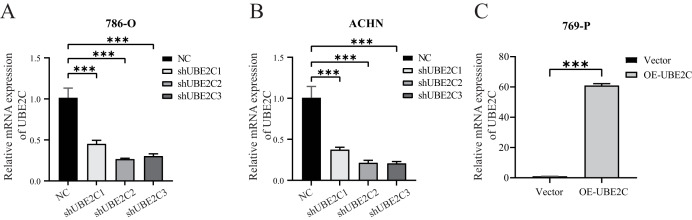
Establishment of ccRCC cell lines with stable downregulation and overexpression of UBE2C. (A, B) The expression levels of UBE2C mRNA in 786-O and ACHN cells following infection with interfering lentiviruses were detected using qRT-PCR. (C) The expression levels of UBE2C mRNA in 769-P cells after infection with overexpressing lentiviruses were determined by qRT-PCR. ***P < 0.001.

Three shRNA sequences targeting UBE2C and a NC were used to generate stable knockdown lines in 786-O and ACHN cells. qRT-PCR confirmed strong knockdown efficiency for all three shRNAs (*P* < 0.001; Figs. 5A, 5B). Among these, shUBE2C#1 and shUBE2C#2 were selected for subsequent experiments. shUBE2C#2 demonstrated the strongest knockdown efficiency, whereas shUBE2C#1 exhibited more stable and reproducible suppression of UBE2C expression compared with shUBE2C#3. For overexpression, a lentiviral construct harboring UBE2C was transduced into 769-P cells, leading to a substantial increase in UBE2C mRNA levels compared to the vector control (*P* < 0.001; Fig. 5C).

### UBE2C promotes malignant phenotypes of ccRCC cells *in vitro*

**Figure 6:**
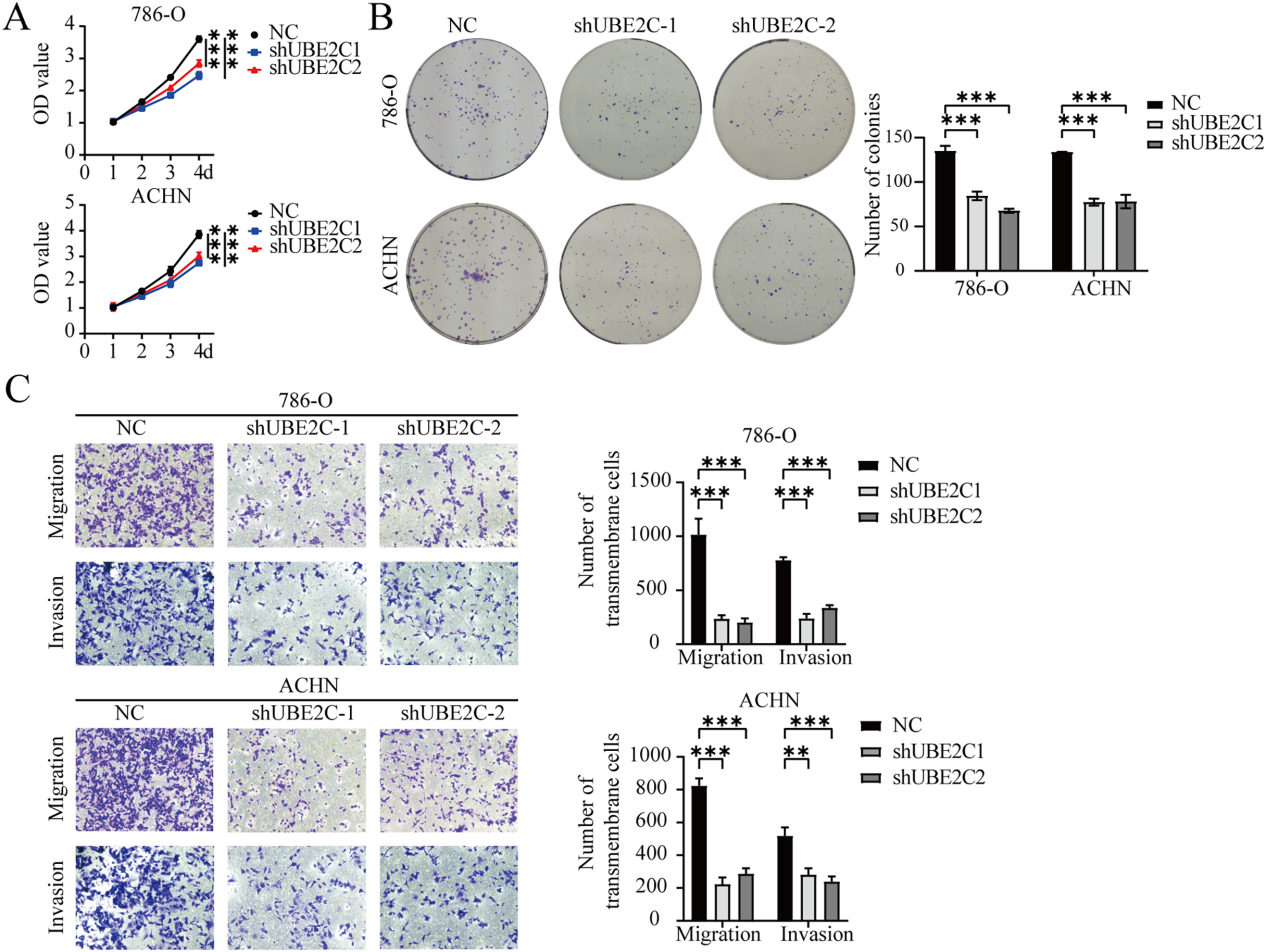
The impact of UBE2C knockdown on the malignant behaviors of ccRCC cells. (A) The results of the CCK-8 assay demonstrated that the proliferative capacity of 786-O and ACHN cells was diminished following UBE2C knockdown. (B) The outcomes of the colony formation assay revealed that the colony-forming ability of 786-O and ACHN cells declined after UBE2C knockdown, accompanied by reduced proliferation. (C) The Transwell assay was utilized to evaluate the migratory and invasive capabilities of ccRCC cells. The knockdown of UBE2C expression effectively inhibited the migration and invasion abilities of 786-O and ACHN cells. **P < 0.01, ***P < 0.001.

CCK-8 assays showed that UBE2C knockdown significantly inhibited the proliferation of 786-O and ACHN cells (*P* < 0.001; Fig. 6A). Colony formation assays further confirmed that UBE2C depletion markedly reduced the clonogenic survival of both cell lines (*P* < 0.001; Fig. 6B). Transwell assays (without Matrigel for migration, with Matrigel for invasion) showed that UBE2C knockdown significantly impaired the migratory and invasive capacities of 786-O (*P* < 0.001) and ACHN cells (*P* < 0.01; Fig. 6C).

### Overexpression of UBE2C enhances proliferation, migration, and invasion

**Figure 7:**
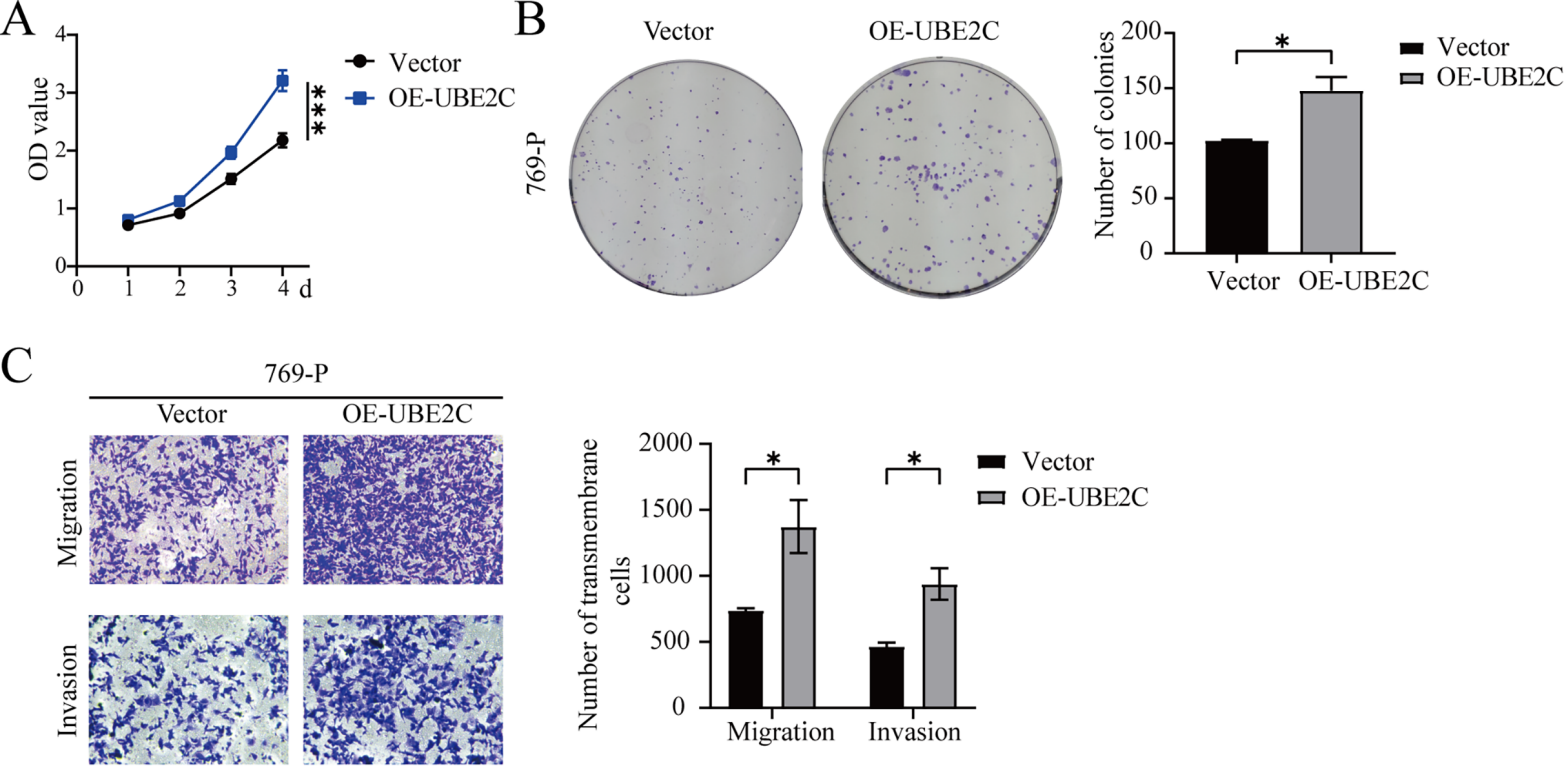
The impact of UBE2C overexpression on the malignant behaviors of ccRCC cells. (A) The CCK-8 assay was employed to evaluate the effect of UBE2C overexpression on the proliferation of 769-P cells. (B) The colony formation assay was conducted to detect the influence of UBE2C upregulation on the proliferative capacity of 769-P cells. (C) The impact of UBE2C upregulation on the migratory and invasive capabilities of 769-P cells was investigated. *P < 0.05, ***P < 0.001.

Conversely, UBE2C overexpression in 769-P cells significantly enhanced cell proliferation (*P* < 0.001; Fig. 7A), promoted colony formation (*P* < 0.05; Fig. 7B), and increased migratory and invasive abilities (*P* < 0.05; Fig. 7C).

### UBE2C knockdown inhibits tumor growth *in vivo*

**Figure 8:**
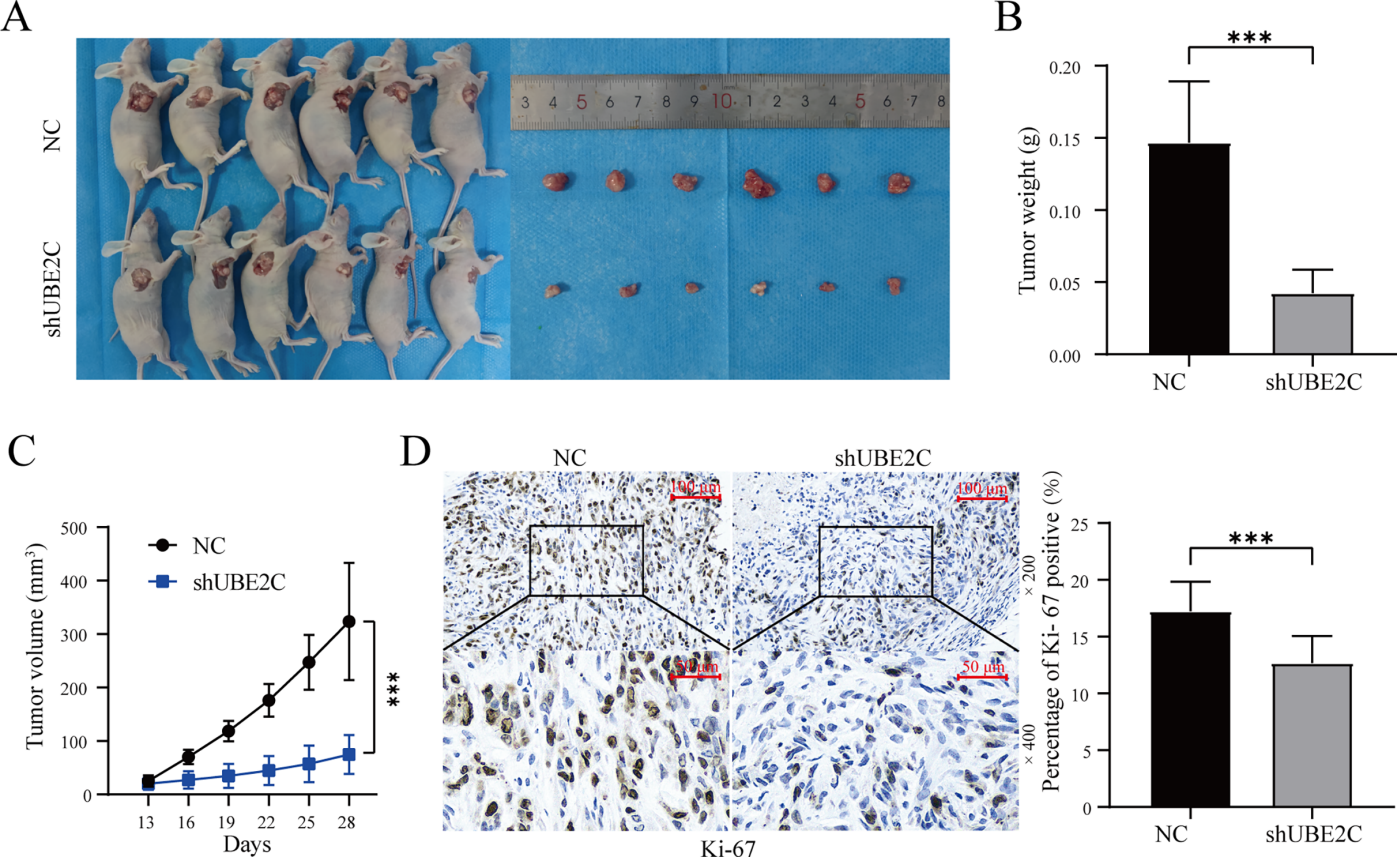
The influence of UBE2C knockdown on the subcutaneous tumor-forming ability of ccRCC cells in nude mice. (A) Photographs of subcutaneous transplants tumors in nude mice. (B) Weights of transplants tumors in the UBE2C-knockdown group and the control group. (C) Growth curves of transplants tumors. (D) Expression of Ki-67 detected by IHC in transplant tumors and statistical analysis of the positive rate. ***P < 0.001.

ACHN cells stably expressing shUBE2C or NC were subcutaneously injected into nude mice (*n* = 6 per group). Tumors formed successfully in both groups (Fig. 8A). The shUBE2C group had significantly lower tumor weight (*P* < 0.001; Fig. 8B) and slower tumor growth (*P* < 0.001; Fig. 8C) compared to the control group. IHC analysis of xenograft tumors showed a significant reduction in Ki-67 positive cells in the shUBE2C group (*P* < 0.001; Fig. 8D), indicating decreased proliferative activity.

### Mechanistic insights into UBE2C function in ccRCC

**Figure 9:**
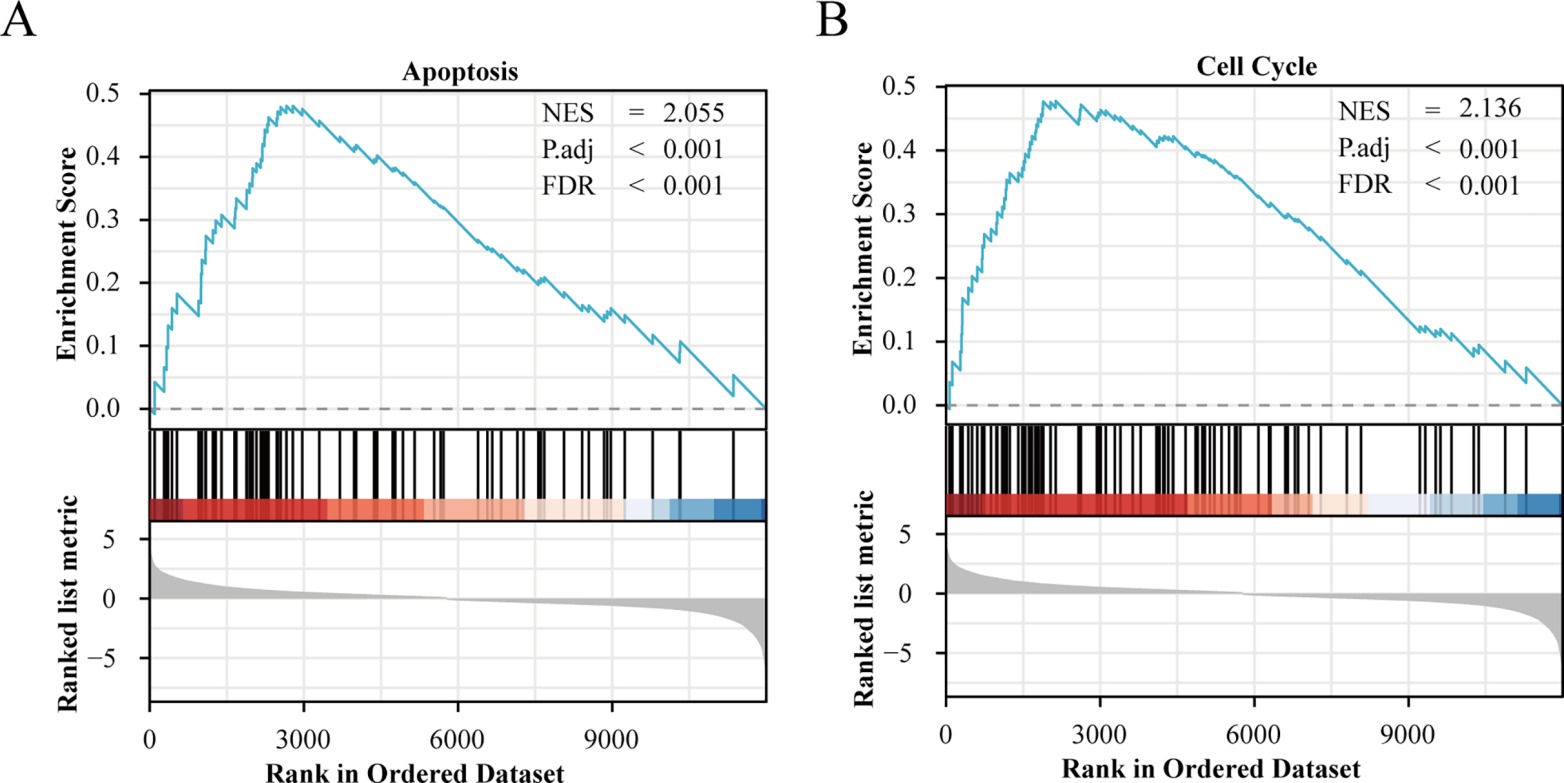
GSEA within the framework of the GEO database. (A) The correlation between UBE2C and the apoptosis signaling pathway. (B) The correlation between UBE2C and the cell cycle signaling pathway.

**Figure 10:**
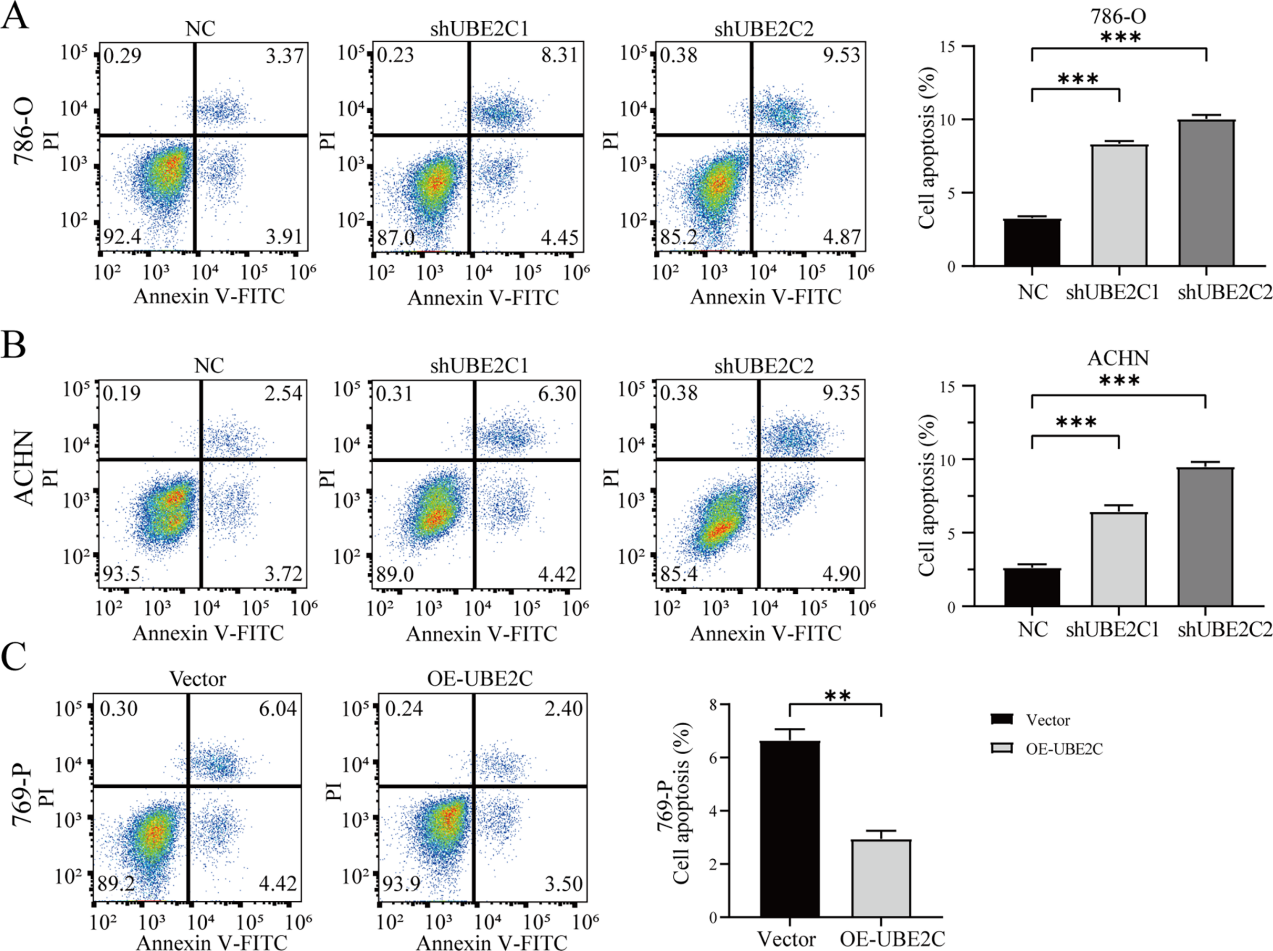
Flow cytometry was employed to assess cell apoptosis. (A) The impact of UBE2C knockdown on the apoptosis of 786-O cells. (B) The influence of UBE2C knockdown on the apoptosis of ACHN cells. ***P < 0.001. (C) Regarding the effect of UBE2C upregulation on the apoptosis of 769-P cells, **P < 0.01.

**Figure 11:**
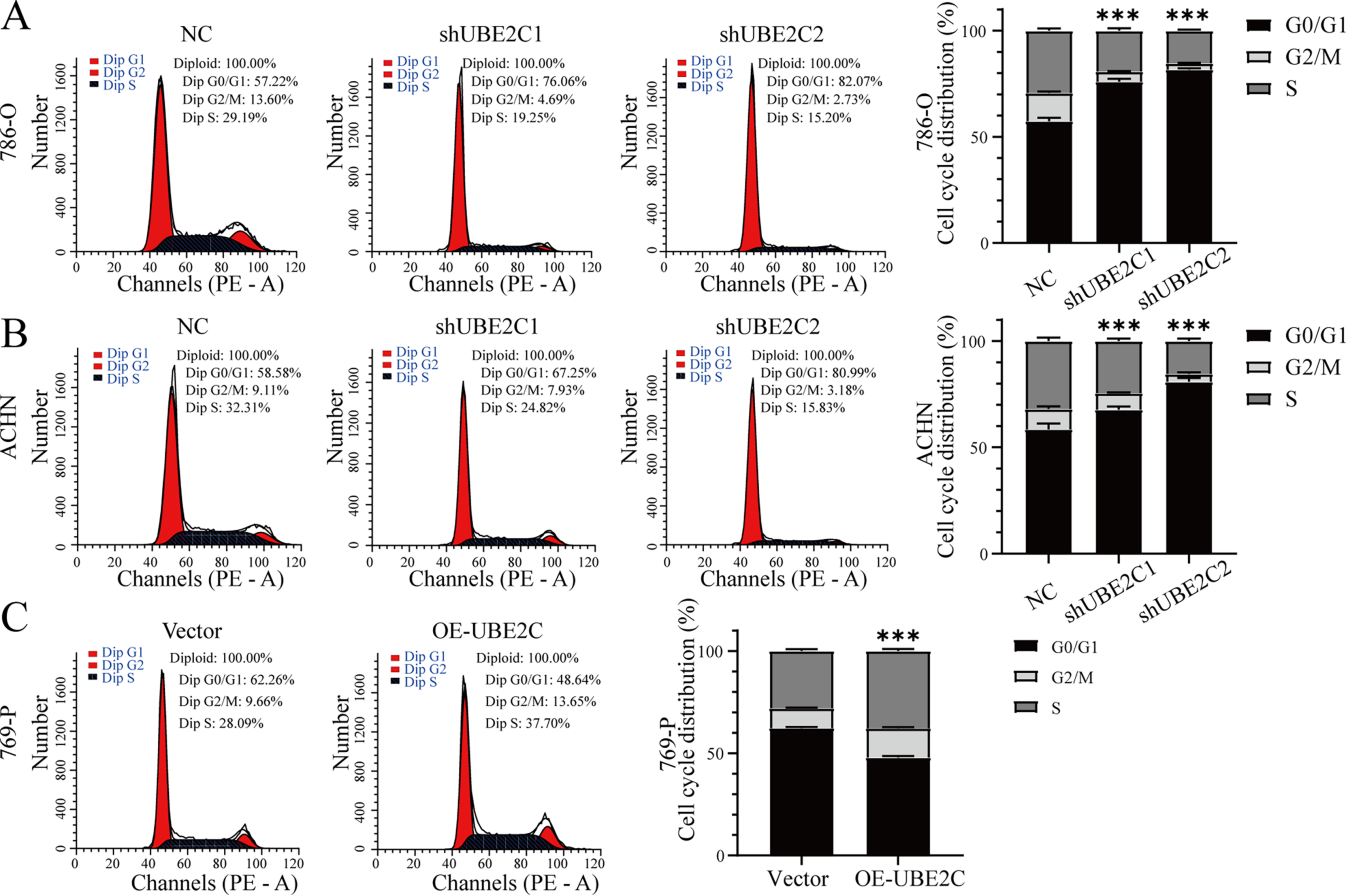
Cell cycle analysis *via* flow cytometry. (A) The impact of UBE2C knockdown on the cell cycle of 786-O cells. (B) The influence of UBE2C knockdown on the cell cycle of ACHN cells. ***P < 0.001. (C) The effect of UBE2C upregulation on the cell cycle of 769-P cells. ***P < 0.001.

GSEA of the GEO dataset GSE53757 showed that high UBE2C expression was significantly enriched in gene sets related to apoptosis (NES = 2.005, *P* < 0.001) and cell cycle progression (NES = 2.136, *P* < 0.001) (Figs. 9A, 9B). Flow cytometric analysis using Annexin V/PI staining showed that UBE2C knockdown significantly induced apoptosis in 786-O and ACHN cells (*P* < 0.001; Figs. 10A, 10B), cell cycle analysis further showed that UBE2C knockdown in 786-O and ACHN cells resulted in significant accumulation of cells in the G0/G1 phase and a corresponding decrease in the S and G2/M phases (*P* < 0.001; Figs. 11A, 11B). Conversely, UBE2C overexpression in 769-P cells reduced the G0/G1 population and increased the proportion of cells in the S and G2/M phases (*P* < 0.001; Fig. 11C), indicating that UBE2C promotes G1/S phase transition and cell cycle progression.

### UBE2C rescue reverses malignant phenotypes and regulates cell cycle and apoptosis

**Figure 12:**
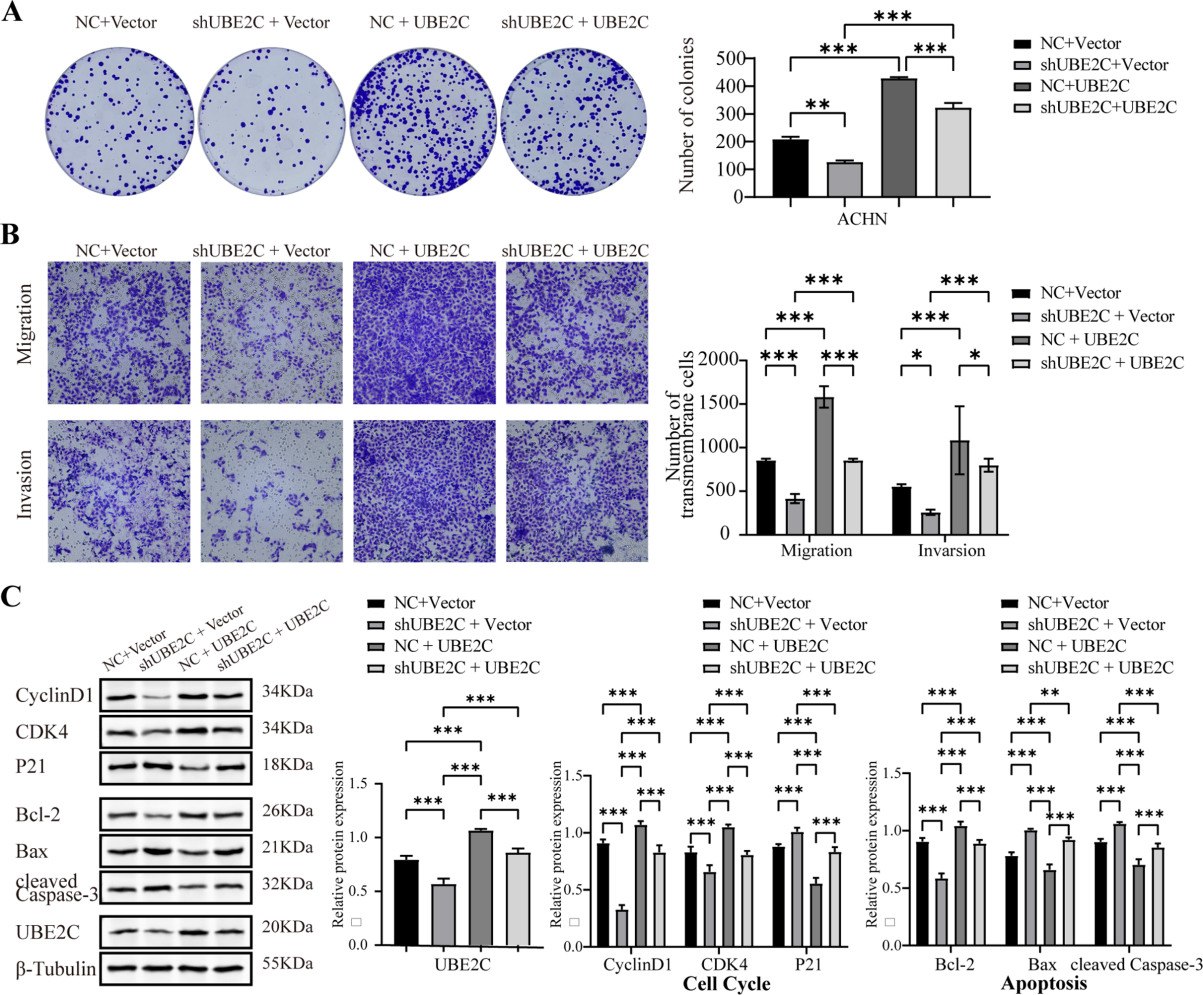
UBE2C rescue reverses malignant phenotypes and regulates cell cycle and apoptosis in ccRCC cells. (A) Colony formation assays showing that UBE2C knockdown significantly suppressed the clonogenic ability of ACHN cells, whereas re-expression of UBE2C restored colony formation. (B) Transwell migration and invasion assays demonstrating that silencing UBE2C inhibited the migratory and invasive capacities of ACHN cells, while UBE2C re-expression significantly reversed these inhibitory effects. (C) Western blot analysis of cell cycle– and apoptosis-related proteins in the indicated groups. UBE2C knockdown decreased Cyclin D1 and CDK4 expression and increased p21 levels, indicating inhibition of cell cycle progression. In parallel, UBE2C silencing reduced Bcl-2 expression and increased Bax and cleaved-caspase-3 levels, suggesting enhanced apoptosis. Re-expression of UBE2C reversed these molecular changes. Quantitative analysis of protein expression is shown on the right. Data are presented as mean ± SD from three independent experiments. *P < 0.05, **P < 0.01, ***P < 0.001.

Rescue experiments were performed to further validate the functional role of UBE2C. UBE2C knockdown markedly suppressed colony formation, migration, and invasion of ACHN cells, whereas re-expression of UBE2C significantly restored these malignant phenotypes (*P* < 0.05; Figs. 12A, 12B). Mechanistically, western blot analysis showed that UBE2C silencing downregulated Cyclin D1 and CDK4 and upregulated p21, indicating inhibition of cell cycle progression. Meanwhile, UBE2C knockdown decreased Bcl-2 expression and increased Bax and cleaved-caspase-3 levels, suggesting activation of apoptosis. Notably, re-expression of UBE2C reversed these changes, restoring both cell cycle progression and anti-apoptotic signaling (*P* < 0.05; Fig. 12C). These results demonstrate that UBE2C promotes ccRCC malignant behavior by regulating cell cycle progression and apoptosis.

## Discussion

ccRCC is a life-threatening urological malignancy characterized by substantial molecular heterogeneity and limited treatment options for advanced disease (Wang et al., 2025a). In the present study, an integrated multi-omics strategy combining bioinformatics analysis, MR, and functional validation was employed to investigate the role of UBE2C in ccRCC. The results demonstrated that UBE2C was significantly overexpressed in ccRCC and was associated with enhanced cell cycle progression and reduced apoptosis. Furthermore, MR analysis suggested a genetic association between serum UBE2C levels and ccRCC risk, indicating that UBE2C may be involved in ccRCC development. Collectively, these findings suggest that UBE2C may serve as a potential biomarker and therapeutic target in ccRCC (Lv et al., 2023).

The UPS plays a central role in maintaining protein homeostasis and regulating key cellular processes, including cell division and programmed cell death (Kandel, Jung & Neal, 2024; Tang, Lai & Pervaiz, 2024). Dysregulation of UPS components has been widely reported in multiple cancers, including ccRCC (Wang et al., 2023b). Among these components, UBE2C is an E2 ubiquitin-conjugating enzyme involved in the ubiquitination of mitotic regulators such as cyclin B1 and securin, thereby contributing to mitotic progression and genomic stability (Wang et al., 2025b; Zhang et al., 2023). Consistent with observations in other malignancies, including breast, lung, and bladder cancers, UBE2C was significantly upregulated in ccRCC tissues and cell lines. Elevated UBE2C expression was associated with advanced tumor stage, lymph node and distant metastasis, higher histological grade, and poorer overall survival. These findings are consistent with previous reports linking UBE2C to aggressive clinicopathological features across multiple tumor types (Xu et al., 2025).

Receiver operating characteristic analysis demonstrated that UBE2C distinguished tumor from normal tissues with a high diagnostic accuracy. Multivariate Cox regression analysis further identified UBE2C expression as an independent prognostic factor. These findings are consistent with previous studies in endometrial cancer and glioblastoma, where elevated UBE2C expression was associated with unfavorable clinical outcomes (Domentean et al., 2023; Jalali et al., 2024; Zhao et al., 2023). Together, these observations suggest that UBE2C may function as an important regulator of tumor progression, possibly through its involvement in cell cycle control.

An additional strength of this study is the incorporation of bidirectional Mendelian randomization analysis. The results indicated that genetically predicted elevated serum UBE2C levels were associated with an increased risk of ccRCC, whereas reverse MR analysis did not support an effect of ccRCC on UBE2C levels. These findings suggest a potential genetic association between UBE2C and ccRCC risk (Zhu et al., 2022). However, MR analysis evaluates genetic associations rather than direct mechanistic causality. Therefore, the present findings provide complementary genetic evidence supporting a potential role of UBE2C in ccRCC, alongside bioinformatics and experimental observations.

Functional experiments further demonstrated that UBE2C was associated with proliferative and invasive behaviors in ccRCC cells. Knockdown of UBE2C significantly reduced cell proliferation, clonogenic ability, migration, and invasion, whereas UBE2C overexpression enhanced these malignant phenotypes (Cai et al., 2024; Lu et al., 2021). Xenograft experiments further showed that UBE2C depletion suppressed tumor growth *in vivo* and was accompanied by a reduced Ki-67 index, supporting a role for UBE2C in ccRCC progression.

Gene set enrichment analysis revealed significant enrichment of cell cycle and apoptosis-related pathways in tumors with high UBE2C expression. Flow cytometry analysis further demonstrated that UBE2C knockdown induced G0/G1 phase arrest and increased apoptosis, whereas UBE2C overexpression promoted cell cycle progression. These observations are consistent with the known involvement of UBE2C in cell cycle regulation (Bodrug et al., 2023).

Importantly, rescue experiments further supported the functional role of UBE2C in ccRCC. Re-expression of UBE2C in knockdown cells restored proliferation, migration, and invasion abilities, indicating that the observed phenotypic changes were mediated by UBE2C. These findings strengthen the functional link between UBE2C expression and malignant behaviors in ccRCC cells.

Western blot analysis provided additional mechanistic insights. UBE2C knockdown decreased Cyclin D1 and CDK4 expression while increasing P21 levels, suggesting inhibition of cell cycle progression. In addition, Bcl-2 expression was reduced, whereas Bax and cleaved-caspase-3 levels were increased, indicating activation of apoptosis. Re-expression of UBE2C reversed these alterations. Together, these findings suggest that UBE2C may contribute to ccRCC progression, at least in part, through regulation of cell cycle progression and apoptosis.

Although Mendelian randomization analysis suggested a genetic association between serum UBE2C levels and ccRCC risk, this does not establish direct mechanistic causality. Furthermore, the relationship between circulating UBE2C and tumor-intrinsic UBE2C activity remains unclear. The cellular origin of serum UBE2C, such as tumor cells or infiltrating immune cells, has not been determined. Therefore, circulating UBE2C levels may not directly reflect intratumoral ubiquitination activity. These considerations highlight the need for further mechanistic studies linking systemic and tumor-specific UBE2C expression.

Despite the significant findings, this study has several limitations. First, while the validation cohort confirmed UBE2C differential expression, the relatively small sample size limits robust subgroup analyses. Larger, multi-center validation studies are required to fully establish the prognostic and diagnostic value of UBE2C across diverse ccRCC patient populations (Lv et al., 2023). Second, although MR analysis provides genetic evidence for an association between serum UBE2C levels and ccRCC risk, the precise molecular mechanisms connecting circulating UBE2C to tumorigenesis remain unclear (Xu et al., 2025). Third, while functional studies linked UBE2C knockdown to dysregulation of apoptosis and cell cycle progression, the specific protein substrates directly ubiquitinated by UBE2C in ccRCC remain unknown. As an E2 conjugating enzyme, UBE2C functions in cooperation with specific E3 ubiquitin ligases, particularly the anaphase-promoting complex/cyclosome (APC/C) (Zhang et al., 2023). Identifying the relevant UBE2C-E3 ligase complexes and their downstream substrates will be critical for understanding the precise pathogenic mechanisms (Yang et al., 2025).

Future studies should focus on identifying UBE2C-specific ubiquitination substrates in ccRCC. Proteomic approaches such as ubiquitin remnant profiling combined with UBE2C perturbation may help define global ubiquitination dynamics. In addition, given the central role of hypoxia signaling in ccRCC, investigating potential interactions between UBE2C and HIF-related pathways may provide further mechanistic insights (Lu et al., 2021; Zhang et al., 2024). Another important direction is exploring the relationship between UBE2C and the immune microenvironment, as the ubiquitin-proteasome system regulates immune checkpoint molecules (Golijanin et al., 2023; Salgia, Dizman & Pal, 2025).

From a therapeutic perspective, UBE2C represents a potential target for ccRCC. Although directly targeting E2 enzymes remains challenging, alternative strategies such as disrupting UBE2C-E3 interactions or developing UBE2C-directed protein degradation approaches may provide specificity (Ma et al., 2022). Furthermore, UBE2C expression may serve as a biomarker for patient stratification. Monitoring circulating UBE2C levels may also provide a non-invasive indicator for disease progression or therapeutic response, although this requires further validation.

## Conclusions

UBE2C was significantly upregulated in ccRCC and was associated with aggressive clinicopathological features and poor prognosis. Functional and rescue experiments suggested that UBE2C may promote proliferation, migration, and invasion while inhibiting apoptosis. These findings indicate that UBE2C may contribute to ccRCC progression through regulation of cell cycle and apoptotic pathways. Nevertheless, further studies are required to identify direct ubiquitination substrates and clarify the underlying molecular mechanisms.

## Supplemental Information

10.7717/peerj.21436/supp-1 Supplemental Information 1 Original Images for Microscopy (Part 1).

10.7717/peerj.21436/supp-2 Supplemental Information 2 Original Images for Microscopy (Part 2).

10.7717/peerj.21436/supp-3 Supplemental Information 3 Original Images for Microscopy (Part 3).

10.7717/peerj.21436/supp-4 Supplemental Information 4 Original Images for Microscopy (Part 4).

10.7717/peerj.21436/supp-5 Supplemental Information 5 Original Images for Microscopy (Part 5).

10.7717/peerj.21436/supp-6 Supplemental Information 6 Original Images for Microscopy (Part 6).

10.7717/peerj.21436/supp-7 Supplemental Information 7 Photographs of the tumors 1.

10.7717/peerj.21436/supp-8 Supplemental Information 8 Photographs of the tumors 2.

10.7717/peerj.21436/supp-9 Supplemental Information 9 Photographs of the tumors 3.

10.7717/peerj.21436/supp-10 Supplemental Information 10 ARRIVE checklist.

10.7717/peerj.21436/supp-11 Supplemental Information 11 MIQE checklist.

10.7717/peerj.21436/supp-12 Supplemental Information 12 MR-PRESSO (ccRCC to UBE2C, English revision).

10.7717/peerj.21436/supp-13 Supplemental Information 13 STROBE checklist.

10.7717/peerj.21436/supp-14 Supplemental Information 14 Raw data.

10.7717/peerj.21436/supp-15 Supplemental Information 15 Flow cytometry data.

10.7717/peerj.21436/supp-16 Supplemental Information 16 Tumor data.

10.7717/peerj.21436/supp-17 Supplemental Information 17 Code.

10.7717/peerj.21436/supp-18 Supplemental Information 18 Primer sequences used for qRT-PCR.

10.7717/peerj.21436/supp-19 Supplemental Information 19 Antibodies used for Western blot analysis.

10.7717/peerj.21436/supp-20 Supplemental Information 20 Figure 12 A Colony Formation Assay data.

10.7717/peerj.21436/supp-21 Supplemental Information 21 Figure 12 B Transwell data.

10.7717/peerj.21436/supp-22 Supplemental Information 22 Figure 12 C Western Blot data.

## Additional Information and Declarations

### Data Availability

The following information was supplied regarding data availability:

The data is available at FigShare:

- Qin, Tianzi (2025). UBE2C. figshare. Journal contribution. https://doi.org/10.6084/m9.figshare.30685424.v2

The flow cytometry data is available at FigShare:

- Qin, Tianzi (2026). UBE2C Flow Cytometry Data. figshare. Dataset. https://doi.org/10.6084/m9.figshare.31220803.v1

The data is available at NCBI: GSE53757

RNA-seq data were retrieved from The Cancer Genome Atlas (TCGA), TCGA accession/project ID: TCGA-KIRC:

- https://portal.gdc.cancer.gov/projects/TCGA-KIRC.
