# Supplementary figures and images for: UBE2C contributes to malignant phenotypes in clear cell renal cell carcinoma via cell cycle and apoptosis regulation
Source: PeerJ. 2026 Jun 18;14:e21436. doi: 10.7717/peerj.21436 (PMC13283363; doi:10.7717/peerj.21436)

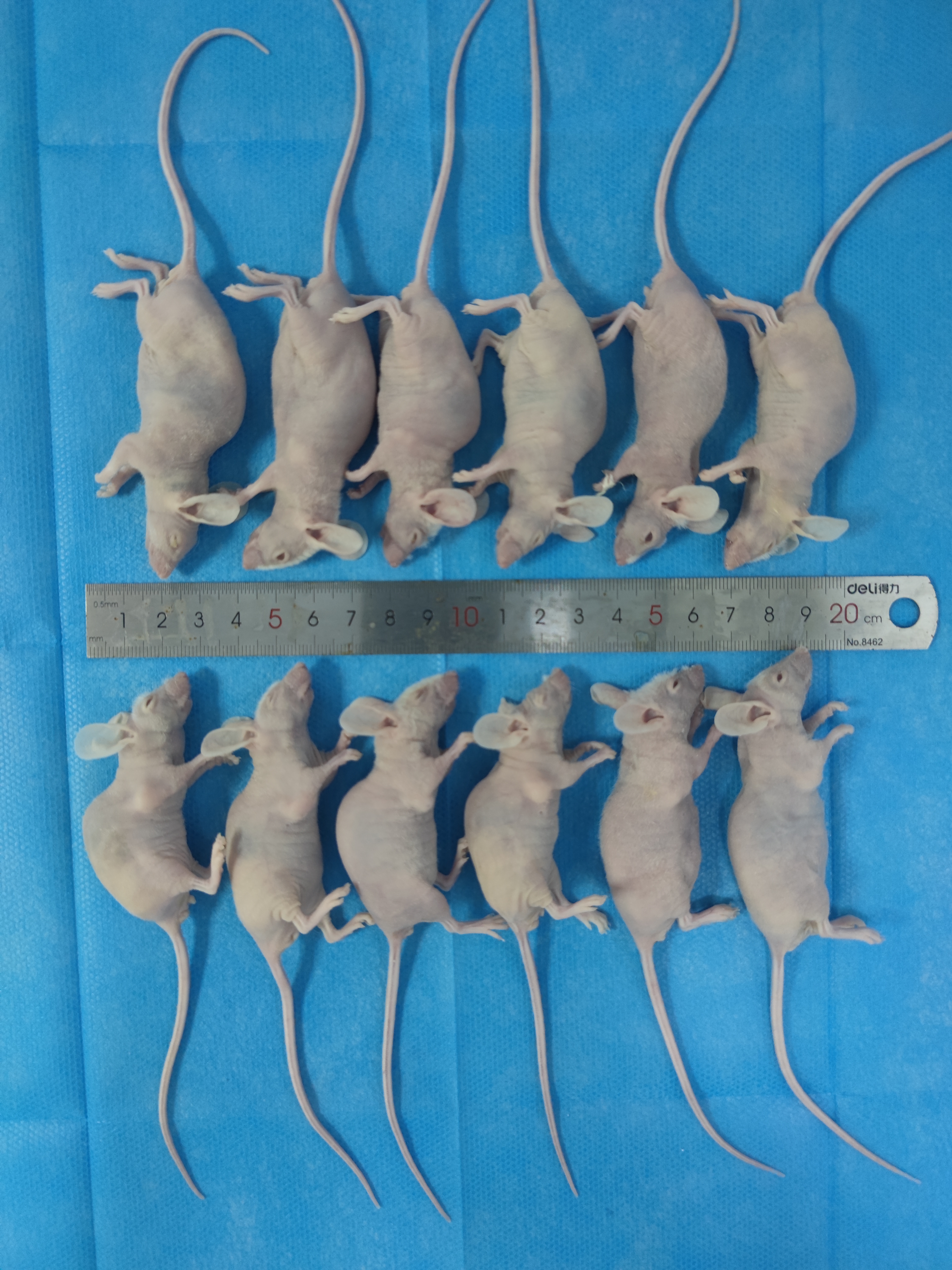

Supplement: Supplemental Information 7 [file peerj-14-21436-s007.jpg]

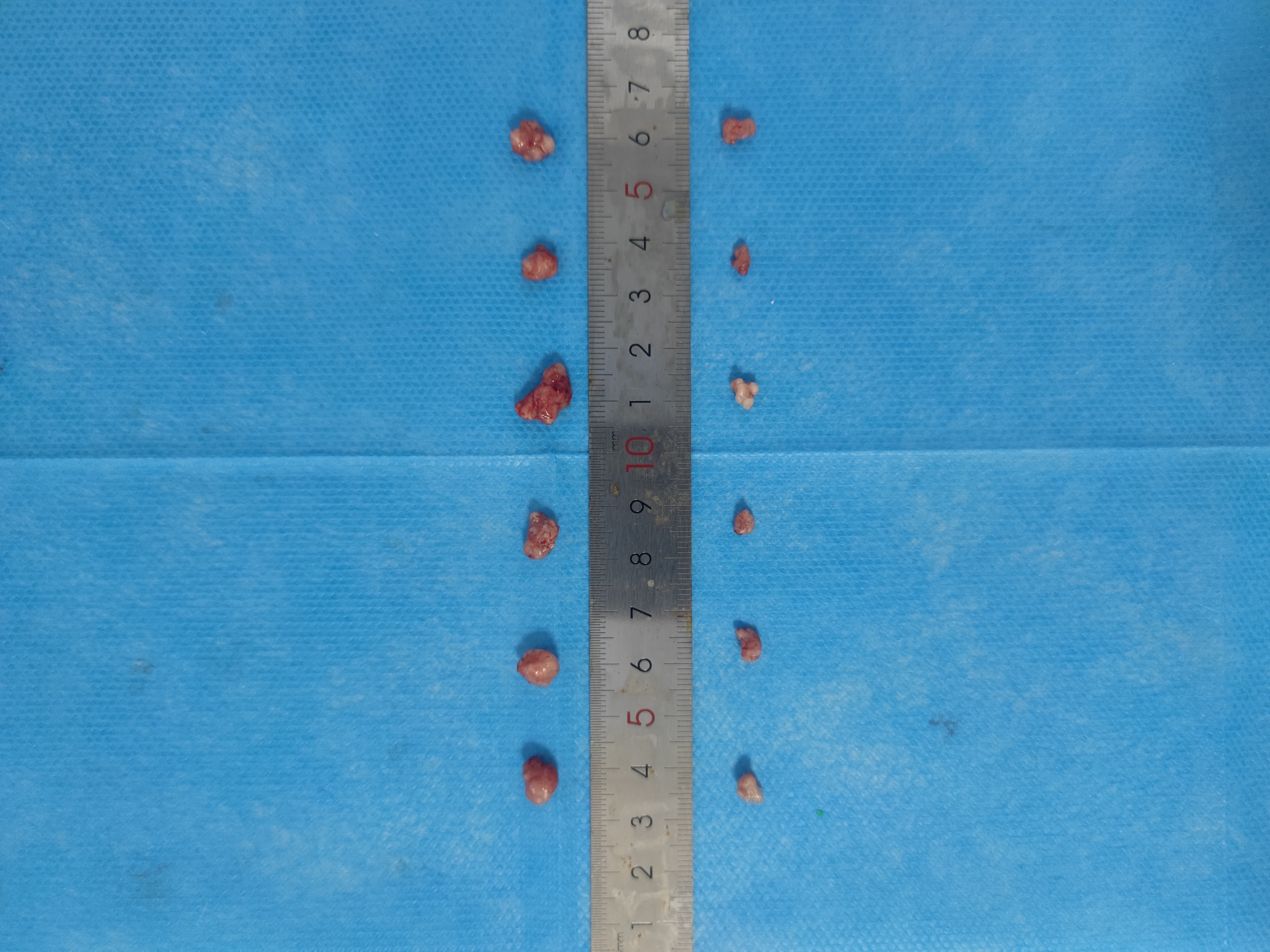

Supplement: Supplemental Information 8 [file peerj-14-21436-s008.jpg]

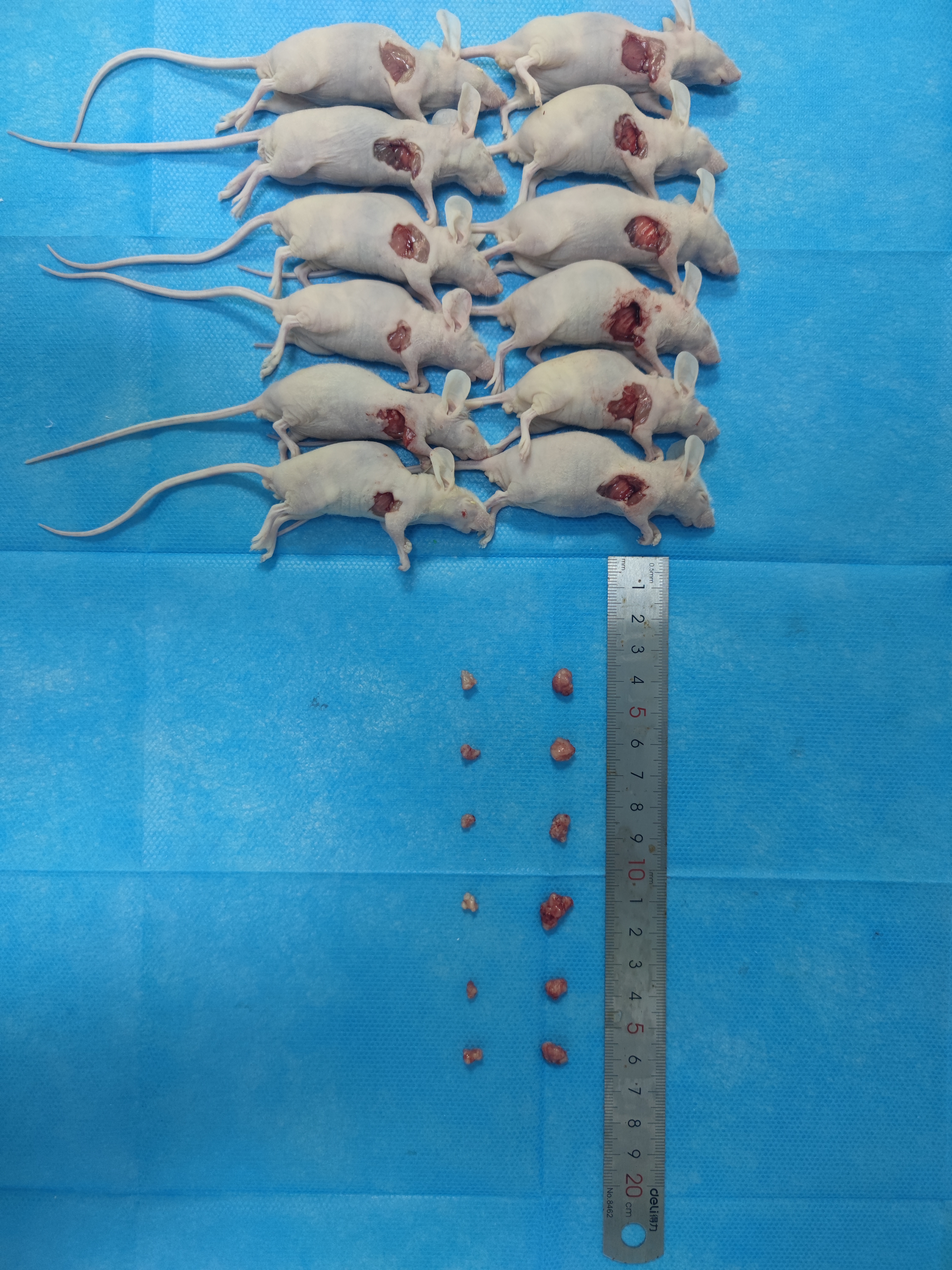

Supplement: Supplemental Information 9 [file peerj-14-21436-s009.jpg]

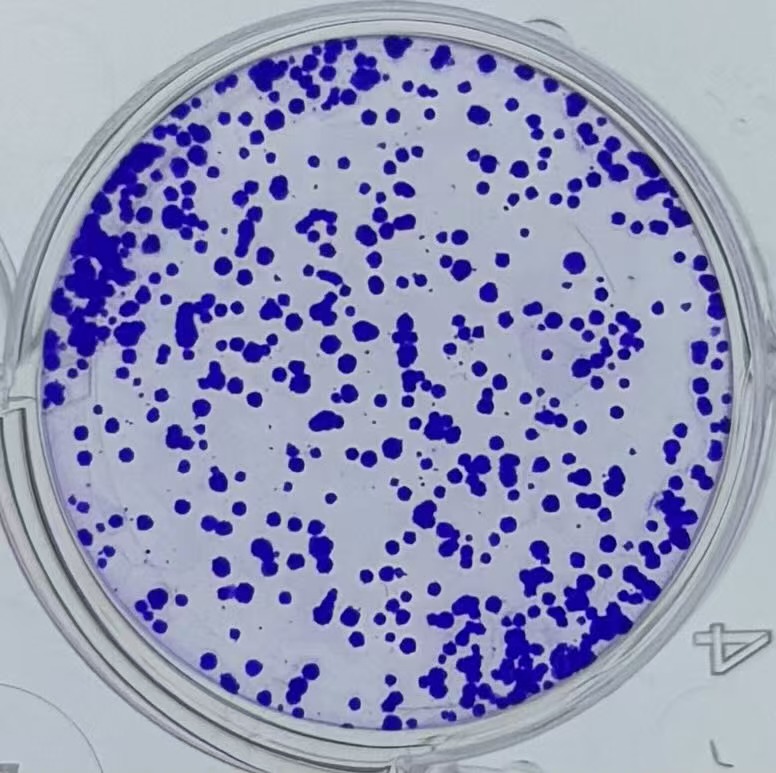

Supplement: Supplemental Information 20 [file peerj-14-21436-s020.zip › NC+UBE2C.jpg]

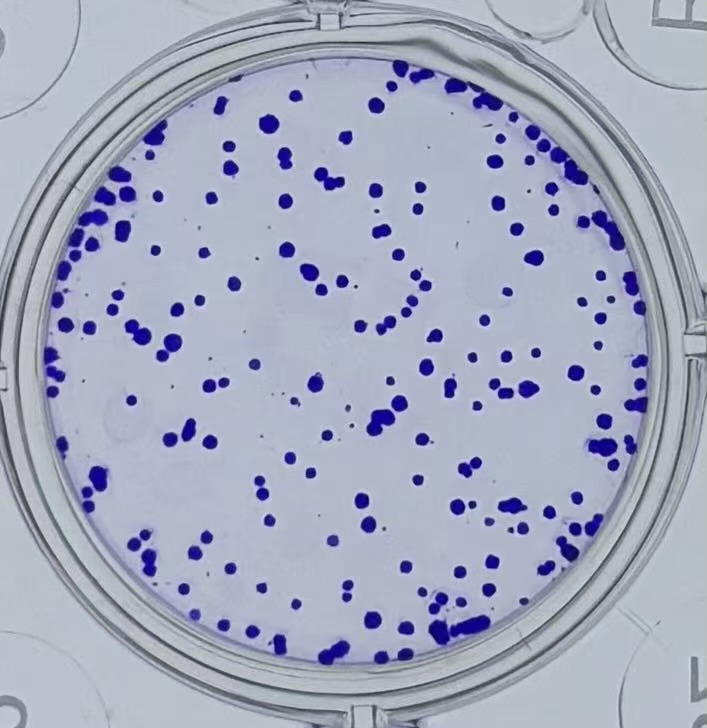

Supplement: Supplemental Information 20 [file peerj-14-21436-s020.zip › NC+Vector.jpg]

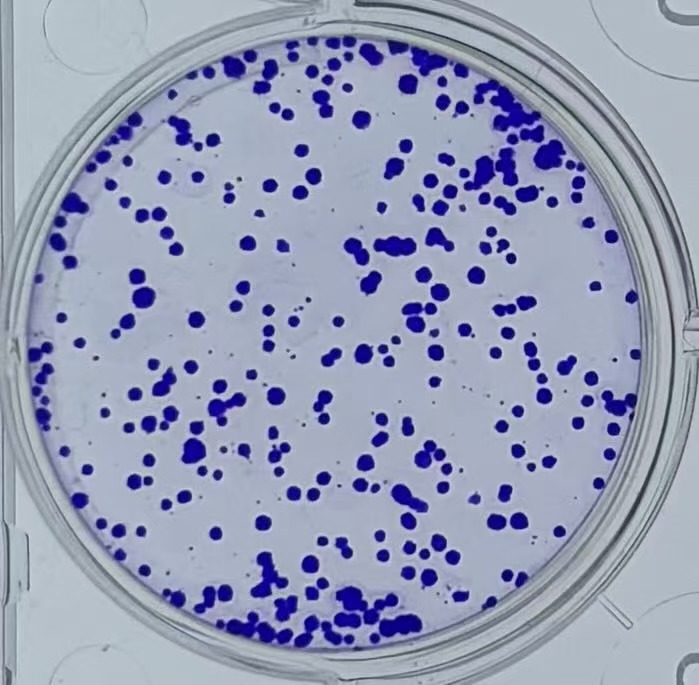

Supplement: Supplemental Information 20 [file peerj-14-21436-s020.zip › shUBE2C+UBE2C.jpg]

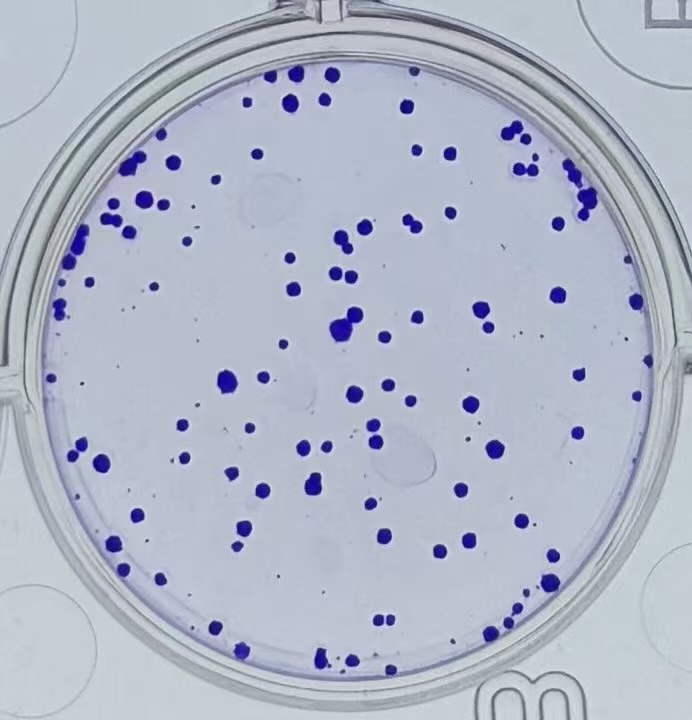

Supplement: Supplemental Information 20 [file peerj-14-21436-s020.zip › shUBE2C+Vector.jpg]

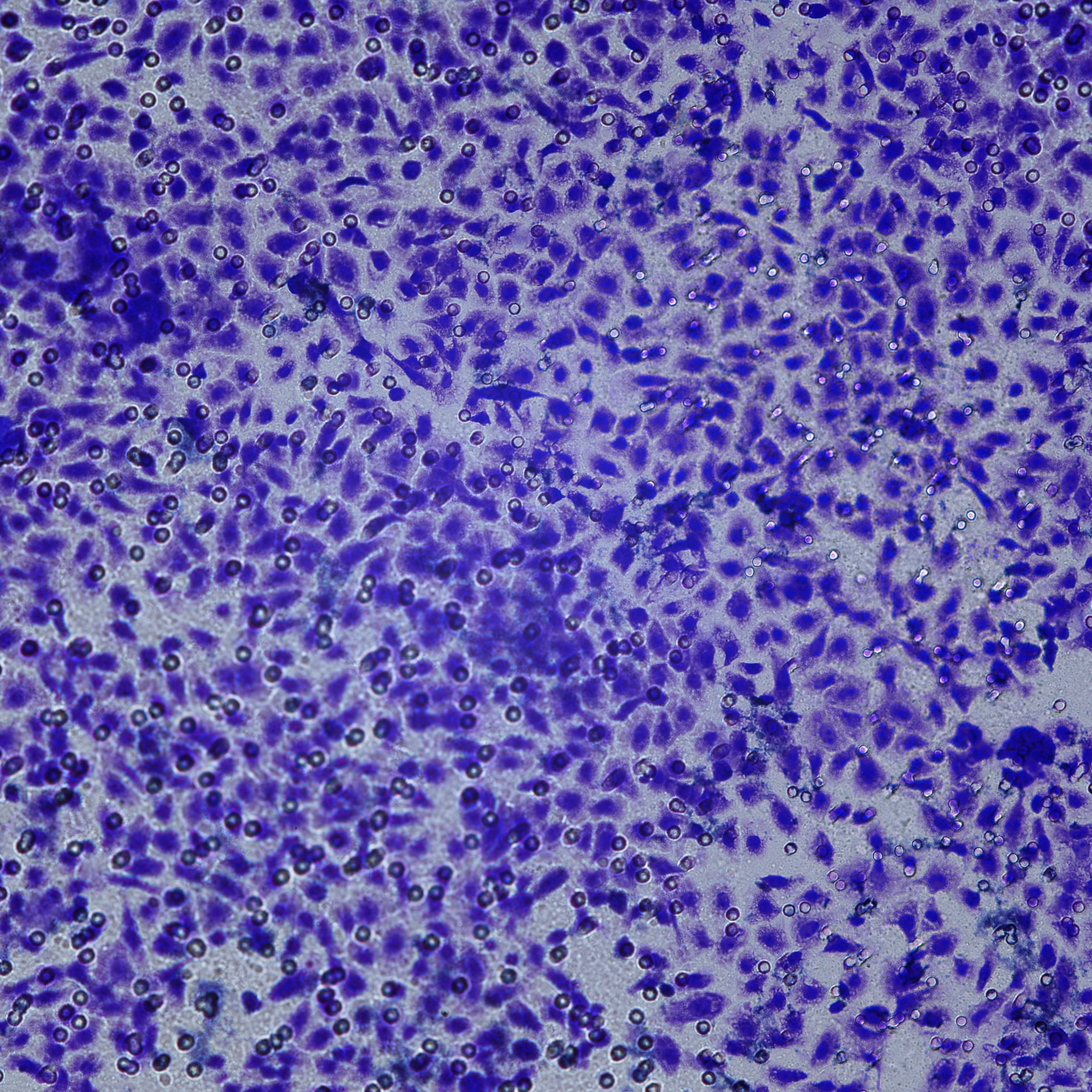

Supplement: Supplemental Information 21 [file peerj-14-21436-s021.zip › Figure 12 B Transwell/Transwell invasion/NC + UBE2C.jpg]

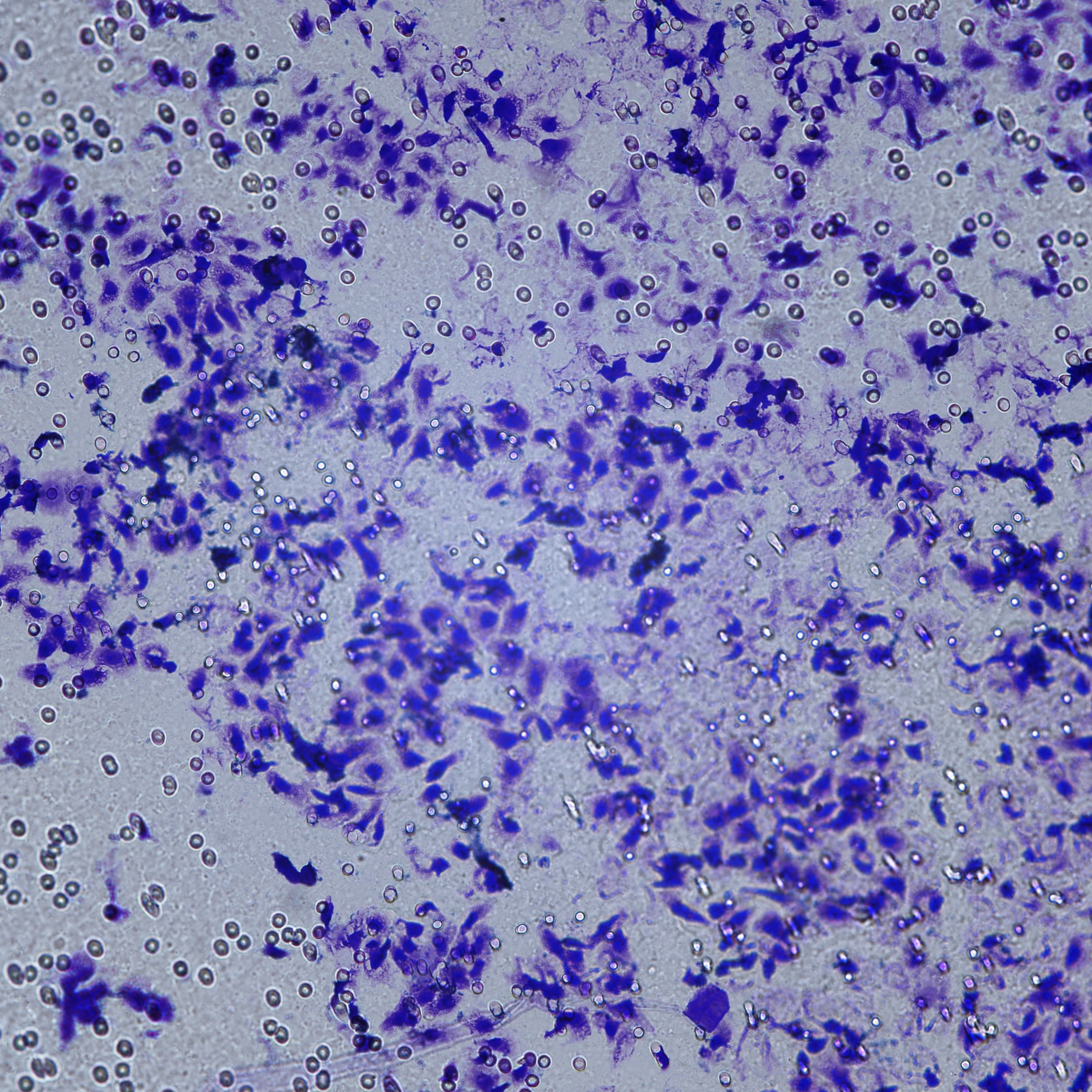

Supplement: Supplemental Information 21 [file peerj-14-21436-s021.zip › Figure 12 B Transwell/Transwell invasion/NC+Vector.jpg]

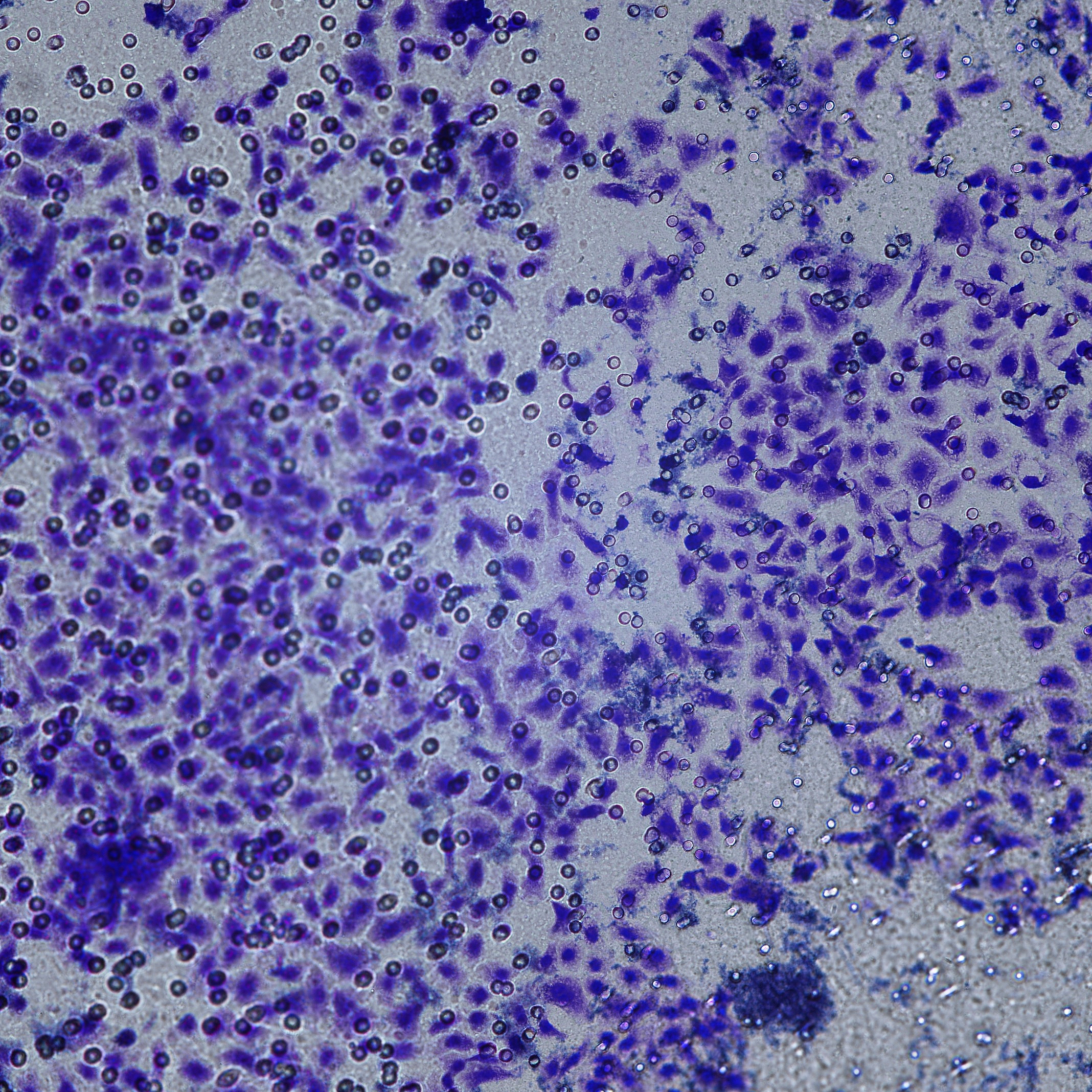

Supplement: Supplemental Information 21 [file peerj-14-21436-s021.zip › Figure 12 B Transwell/Transwell invasion/shUBE2C + UBE2C.jpg]

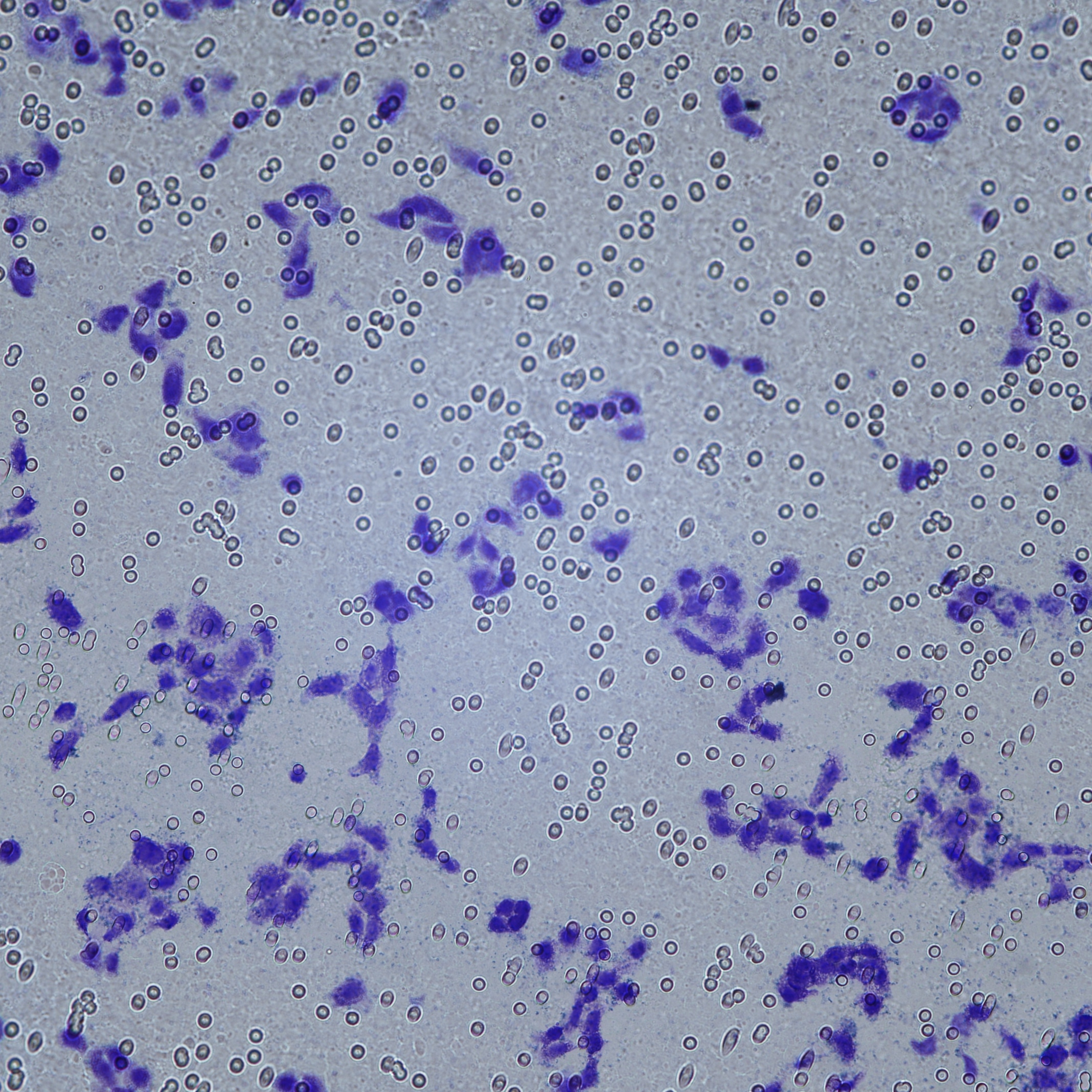

Supplement: Supplemental Information 21 [file peerj-14-21436-s021.zip › Figure 12 B Transwell/Transwell invasion/shUBE2C + Vector.jpg]

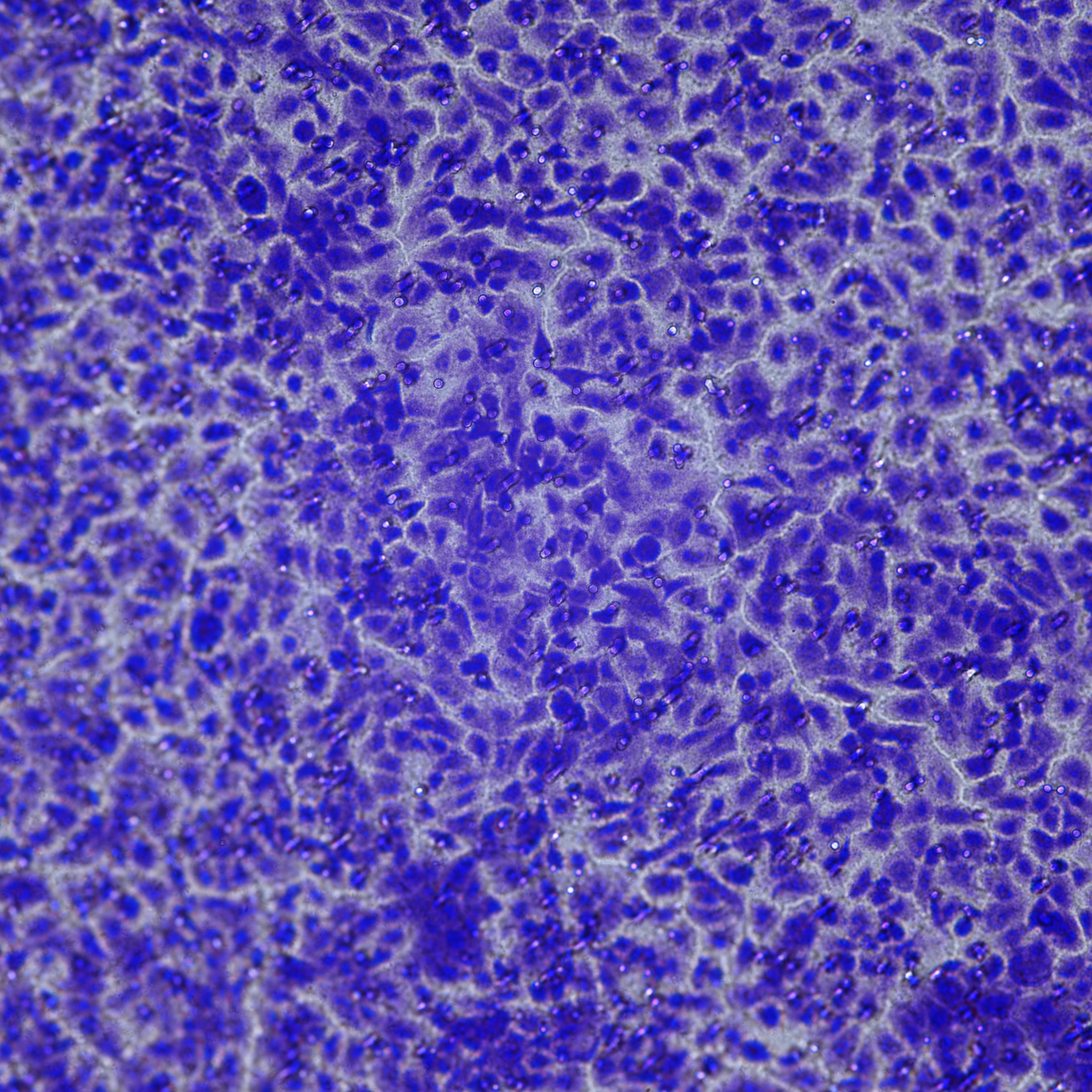

Supplement: Supplemental Information 21 [file peerj-14-21436-s021.zip › Figure 12 B Transwell/Transwell migration/NC + UBE2C.jpg]

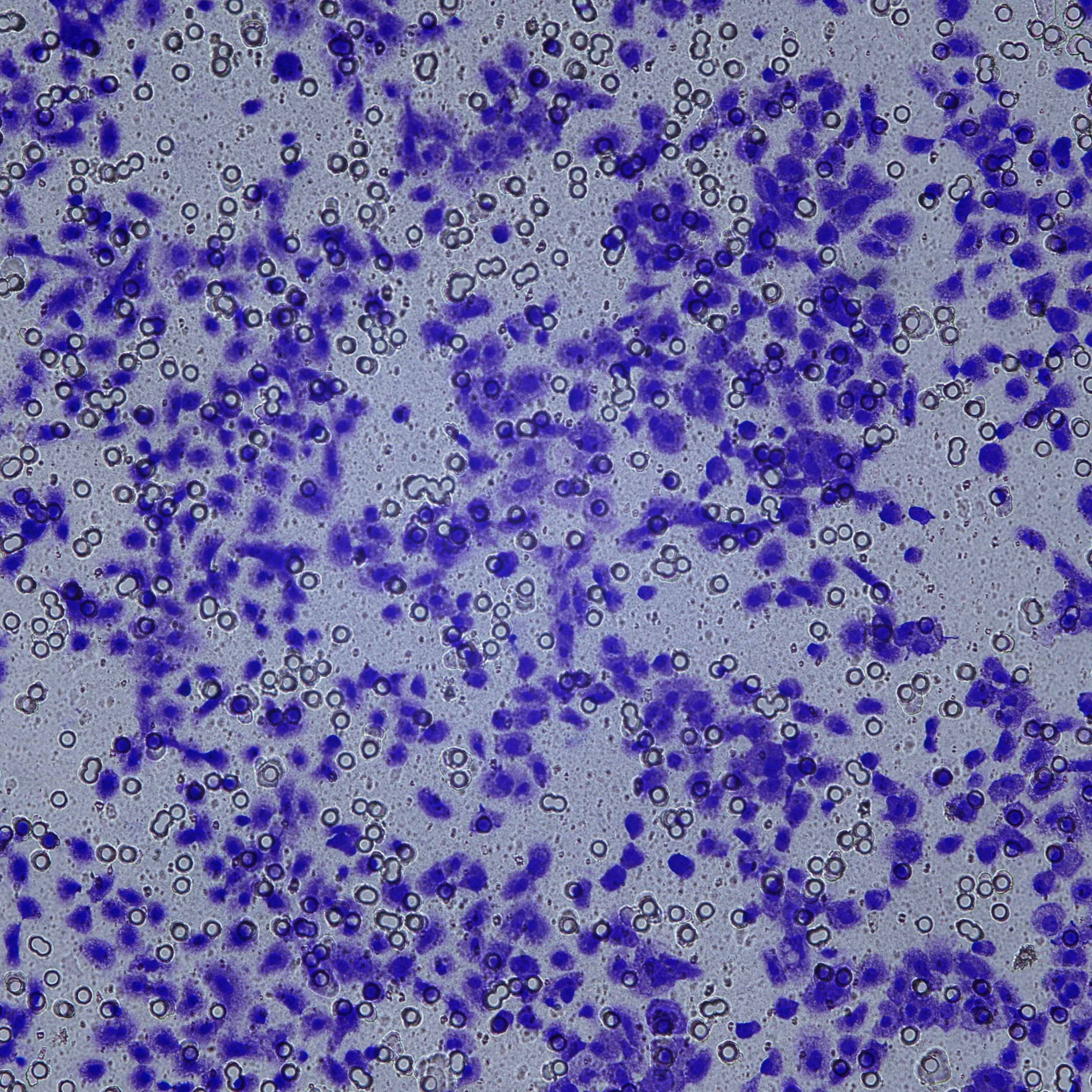

Supplement: Supplemental Information 21 [file peerj-14-21436-s021.zip › Figure 12 B Transwell/Transwell migration/NC+Vector.jpg]

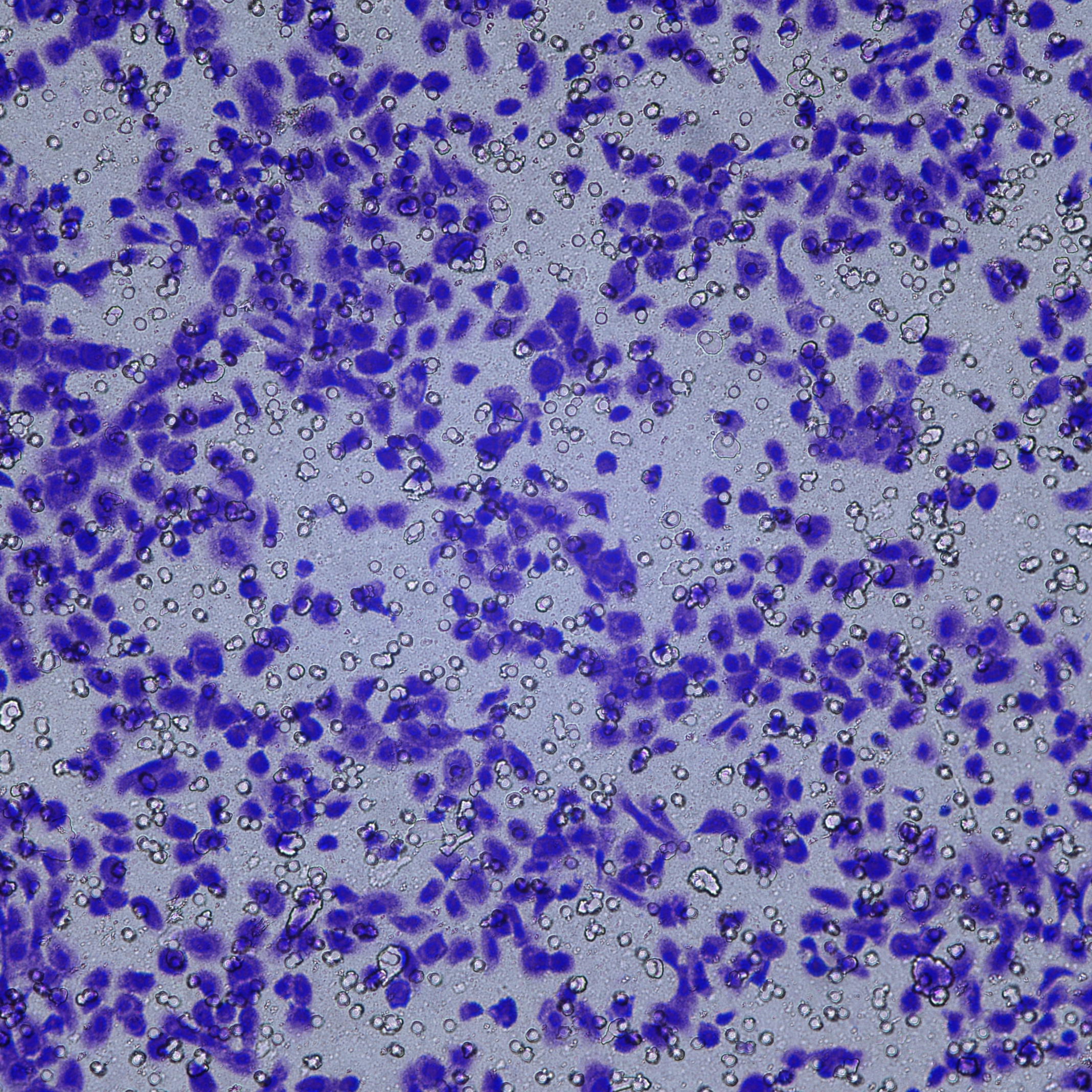

Supplement: Supplemental Information 21 [file peerj-14-21436-s021.zip › Figure 12 B Transwell/Transwell migration/shUBE2C + UBE2C.jpg]

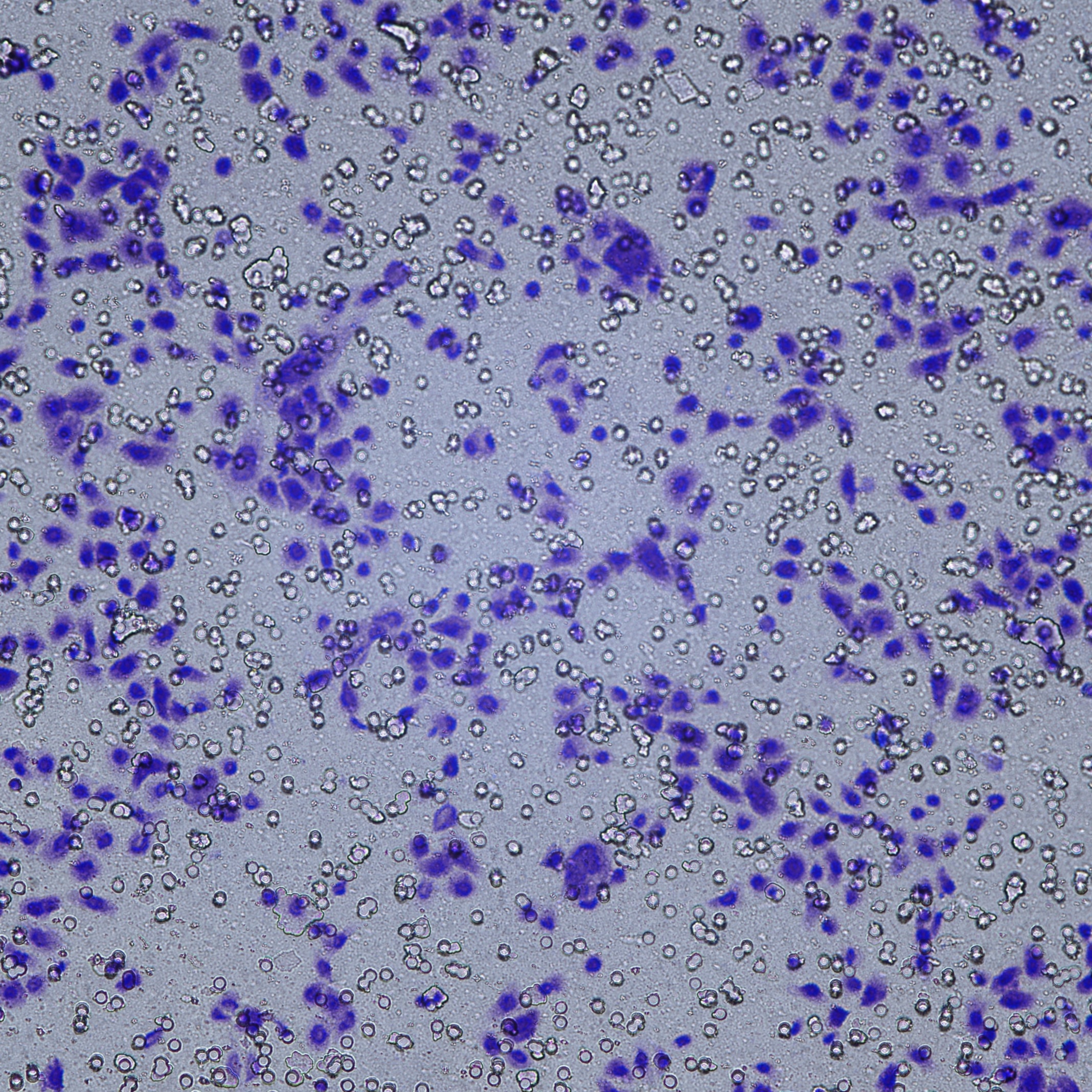

Supplement: Supplemental Information 21 [file peerj-14-21436-s021.zip › Figure 12 B Transwell/Transwell migration/shUBE2C + Vector.jpg]

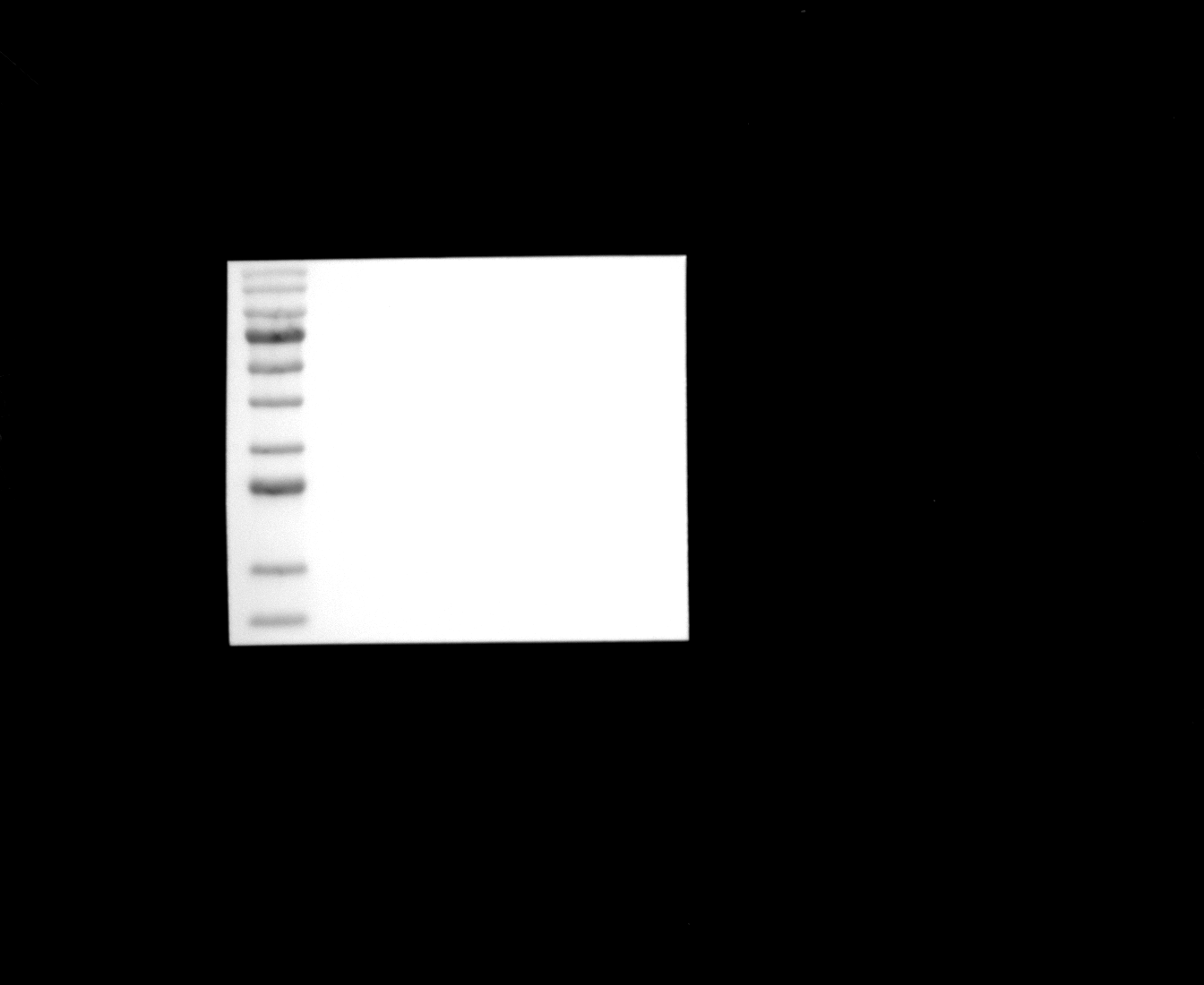

Supplement: Supplemental Information 22 [file peerj-14-21436-s022.zip › Figure 12 C Western Blot/1-Cyclin D1/0.Tif]

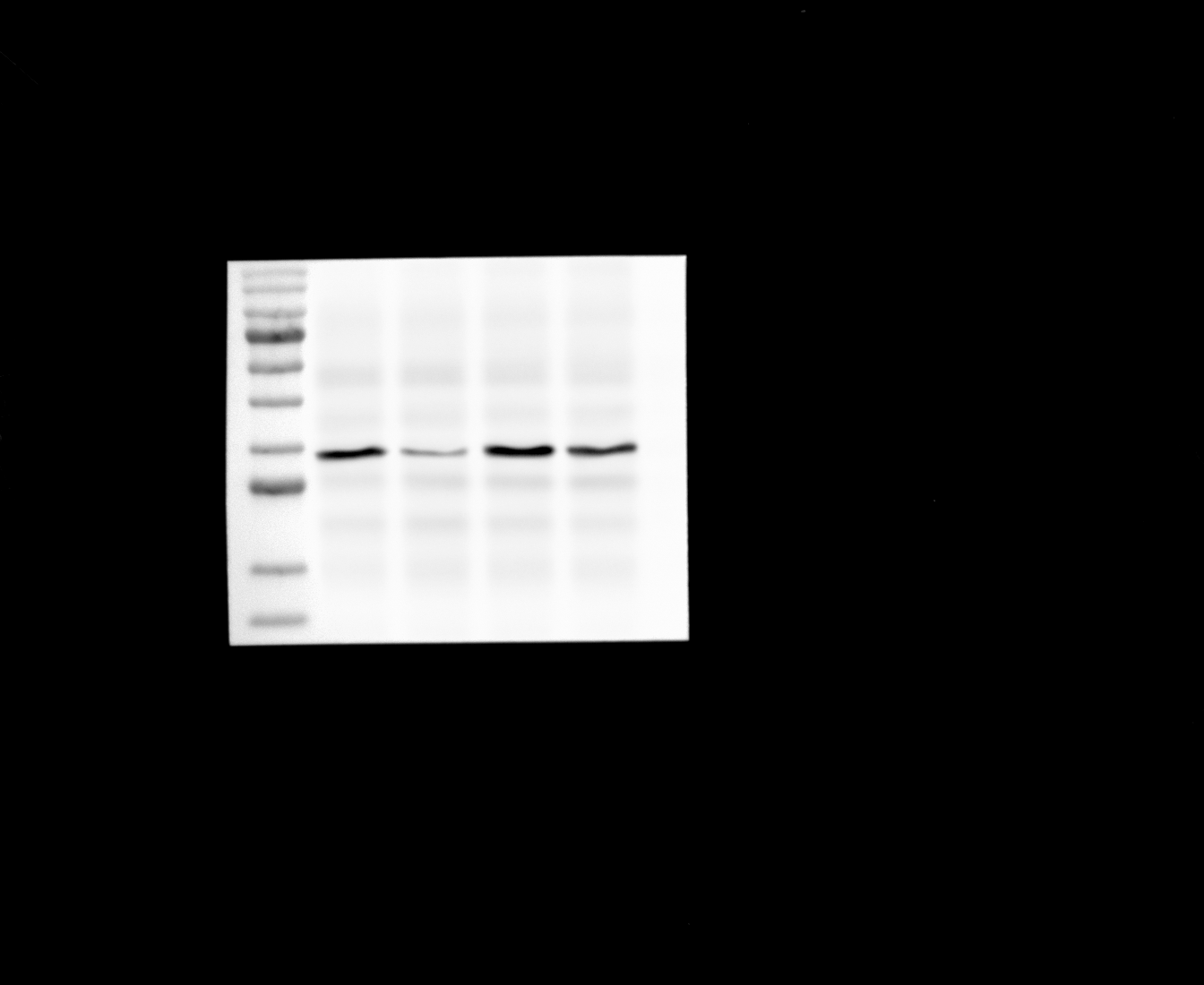

Supplement: Supplemental Information 22 [file peerj-14-21436-s022.zip › Figure 12 C Western Blot/1-Cyclin D1/1.Tif]

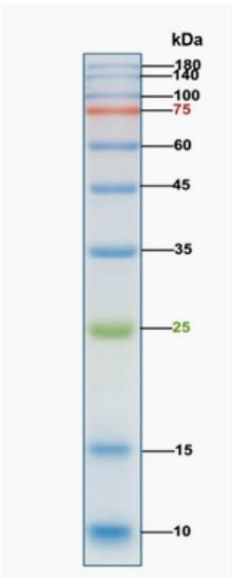

Supplement: Supplemental Information 22 [file peerj-14-21436-s022.zip › Figure 12 C Western Blot/1-Cyclin D1/Marker.tif]

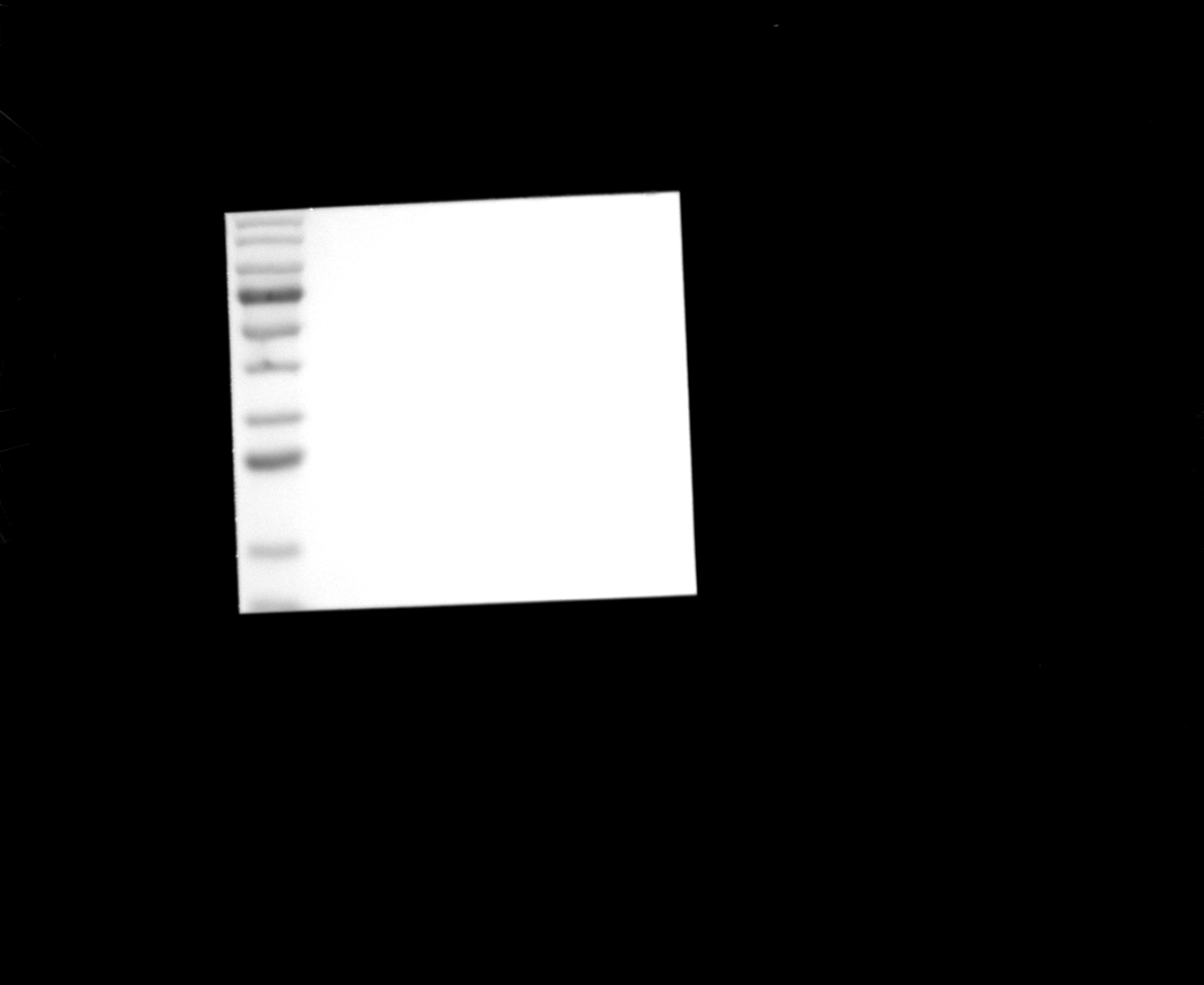

Supplement: Supplemental Information 22 [file peerj-14-21436-s022.zip › Figure 12 C Western Blot/2-P21/0.Tif]

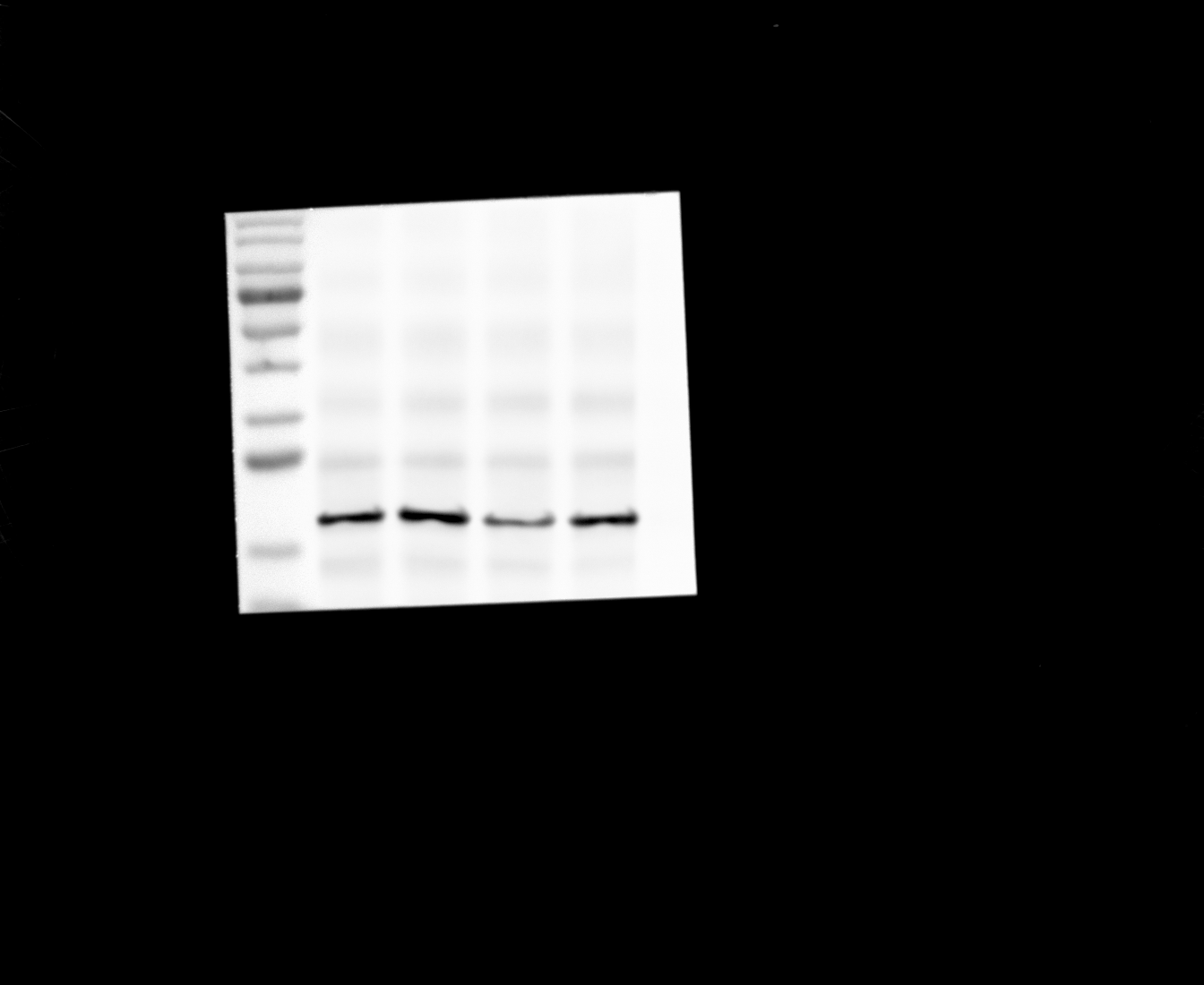

Supplement: Supplemental Information 22 [file peerj-14-21436-s022.zip › Figure 12 C Western Blot/2-P21/1.Tif]

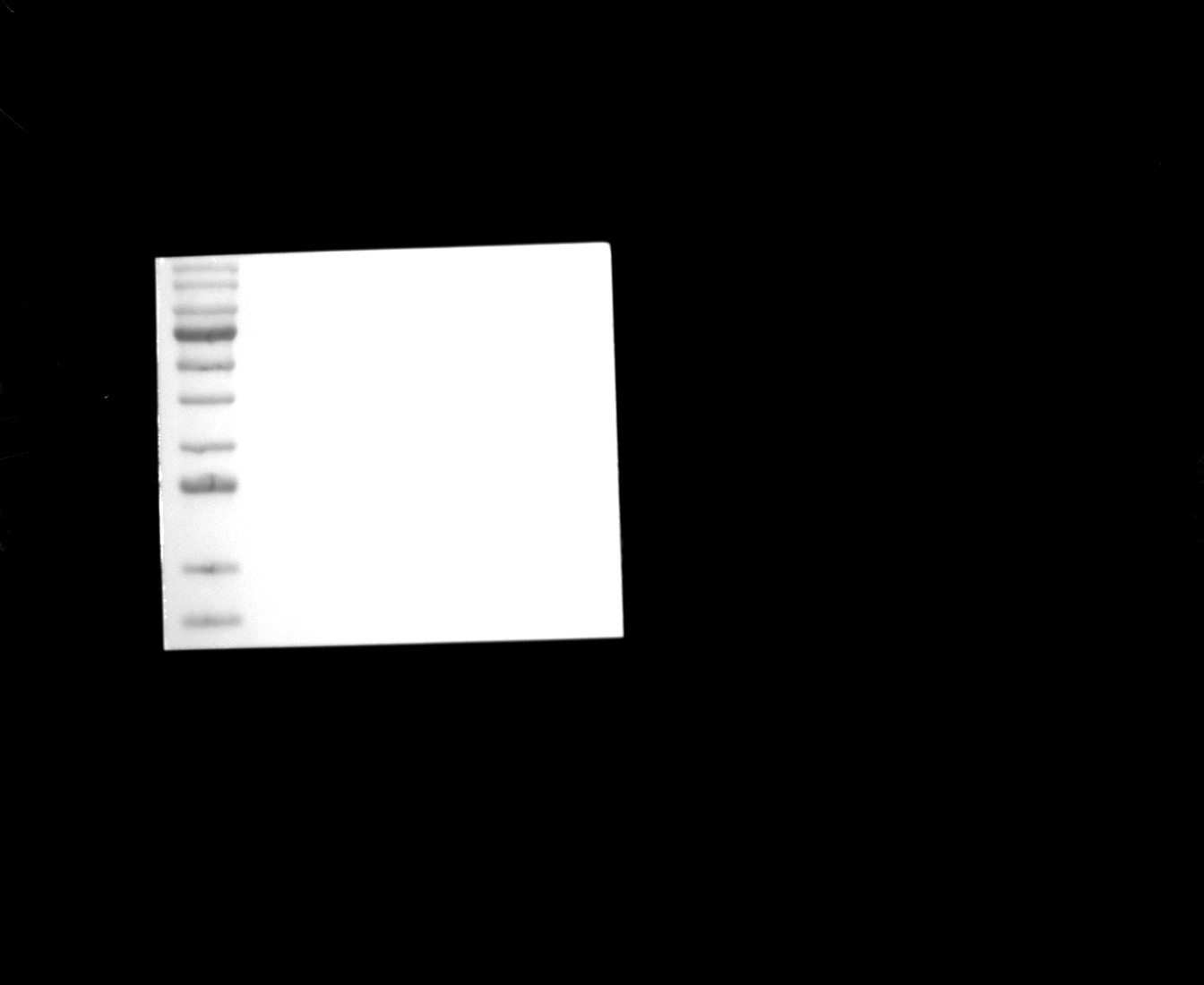

Supplement: Supplemental Information 22 [file peerj-14-21436-s022.zip › Figure 12 C Western Blot/3-CDK4/0.Tif]

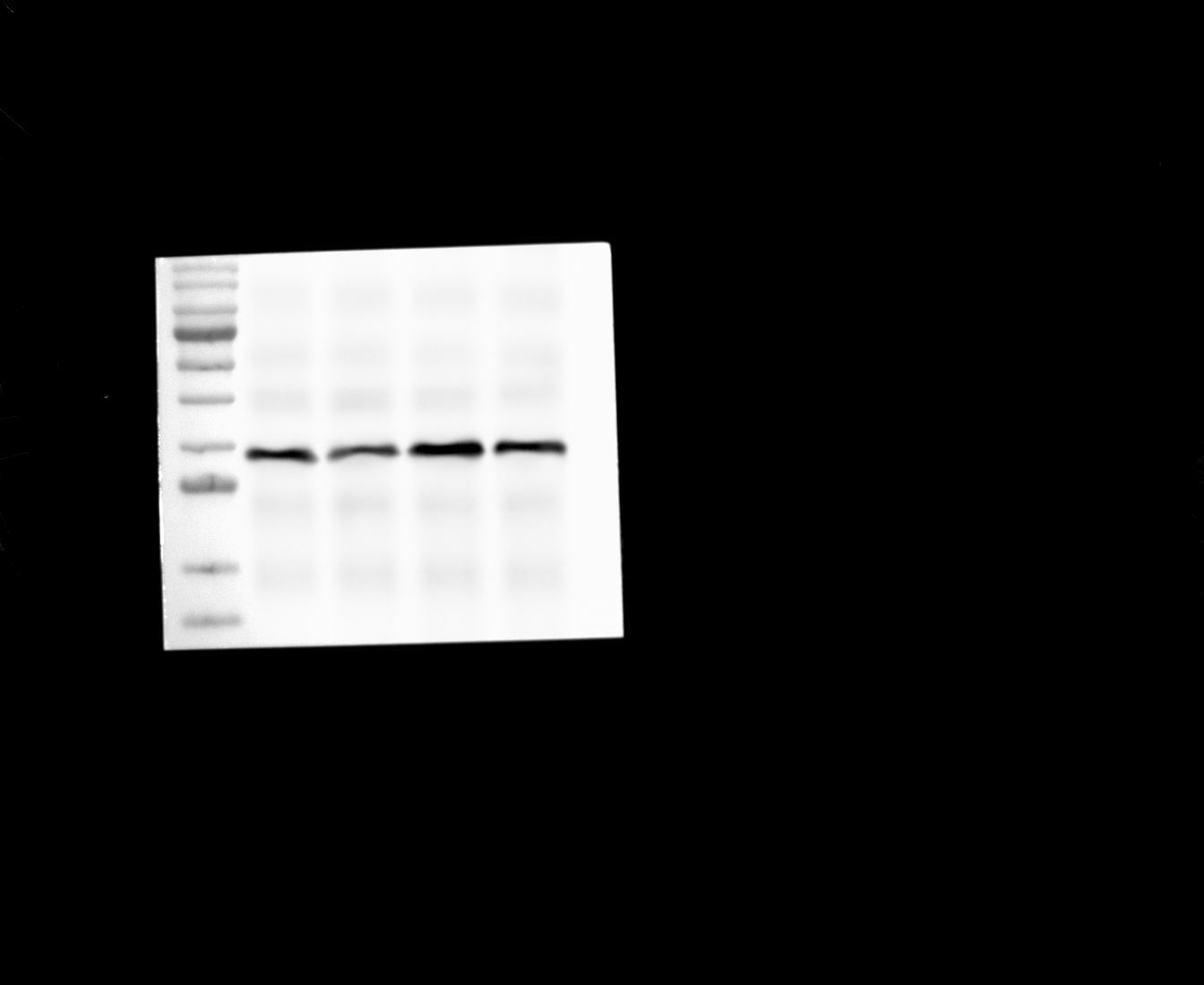

Supplement: Supplemental Information 22 [file peerj-14-21436-s022.zip › Figure 12 C Western Blot/3-CDK4/1.Tif]

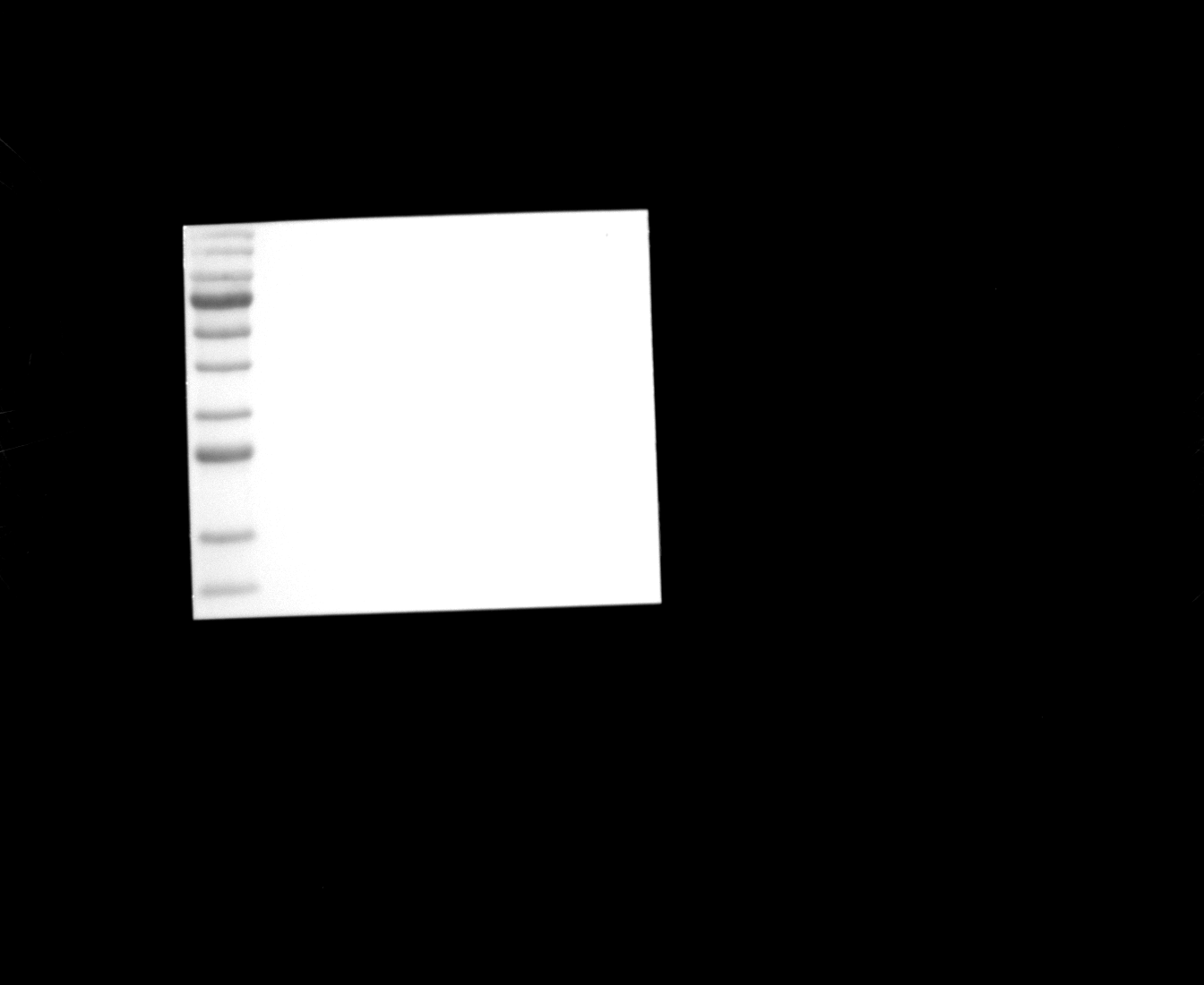

Supplement: Supplemental Information 22 [file peerj-14-21436-s022.zip › Figure 12 C Western Blot/4-Bcl-2/0.Tif]

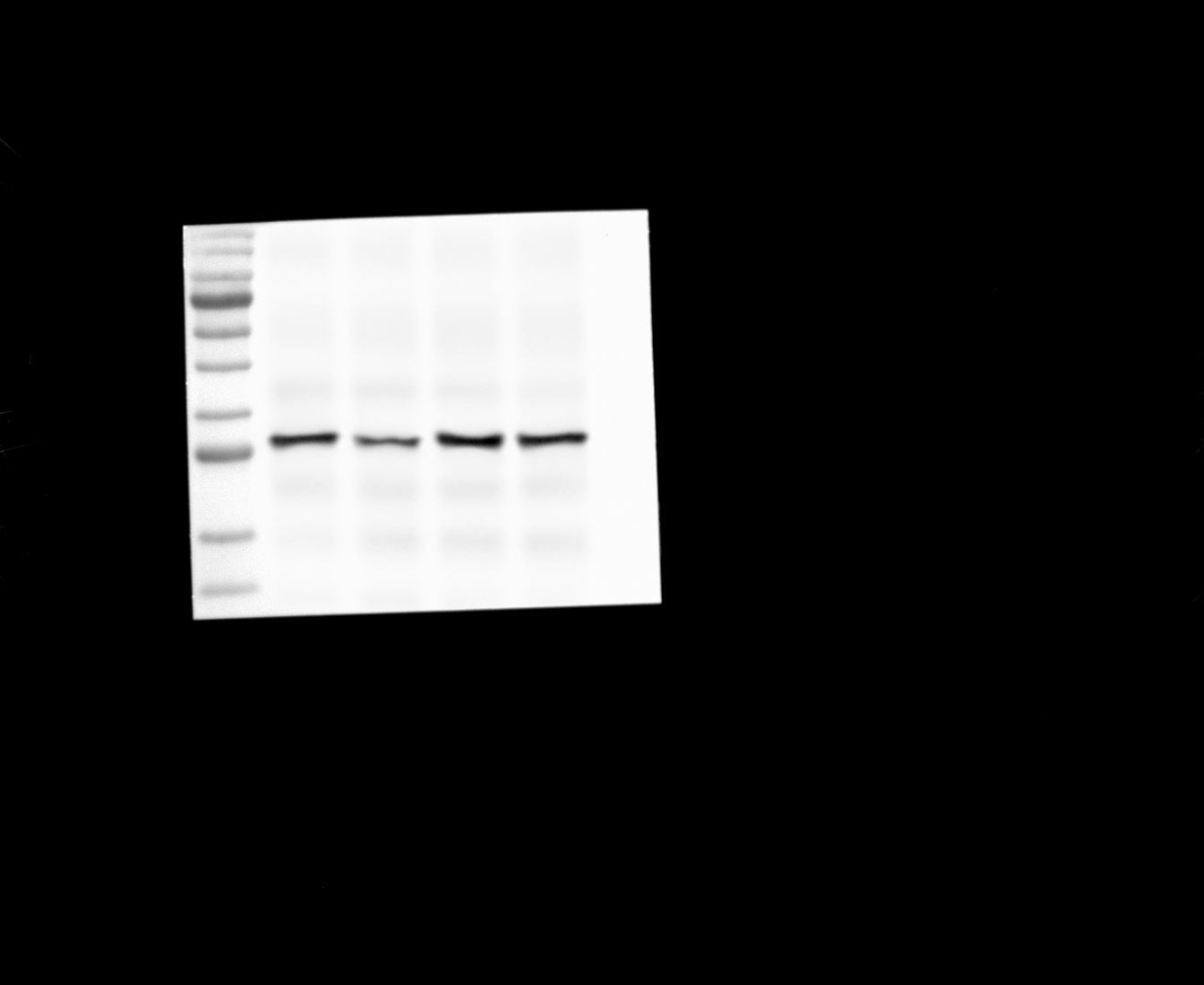

Supplement: Supplemental Information 22 [file peerj-14-21436-s022.zip › Figure 12 C Western Blot/4-Bcl-2/1.Tif]

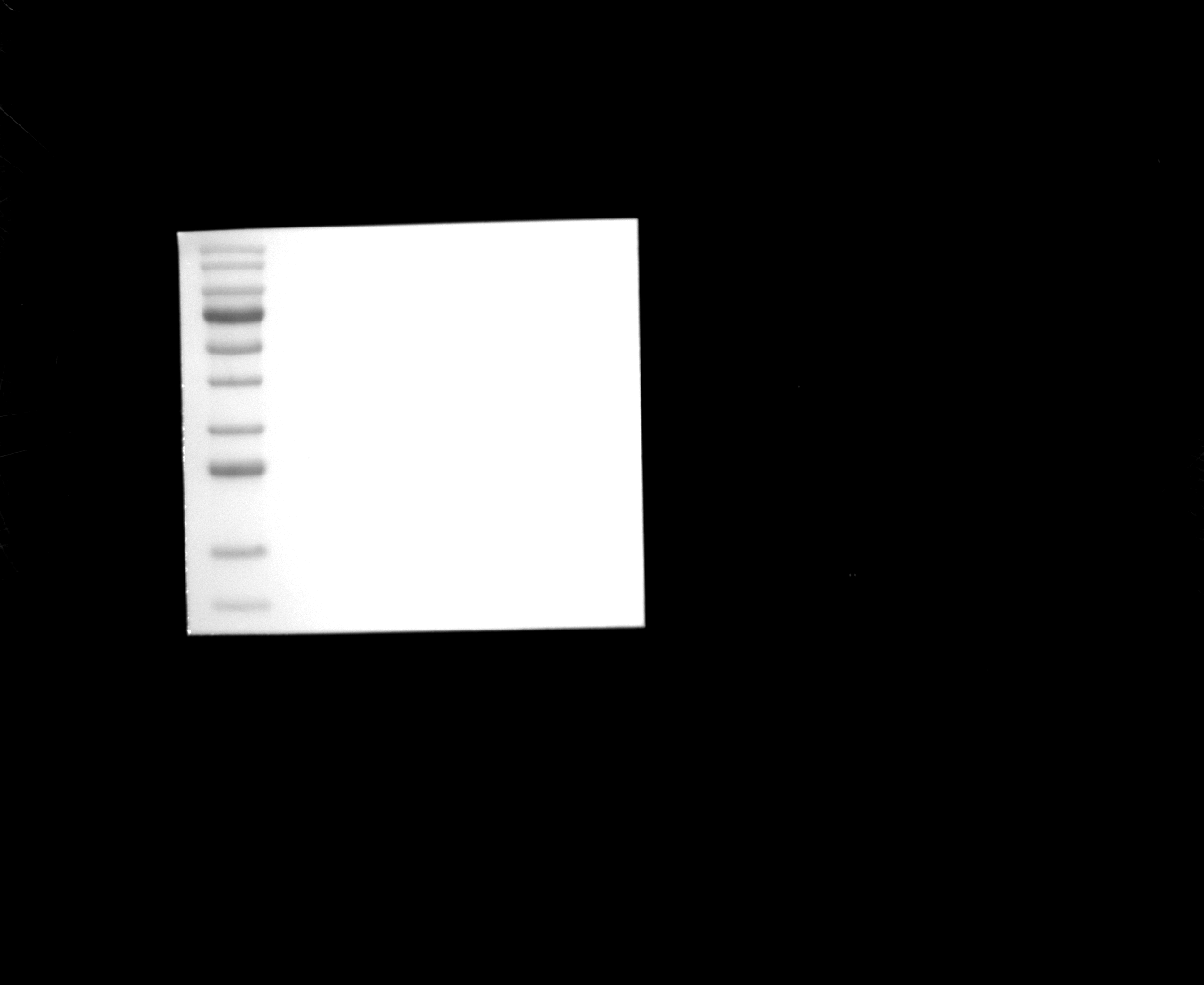

Supplement: Supplemental Information 22 [file peerj-14-21436-s022.zip › Figure 12 C Western Blot/5-Bax/0.Tif]

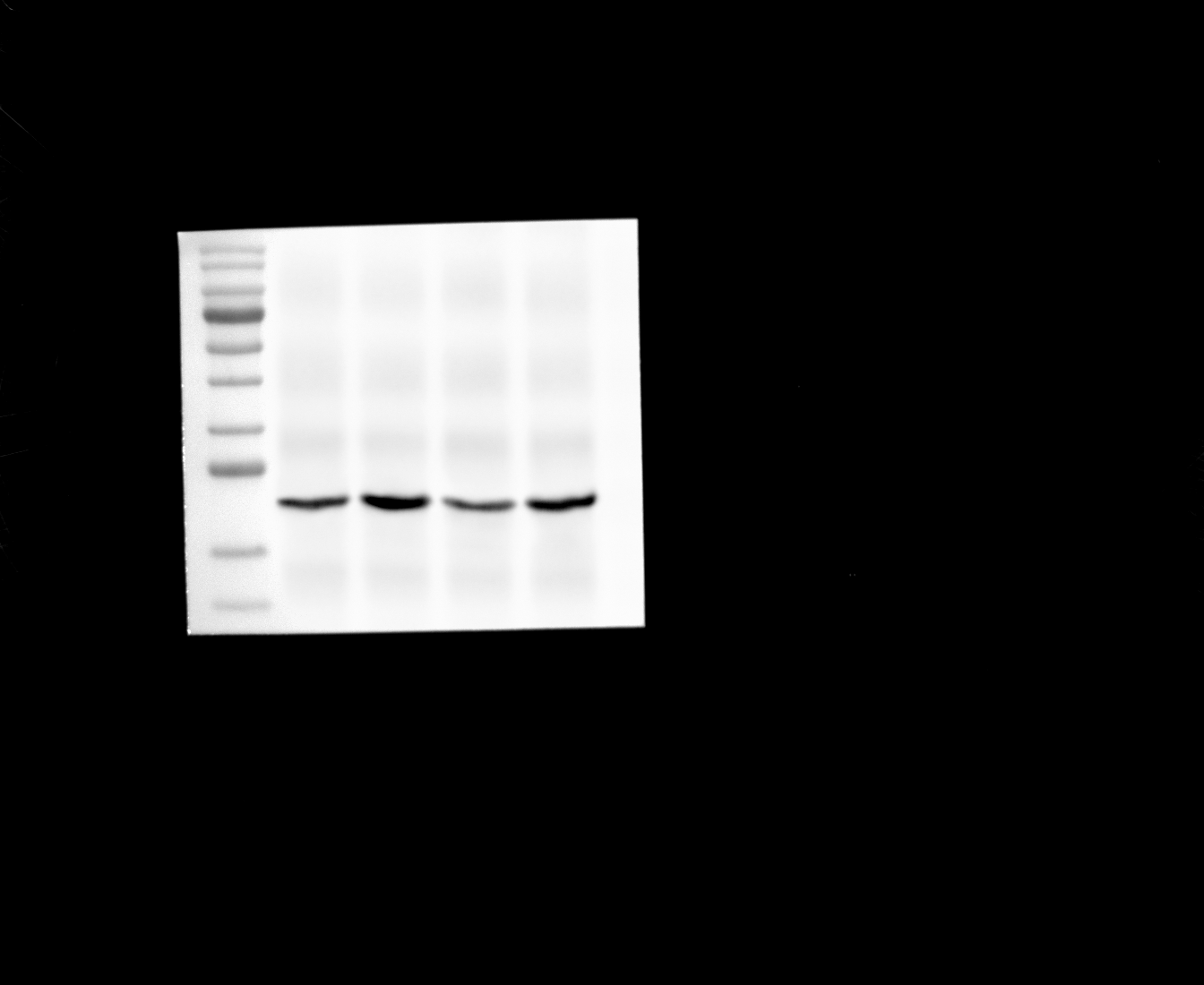

Supplement: Supplemental Information 22 [file peerj-14-21436-s022.zip › Figure 12 C Western Blot/5-Bax/1.Tif]

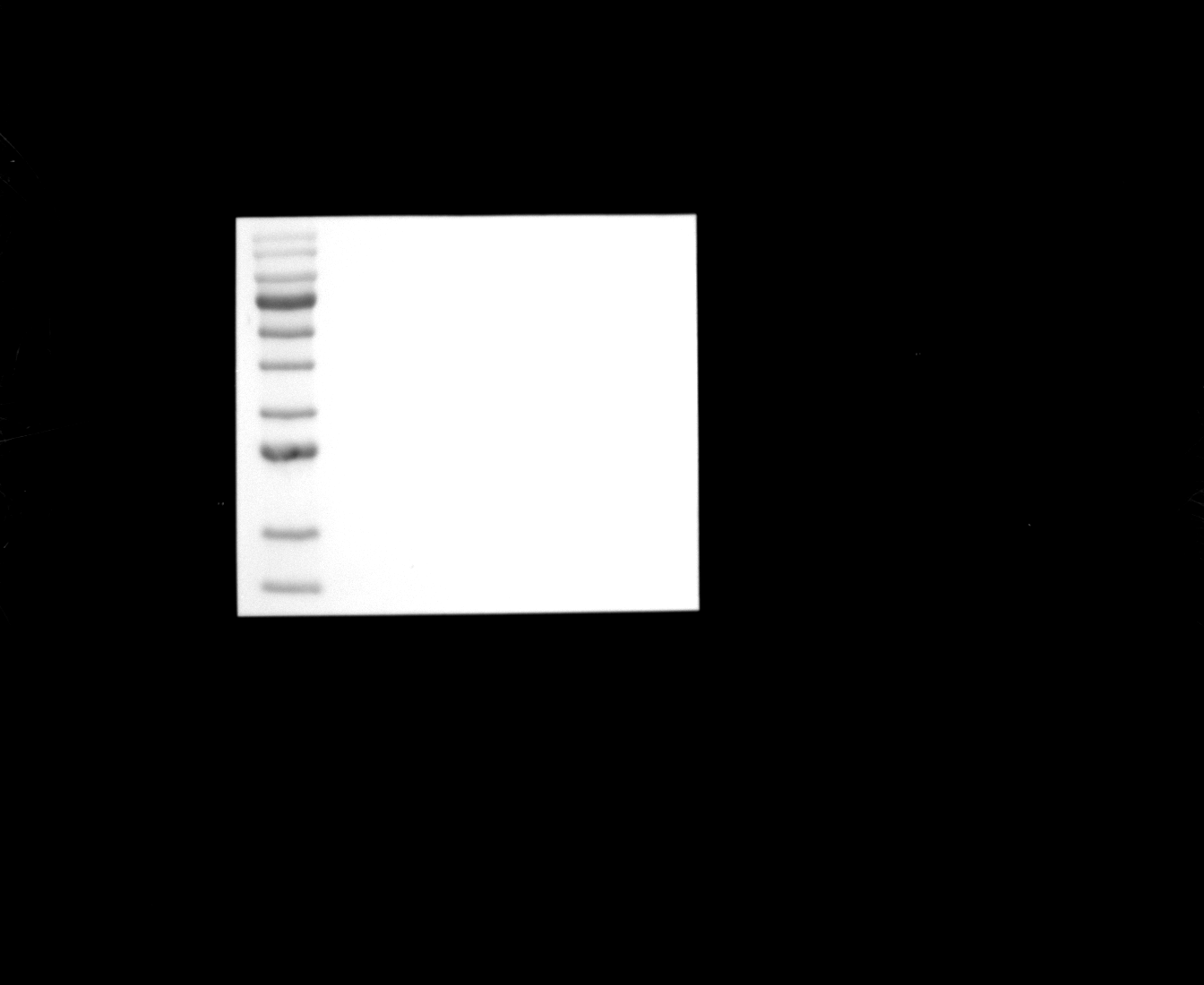

Supplement: Supplemental Information 22 [file peerj-14-21436-s022.zip › Figure 12 C Western Blot/6-Cleaved-Caspase-3/0.Tif]

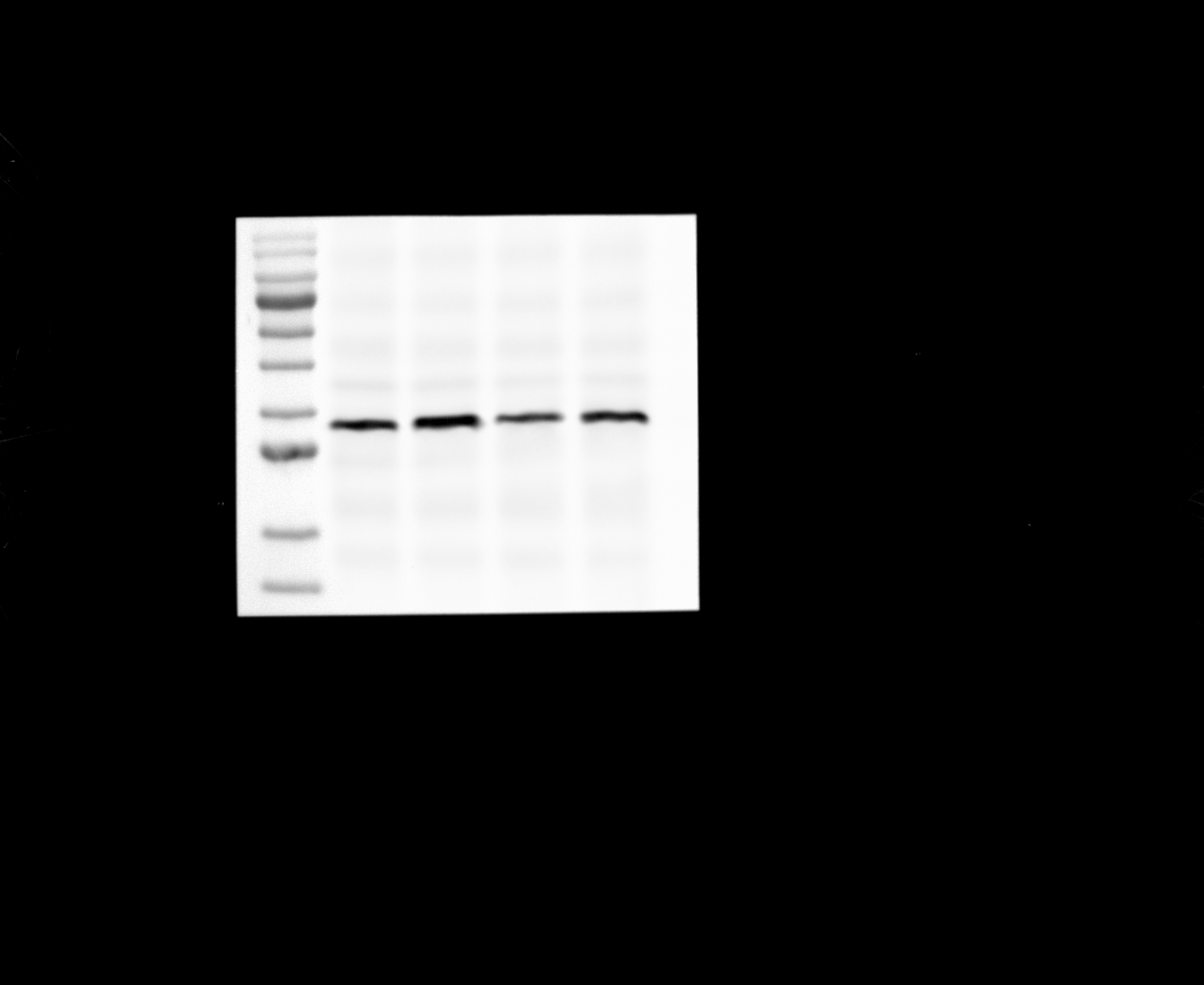

Supplement: Supplemental Information 22 [file peerj-14-21436-s022.zip › Figure 12 C Western Blot/6-Cleaved-Caspase-3/1.Tif]

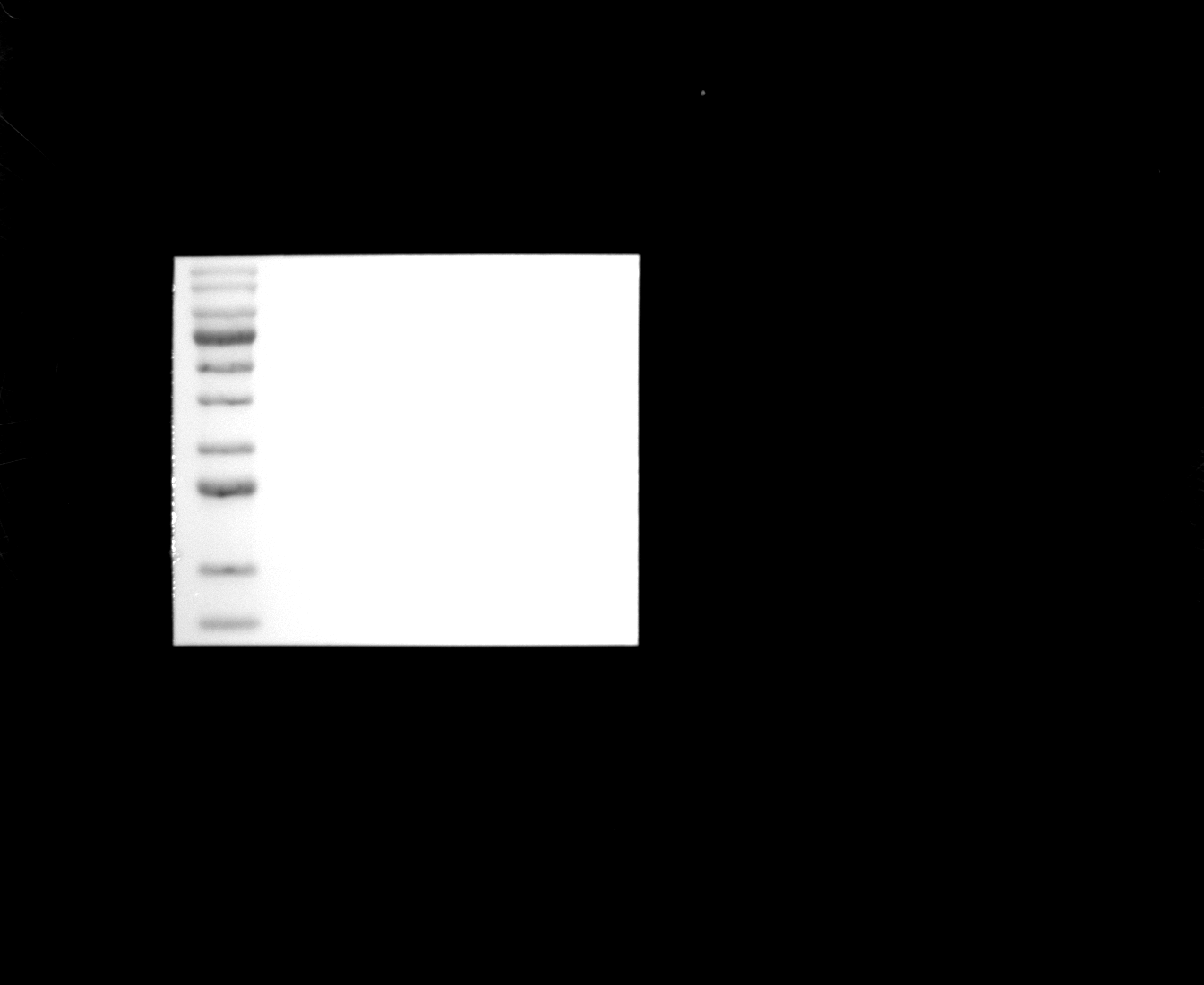

Supplement: Supplemental Information 22 [file peerj-14-21436-s022.zip › Figure 12 C Western Blot/7-UBE2C/0.Tif]

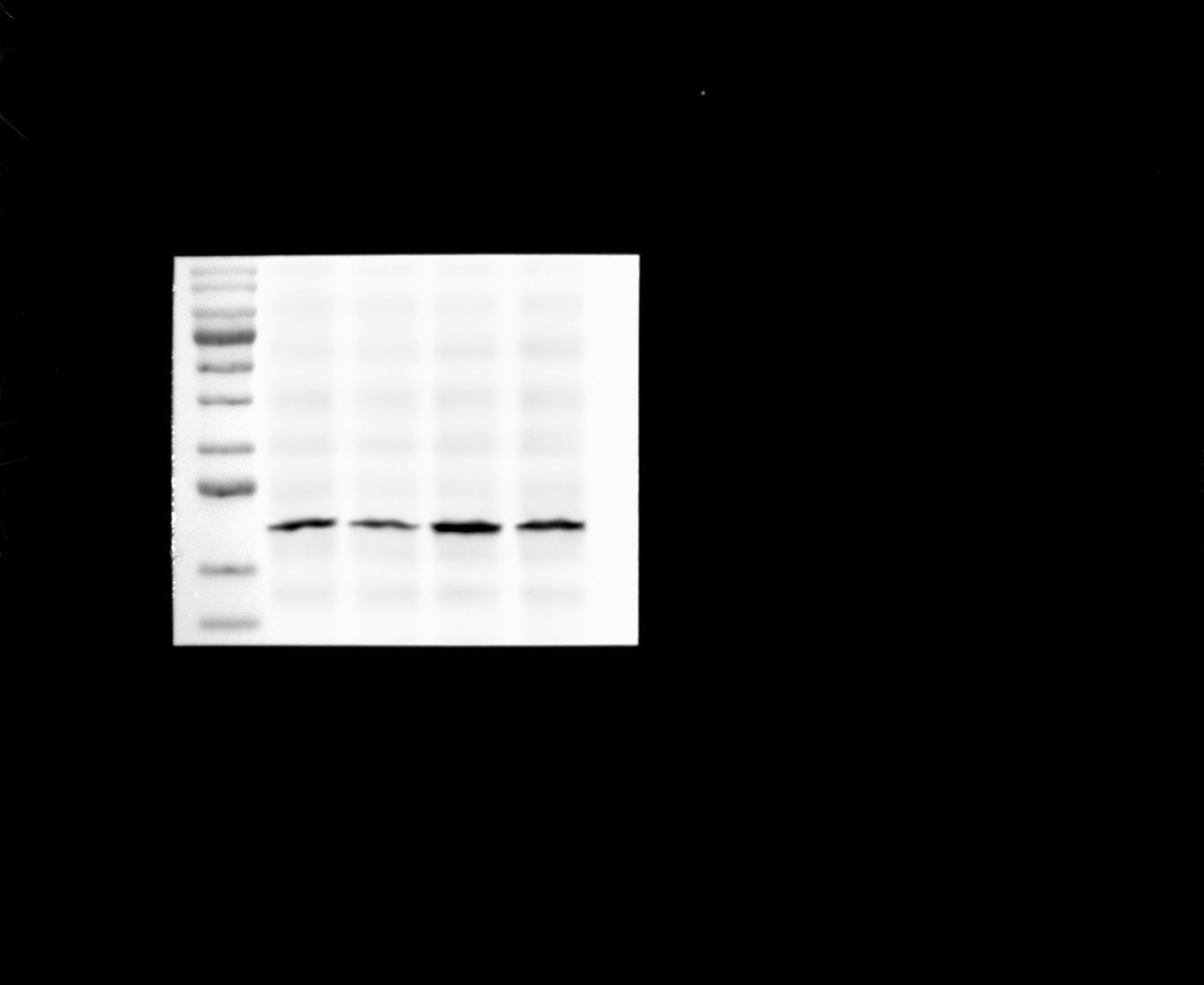

Supplement: Supplemental Information 22 [file peerj-14-21436-s022.zip › Figure 12 C Western Blot/7-UBE2C/1.Tif]

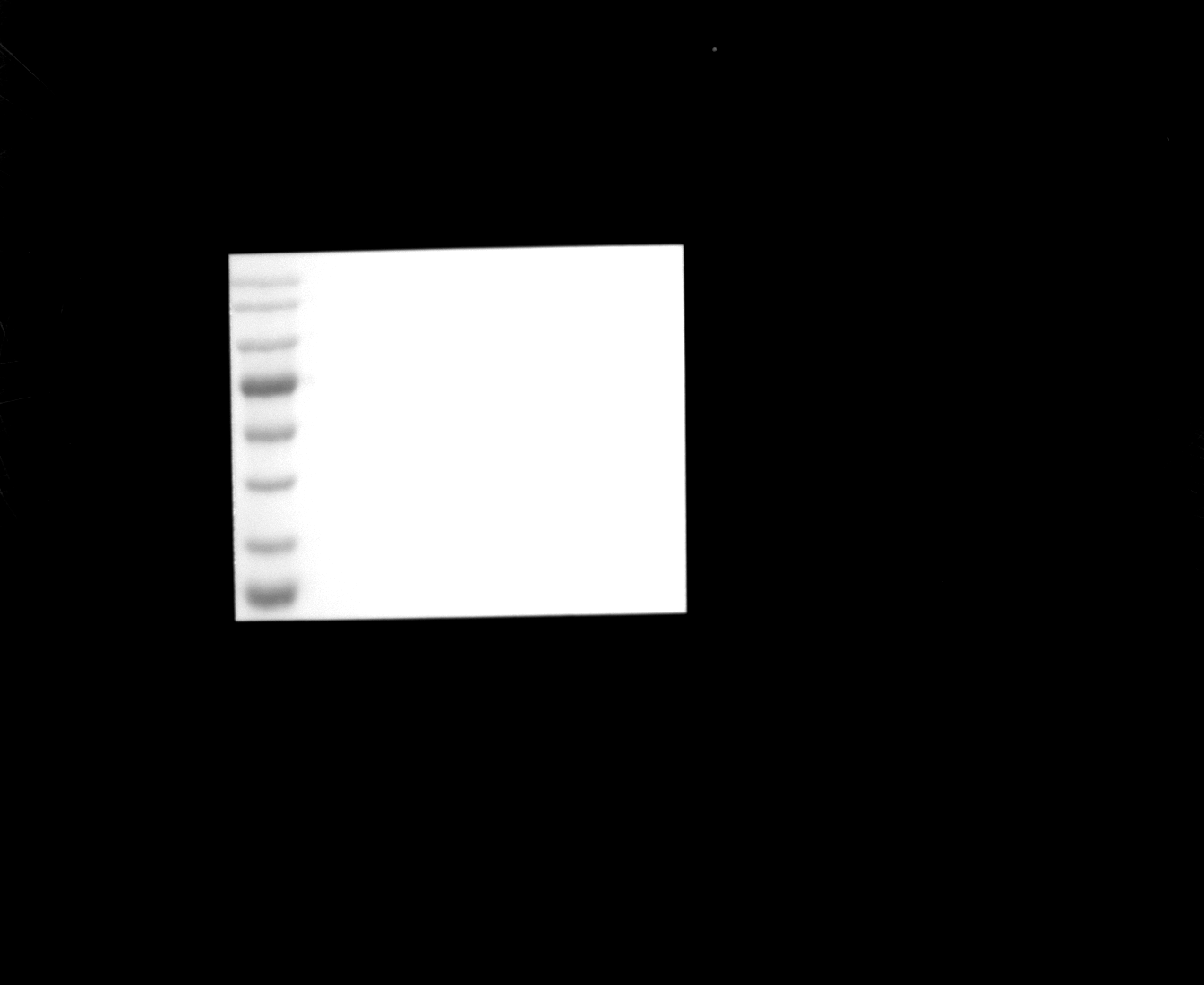

Supplement: Supplemental Information 22 [file peerj-14-21436-s022.zip › Figure 12 C Western Blot/8-β-Tubulin/0.Tif]

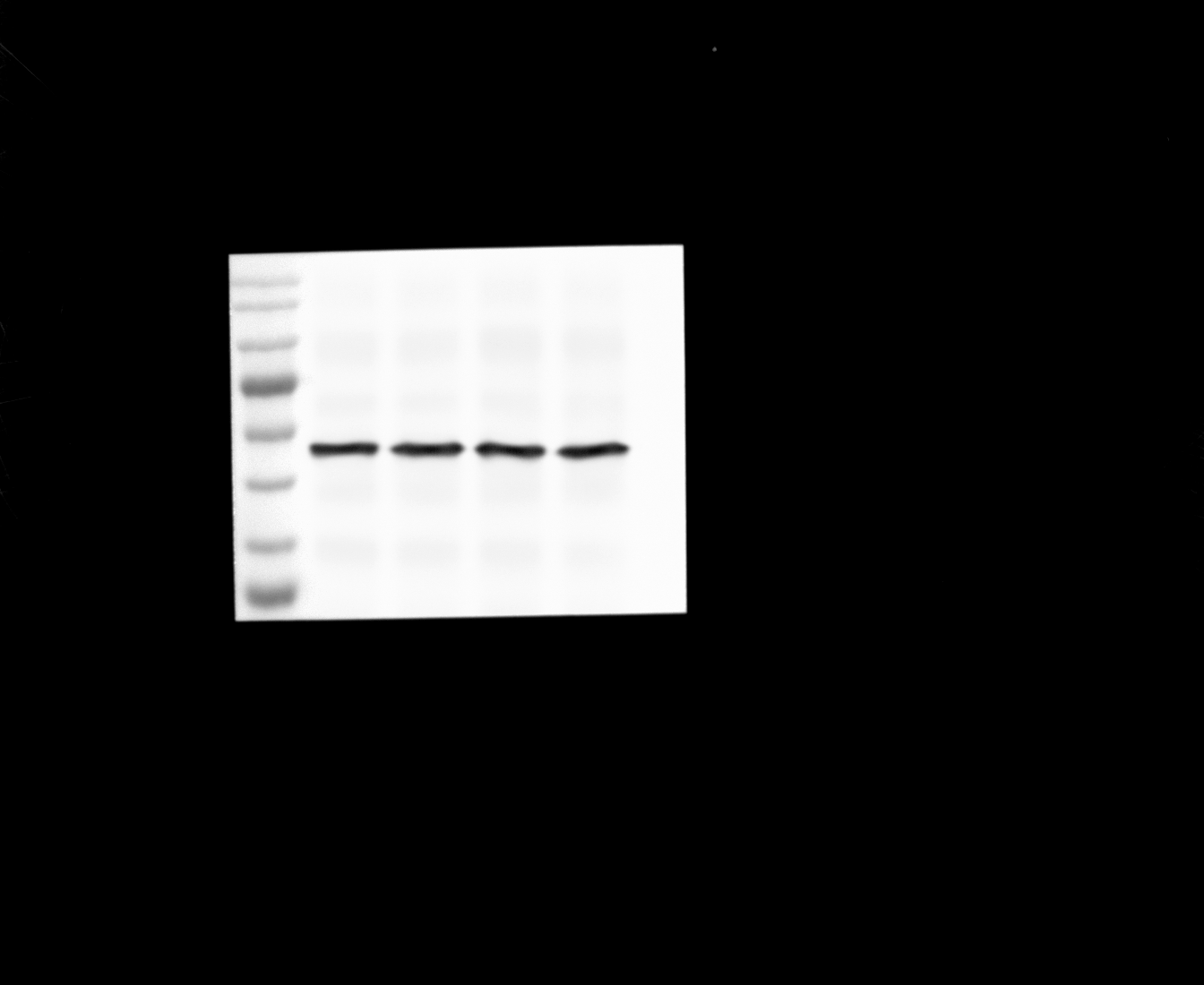

Supplement: Supplemental Information 22 [file peerj-14-21436-s022.zip › Figure 12 C Western Blot/8-β-Tubulin/1.Tif]
