# Supplementary material for: UBE2C contributes to malignant phenotypes in clear cell renal cell carcinoma via cell cycle and apoptosis regulation
Source: PeerJ. 2026 Jun 18;14:e21436. doi: 10.7717/peerj.21436 (PMC13283363; doi:10.7717/peerj.21436)
Supplement: Supplemental Information 13 [file peerj-14-21436-s013.docx]

**STROBE-MR checklist of recommended items to address in reports of Mendelian randomization studies**^1^ ^2^

| **Item No.** | **Section** | **Checklist item** | **Page No.** | **Relevant text from manuscript** |
| --- | --- | --- | --- | --- |
| 1 | **TITLE and ABSTRACT** | Indicate Mendelian randomization (MR) as the study’s design in the title and/or the abstract if that is a main purpose of the study | Title page & Abstract | Title: "UBE2C Acts as a Causal Oncogene in Clear Cell Renal Cell Carcinoma by Accelerating Cell Cycle Progression and Inhibiting Apoptosis" (MR not in title); Abstract: "Mendelian randomization (MR) was used to test causal relationships". |
|  | **INTRODUCTION** |  |  |  |
| 2 | **Background** | Explain the scientific background and rationale for the reported study. What is the exposure? Is a potential causal relationship between exposure and outcome plausible? Justify why MR is a helpful method to address the study question | 1-3 | Background: Clear cell renal cell carcinoma (ccRCC) has poor prognosis; UBE2C (ubiquitin-conjugating enzyme E2C) is linked to tumorigenesis but understudied in ccRCC. Exposure: Serum UBE2C levels; Outcome: ccRCC risk. Rationale: UBE2C overexpressed in other cancers, but causal link to ccRCC unknown. MR value: Overcomes confounding from observational studies to infer causality, complementing genomic/experimental evidence. |
| 3 | **Objectives** | State specific objectives clearly, including pre-specified causal hypotheses (if any). State that MR is a method that, under specific assumptions, intends to estimate causal effects | 3 | Objectives: 1) Assess UBE2C expression/prognostic value via multi-omics; 2) Explore causal relationships between UBE2C and ccRCC using bidirectional MR; 3) Validate UBE2C function in clinical specimens/cell lines; 4) Identify underlying mechanisms. Causal hypothesis: Higher UBE2C levels causally increase ccRCC risk. MR description: "Mendelian randomization (MR) was used to test causal relationships" (note: explicitly states MR’s purpose to estimate causal effects under assumptions). |
|  | **METHODS** |  |  |  |
| 4 | **Study design and data sources** | Present key elements of the study design early in the article. Consider including a table listing sources of data for all phases of the study. For each data source contributing to the analysis, describe the following: | 4-10 |  |
|  | a) | Setting: Describe the study design and the underlying population, if possible. Describe the setting, locations, and relevant dates, including periods of recruitment, exposure, follow-up, and data collection, when available. |  | a) Setting: Mixed study design (bioinformatics, MR, experimental validation). MR data: European populations (2021); clinical specimens: Affiliated Hospital of Youjiang Medical University for Nationalities (Baise, China); cell lines: ProCell Corporation; animal models: Vital River Laboratory Animal Technology Co., Ltd. (Beijing, China). |
|  | b) | Participants: Give the eligibility criteria, and the sources and methods of selection of participants. Report the sample size, and whether any power or sample size calculations were carried out prior to the main analysis |  | b) Participants: - TCGA-KIRC: 539 tumor/72 normal tissues; GEO GSE53757: 72 ccRCC/72 normal tissues; clinical specimens: 30 patients who underwent radical/partial nephrectomy (eligibility: ccRCC diagnosis, no prior treatment); MR: Serum UBE2C (5,365 samples), ccRCC (315,137 samples); animals: 12 male BALB/c nude mice (4-week-old). Power calculation: Animal sample size (6 per group) based on prior xenograft studies; no explicit MR power calculation reported. |
|  | c) | Describe measurement, quality control and selection of genetic variants |  | c) Genetic variants: MR - IV selection: Independent SNPs (P < 5×10⁻⁸) for serum UBE2C/ccRCC; LD clumping (r² < 0.001, 10,000 kb); F-statistic >10 for SNP strength. |
|  | d) | For each exposure, outcome, and other relevant variables, describe methods of assessment and diagnostic criteria for diseases |  | d) Exposure: Serum UBE2C (GWAS data); Outcome: ccRCC (GWAS diagnosis, clinical histopathological confirmation). Variables: mRNA/protein expression (qRT-PCR, IHC, IF), cell function (CCK-8, colony formation, etc.), apoptosis/cell cycle (flow cytometry). |
|  | e) | Provide details of ethics committee approval and participant informed consent, if relevant |  | e) Ethics approval: - Clinical study: Youjiang Medical University for Nationalities Institutional Review Board (Approval No: 2025JJH140083); - Animal study: Animal Ethics Committee of Youjiang Medical University for Nationalities (Approval No.: 2023101302); Informed consent: Obtained from all clinical participants. |
| 5 | **Assumptions** | Explicitly state the three core IV assumptions for the main analysis (relevance, independence and exclusion restriction) as well assumptions for any additional or sensitivity analysis | 7 | Core IV assumptions (implied in MR methods): 1) Relevance: SNPs significantly associated with serum UBE2C levels (P < 5×10⁻⁸); 2) Independence: SNPs not associated with confounding factors (LD clumping to ensure independence); 3) Exclusion restriction: SNPs affect ccRCC only through UBE2C. Sensitivity analysis assumptions: Assessed heterogeneity (Cochran’s Q) and horizontal pleiotropy (MR-Egger intercept, MR-PRESSO). |
| 6 | **Statistical methods: main analysis** | Describe statistical methods and statistics used | 7-8, 12 |  |
|  | a) | Describe how quantitative variables were handled in the analyses (i.e., scale, units, model) |  | a) Quantitative variables: Presented as mean ± SD; analyzed via t-tests, ANOVA, Cox regression. |
|  | b) | Describe how genetic variants were handled in the analyses and, if applicable, how their weights were selected |  | b) Genetic variants: LD clumping (r² < 0.001, 10,000 kb); weak IVs excluded (F > 10); weights based on IVW method. |
|  | c) | Describe the MR estimator (e.g. two-stage least squares, Wald ratio) and related statistics. Detail the included covariates and, in case of two-sample MR, whether the same covariate set was used for adjustment in the two samples |  | c) MR estimator: Main - Inverse-variance weighted (IVW) regression; supplements - MR-Egger, weighted median, simple mode, weighted mode. Two-sample MR: No explicit covariate adjustment reported (same covariate set not specified). |
|  | d) | Explain how missing data were addressed |  | d) Missing data: No explicit description of missing data handling. |
|  | e) | If applicable, indicate how multiple testing was addressed |  | e) Multiple testing: Not explicitly addressed (P < 0.05 considered significant). |
| 7 | **Assessment of assumptions** | Describe any methods or prior knowledge used to assess the assumptions or justify their validity | 7-8 | Assessment methods: - Relevance: F-statistic >10; - Independence: LD clumping; - Exclusion restriction: Sensitivity analyses (MR-Egger intercept test, MR-PRESSO global test); - Heterogeneity: Cochran’s Q statistic; - Prior knowledge: UBE2C’s role in ubiquitin-proteasome system supports biological plausibility of IV assumptions. |
| 8 | **Sensitivity analyses and additional analyses** | Describe any sensitivity analyses or additional analyses performed (e.g. comparison of effect estimates from different approaches, independent replication, bias analytic techniques, validation of instruments, simulations) | 7-8 | Sensitivity analyses: MR-Egger, weighted median, simple mode, weighted mode; Cochran’s Q (heterogeneity); MR-Egger intercept/MR-PRESSO (pleiotropy); leave-one-out analysis. Additional analyses: Bidirectional MR (reverse MR: ccRCC → UBE2C); experimental validation (in vitro/in vivo assays); GSEA; flow cytometry. |
| 9 | **Software and pre-registration** |  |  |  |
|  | a) | Name statistical software and package(s), including version and settings used | 12 | a) Software: R (v4.2.1), GraphPad Prism (v9.0), GSEA (v4.3.2), LightCycler® 96 system (Roche), flow cytometer (Thermo Fisher Scientific). Packages: ggplot2 (R). |
|  | b) | State whether the study protocol and details were pre-registered (as well as when and where) | 12 | b) Pre-registration: Not reported. |
|  | **RESULTS** |  |  |  |
| 10 | **Descriptive data** |  | 12-18 |  |
|  | a) | Report the numbers of individuals at each stage of included studies and reasons for exclusion. Consider use of a flow diagram |  | a) Sample flow: - TCGA-KIRC: 539 tumor/72 normal; GSE53757:72 ccRCC/72 normal; clinical:30 paired tissues; MR: Serum UBE2C (5,365)/ccRCC (315,137); animals:12 → 6 per group. Exclusion reasons: MR - weak IVs, LD clumping; cell lines - mycoplasma-positive (excluded). No flow diagram provided. |
|  | b) | Report summary statistics for phenotypic exposure(s), outcome(s), and other relevant variables (e.g. means, SDs, proportions) |  | b) Summary statistics: - UBE2C expression: TCGA (t=15.592, P<0.001), GSE53757 (t=9.717, P<0.001); survival (HR=2.25, 95% CI 1.64–3.09, P<0.001); MR (IVW OR=1.418, 95% CI 1.085–1.853, P=0.010). |
|  | c) | If the data sources include meta-analyses of previous studies, provide the assessments of heterogeneity across these studies |  | c) Heterogeneity assessment: MR (IVW Q=22.169, P=0.178); GSEA (FDR q-value <0.25). |
|  | d) | For two-sample MR:  i.  Provide justification of the similarity of the genetic variant-exposure associations between the exposure and outcome samples  ii.  Provide information on the number of individuals who overlap between the exposure and outcome studies |  | d) Two-sample MR: i. Similarity justification: Both datasets from European populations (2021); no explicit genetic variant-exposure association similarity data. ii. Overlap: Not reported. |
| 11 | **Main results** |  | 16-17 |  |
|  | a) | Report the associations between genetic variant and exposure, and between genetic variant and outcome, preferably on an interpretable scale |  | a) Genetic variant associations: 18 SNPs associated with serum UBE2C, 9 SNPs associated with ccRCC (P < 5×10⁻⁸). |
|  | b) | Report MR estimates of the relationship between exposure and outcome, and the measures of uncertainty from the MR analysis, on an interpretable scale, such as odds ratio or relative risk per SD difference |  | b) MR estimates: IVW OR=1.418 (95% CI 1.085–1.853, P=0.010) for serum UBE2C → ccRCC; reverse MR OR=0.985 (95% CI 0.939–1.032, P=0.519). |
|  | c) | If relevant, consider translating estimates of relative risk into absolute risk for a meaningful time period |  | c) Absolute risk translation: Not performed. |
|  | d) | Consider plots to visualize results (e.g. forest plot, scatterplot of associations between genetic variants and outcome versus between genetic variants and exposure) |  | d) Visualization: Forest plots (Figure 2A, B), leave-one-out plots (Figure 2C-F). |
| 12 | **Assessment of assumptions** |  | 17 |  |
|  | a) | Report the assessment of the validity of the assumptions |  | a) Assumption validity: - Relevance: F-statistic >10; - Independence: LD clumping (r² <0.001); - Exclusion restriction: MR-Egger intercept (P=0.584), MR-PRESSO (P=0.172) → no pleiotropy. |
|  | b) | Report any additional statistics (e.g., assessments of heterogeneity across genetic variants, such as *I^2^*, Q statistic or E-value) |  | b) Additional statistics: Cochran’s Q (IVW:22.169, P=0.178; reverse MR: P=0.399); I² not reported; E-value not reported. |
| 13 | **Sensitivity analyses and additional analyses** |  | 16-17 |  |
|  | a) | Report any sensitivity analyses to assess the robustness of the main results to violations of the assumptions |  | a) Sensitivity to assumptions: MR-Egger, weighted median, simple mode, weighted mode → consistent with IVW (no significant violation impact). |
|  | b) | Report results from other sensitivity analyses or additional analyses |  | b) Other analyses: Experimental validation (UBE2C knockdown/overexpression affects cell proliferation/invasion/apoptosis); GSEA (apoptosis/cell cycle pathways enriched). |
|  | c) | Report any assessment of direction of causal relationship (e.g., bidirectional MR) |  | c) Bidirectional MR: UBE2C → ccRCC (significant), ccRCC → UBE2C (non-significant). |
|  | d) | When relevant, report and compare with estimates from non-MR analyses |  | d) Non-MR comparison: Bioinformatics (UBE2C overexpressed in ccRCC, prognostic marker) consistent with MR causal link. |
|  | e) | Consider additional plots to visualize results (e.g., leave-one-out analyses) |  | e) Visualization: Leave-one-out plots (Figure 2C-F), GSEA plots (Figure 9A-B), flow cytometry plots (Figure 10-11). |
|  | **DISCUSSION** |  |  |  |
| 14 | **Key results** | Summarize key results with reference to study objectives | 26-28 | Key results: 1) UBE2C upregulated in ccRCC, linked to poor prognosis (AUC=0.944, independent prognostic factor); 2) MR confirms causal role (IVW OR=1.418, P=0.010) of UBE2C in ccRCC; 3) UBE2C promotes ccRCC via cell cycle acceleration and apoptosis inhibition; 4) In vitro/in vivo validation supports oncogenic function. |
| 15 | **Limitations** | Discuss limitations of the study, taking into account the validity of the IV assumptions, other sources of potential bias, and imprecision. Discuss both direction and magnitude of any potential bias and any efforts to address them | 28-29 | Limitations: 1) Clinical validation cohort small (n=30) → limited subgroup analyses; 2) MR: Cellular origin of serum UBE2C unknown; no direct link between circulating and intratumoral UBE2C; 3) Mechanisms: Specific UBE2C substrates/E3 ligase partners in ccRCC unidentifed; 4) Bias: MR may have unmeasured confounding; experimental models may not fully recapitulate human disease; 5) Imprecision: MR sample size for serum UBE2C (5,365) smaller than ccRCC (315,137) → potential imprecision. |
| 16 | **Interpretation** |  | 28-31 |  |
|  | a) | Meaning: Give a cautious overall interpretation of results in the context of their limitations and in comparison with other studies |  | a) Meaning: UBE2C is a causal oncogene in ccRCC (consistent with other cancer studies); limitations (small clinical cohort, unknown serum UBE2C origin) urge cautious interpretation. |
|  | b) | Mechanism: Discuss underlying biological mechanisms that could drive a potential causal relationship between the investigated exposure and the outcome, and whether the gene-environment equivalence assumption is reasonable. Use causal language carefully, clarifying that IV estimates may provide causal effects only under certain assumptions |  | b) Mechanism: UBE2C regulates cell cycle (G1/S transition) and apoptosis via ubiquitin-mediated degradation of mitotic regulators (cyclin B1, securin); gene-environment equivalence plausible (UBE2C’s conserved role in cell homeostasis); causal language: "MR analysis provides genetic evidence supporting a causal role" (acknowledges IV assumptions). |
|  | c) | Clinical relevance: Discuss whether the results have clinical or public policy relevance, and to what extent they inform effect sizes of possible interventions |  | c) Clinical relevance: UBE2C as diagnostic (AUC=0.944) and prognostic marker; potential therapeutic target (PROTACs, E3 ligase interaction disruptors); serum UBE2C as non-invasive monitoring tool. |
| 17 | **Generalizability** | Discuss the generalizability of the study results (a) to other populations, (b) across other exposure periods/timings, and (c) across other levels of exposure | 31 | a) Other populations: MR data from Europeans; clinical data from Chinese cohort → need validation in diverse ethnicities. b) Exposure periods/timings: UBE2C may act as early driver (causal link), but effect of exposure timing (e.g., early vs. late tumorigenesis) unknown. c) Exposure levels: High UBE2C linked to advanced disease, but dose-response relationship (different UBE2C levels) not explored. |
|  | **OTHER INFORMATION** |  |  |  |
| 18 | **Funding** | Describe sources of funding and the role of funders in the present study and, if applicable, sources of funding for the databases and original study or studies on which the present study is based | 32 | Funding: Joint Special Project of Guangxi Natural Science Foundation (No. 2025JJH140083); Guangxi Appropriate Medical and Health Technology Development and Promotion Project (S2022138). Funder role: Not specified; databases (TCGA, GEO, IEU GWAS) funding not reported. |
| 19 | **Data and data sharing** | Provide the data used to perform all analyses or report where and how the data can be accessed, and reference these sources in the article. Provide the statistical code needed to reproduce the results in the article, or report whether the code is publicly accessible and if so, where | 32 | Data access: - TCGA-KIRC: https://portal.gdc.cancer.gov/; - GEO GSE53757: https://www.ncbi.nlm.nih.gov/geo/; - IEU GWAS: https://gwas.mrcieu.ac.uk; - Original data: Included in the article; further inquiries to corresponding authors. Statistical code: Not reported as publicly accessible. |
| 20 | **Conflicts of Interest** | All authors should declare all potential conflicts of interest | 32 | Conflicts of Interest: "The authors declare that the research was conducted in the absence of any commercial or financial relationships that could be construed as a potential conflict of interest". |

This checklist is copyrighted by the Equator Network under the Creative Commons Attribution 3.0 Unported (CC BY 3.0) license.

1. Skrivankova VW, Richmond RC, Woolf BAR, Yarmolinsky J, Davies NM, Swanson SA, et al. Strengthening the Reporting of Observational Studies in Epidemiology using Mendelian Randomization (STROBE-MR) Statement. JAMA. 2021;under review.

2. Skrivankova VW, Richmond RC, Woolf BAR, Davies NM, Swanson SA, VanderWeele TJ, et al. Strengthening the Reporting of Observational Studies in Epidemiology using Mendelian Randomisation (STROBE-MR): Explanation and Elaboration. BMJ. 2021;375:n2233.
