# Supplementary material for: UBE2C contributes to malignant phenotypes in clear cell renal cell carcinoma via cell cycle and apoptosis regulation
Source: PeerJ. 2026 Jun 18;14:e21436. doi: 10.7717/peerj.21436 (PMC13283363; doi:10.7717/peerj.21436)
Supplement: Supplemental Information 18 [file peerj-14-21436-s018.docx]

**Supplementary Table S1. Primer sequences used for qRT-PCR.**

| Gene | Forward primer (5′–3′) | Reverse primer (5′–3′) |
| --- | --- | --- |
| UBE2C | 5′-AGGAGCAGCTGAACCCTATT-3′ | 5′-TCCAGGTGCTTCATCACCAT-3′ |
| GAPDH | 5′-GGAGCGAGATCCCTCCAAAAT-3′ | 5′-GGCTGTTGTCATACTTCTCATGG-3′ |
