# Supplementary material for: UBE2C contributes to malignant phenotypes in clear cell renal cell carcinoma via cell cycle and apoptosis regulation
Source: PeerJ. 2026 Jun 18;14:e21436. doi: 10.7717/peerj.21436 (PMC13283363; doi:10.7717/peerj.21436)
Supplement: Supplemental Information 19 [file peerj-14-21436-s019.docx]

**Supplementary Table S2. Antibodies used for Western blot analysis.**

| **Antibodies** | **Art.NO.** | **Ratio** | **Manufacturer** | **Antibodies** | **Art.NO.** | **Ratio** | **Manufacturer** |
| --- | --- | --- | --- | --- | --- | --- | --- |
| **UBE2C** | Ab252940 | 1:1000 | ABCAM | **CDK4** | R23886 | 1:1000 | Zenbio |
| **β- Tubulin** | 10094-1-AP | 1:9000 | Proteintech | **Bcl-2** | R381702 | 1:1000 | Zenbio |
| **Cyclin D1** | R380999 | 1:1000 | Zenbio | **Bax** | R22708 | 1:1000 | Zenbio |
| **P21** | R382492 | 1:1000 | Zenbio | **Cleaved Caspase-3** | 300968 | 1:1000 | Zenbio |
